# High-definition likelihood inference of genetic colocalization reveals protein biomarkers for human complex diseases

**DOI:** 10.1093/gigascience/giaf155

**Published:** 2026-01-23

**Authors:** Yuying Li, Ranran Zhai, Zhijian Yang, Ting Li, Yudi Pawitan, Xia Shen

**Affiliations:** Department of Medical Epidemiology and Biostatistics, Karolinska Institutet, 171 77 Stockholm, Sweden; Center for Intelligent Medicine Research, Greater Bay Area Institute of Precision Medicine (Guangzhou), Fudan University, 511458 Guangzhou, China; Center for Intelligent Medicine Research, Greater Bay Area Institute of Precision Medicine (Guangzhou), Fudan University, 511458 Guangzhou, China; State Key Laboratory of Genetic Engineering, Center for Evolutionary Biology, School of Life Sciences, Fudan University, 200438 Shanghai, China; Center for Intelligent Medicine Research, Greater Bay Area Institute of Precision Medicine (Guangzhou), Fudan University, 511458 Guangzhou, China; Institute for Molecular Medicine Finland (FIMM), HiLIFE, University of Helsinki, 00014 Helsinki, Finland; Center for Intelligent Medicine Research, Greater Bay Area Institute of Precision Medicine (Guangzhou), Fudan University, 511458 Guangzhou, China; State Key Laboratory of Genetic Engineering, Center for Evolutionary Biology, School of Life Sciences, Fudan University, 200438 Shanghai, China; Department of Medical Epidemiology and Biostatistics, Karolinska Institutet, 171 77 Stockholm, Sweden; Department of Medical Epidemiology and Biostatistics, Karolinska Institutet, 171 77 Stockholm, Sweden; Center for Intelligent Medicine Research, Greater Bay Area Institute of Precision Medicine (Guangzhou), Fudan University, 511458 Guangzhou, China; State Key Laboratory of Genetic Engineering, Center for Evolutionary Biology, School of Life Sciences, Fudan University, 200438 Shanghai, China; Centre for Global Health Research, Usher Institute, University of Edinburgh, EH16 4UX Edinburgh, United Kingdom

**Keywords:** HDL-C, genetic colocalization, genetic correlation, proteomics, complex diseases, therapeutic targets

## Abstract

**Background:**

Genetic colocalization analysis is essential for understanding the shared genetic basis between phenotypic traits. Such an analysis is particularly useful for identifying plasma proteins with potential as therapeutic targets or clinical biomarkers. Improvements to existing tools are needed for more accurate inference of potentially causal biomarkers.

**Findings:**

We develop high-definition likelihood for colocalization inference (HDL-C), a method for genetic colocalization analysis. Based on simulations and observed rediscovery rates in real data analyses, we demonstrate that the HDL-C approach outperforms state-of-the-art methods, COLOC, SuSiE, and SharePro, in detecting genetic colocalization, thus enabling a more complete understanding of genetic connections at specific loci. Analyses of the top 50 protein–disease pairs identified by HDL-C in the male and female cohorts of the UK Biobank uncovered 40 previously validated drug-protein–disease combinations with approved drugs matching the phenotypes and 62 combinations with potential drug re-purposing opportunities. Additionally, we identified 63 novel protein–disease pairs that suggest promising candidates for future therapeutic interventions.

**Conclusion:**

This research establishes a robust framework for detecting genetic colocalization signals, enabling the prioritization of disease-relevant protein targets and informing therapeutic development strategies.

## Introduction

Genetic influences underlying human diseases and traits remain an important area of investigation in genomics. Genome-wide association studies (GWAS) have significantly advanced genetic research by identifying numerous genomic regions linked to various traits and disease susceptibilities [1–5]. A key aspect of this exploration is understanding how variations in the genome correlate with variations in phenotypic traits, including those associated with complex diseases. This understanding not only reveals the genetic architecture of these traits but also helps to identify potential therapeutic targets and biomarkers for disease prediction and management.

Plasma proteins, given their critical roles in various biological processes and disease pathways, serve as valuable biomarkers and therapeutic targets. The measured proteome encompasses proteins secreted or shed into the blood circulation, which play major roles in various molecular processes and mediate cross-tissue communication [6]. Their expression levels, often influenced by genetic variations, can provide insights into the molecular mechanisms of diseases. Recent technological advancements in high-throughput quantification of circulating proteins have led to large-scale studies of protein quantitative trait loci (pQTL) [7–13]. These studies have highlighted the potential of associating protein levels with DNA sequence variants that colocalize with risk alleles for common diseases. Such colocalizations can reveal disease-associated pathways, offering novel insights into drug targets and translational biomarkers.

Therefore, methods for more accurate inference of genetic colocalization are essential for the joint analysis of molecular traits and complex diseases. COLOC is one of the most widely used methods for colocalization analysis, which aims to detect genetic colocalization between pairs of traits, such as the analysis of complex traits at specific molecular quantitative trait loci (QTL) [14]. This Bayesian model makes restrictive assumptions about the underlying shared genetic architecture at the given locus (e.g., 1 causal variant per trait). Such an assumption may not always hold in real-world datasets. While extensions of COLOC [15] using conditional regression have been attempted to address the issue of multiple variants, they rely on assumptions of independence among causal variants, which may not hold true, especially in the presence of extensive linkage disequilibrium (LD) [16, 17]. The Sum of Single Effects (SuSiE) framework, integrated into the COLOC package, has addressed these limitations by enabling robust fine-mapping of multiple causal variants [18–20]. However, this approach primarily focuses on fine-mapping rather than quantifying colocalization itself. A more recent method is SharePro, which explicitly models multiple causal variants and joint fine-mapping [21].

As an alternative strategy, we propose the inference of a *sufficiently high* estimated regional genetic correlation (${{r}_G}$) between 2 phenotypes at a specific genomic locus to detect genetic colocalization. Unlike existing methods, this strategy quantifies colocalization through a single genetic correlation parameter without strict assumptions about the underlying genetic architecture. We previously developed the high-definition likelihood (HDL) method as a robust approach for estimating genetic correlations using GWAS summary statistics [22] and the recent local version of this method, HDL-L, to estimate local genetic correlations [23]. This advancement enables more granular exploration of genetic correlations at specific loci. Nevertheless, for colocalization detection, inference must be based on a conditional likelihood given a sufficiently high regional genetic correlation estimate.

In this study, we (i) develop the theory and implement high-definition likelihood for colocalization inference (HDL-C); (ii) demonstrate that HDL-C performs better than COLOC, SuSiE, and SharePro in detecting genetic colocalization; and (iii) apply the HDL-C method to investigate the colocalization between plasma proteins and complex diseases using data from the UK Biobank. Specifically, we prioritize drug targets focusing on 2,826 plasma proteins and their colocalization with 200 diseases. This approach offers a new opportunity to explore the genetic basis of disease–protein associations, potentially uncovering novel insights into disease mechanisms.

## Results

### Overview of the HDL-C method

We define regional genetic colocalization as the presence of a non-zero local genetic correlation between 2 traits. When the estimated correlation ${{r}_G}$ equals zero, the local genetic effects are uncorrelated, and there is no evidence of colocalization. Conversely, a significantly large ${{r}_G}$ indicates that the traits share a consistent pattern of genetic effects within the region. In practice, one may regard a region as colocalized either when ${{r}_G}$ is significantly different from zero or exceeds a prespecified threshold ${{r}_0} > 0$ that reflects a biologically meaningful level of correlation.

To formally test whether the local genetic correlation exceeds such a threshold, we develop the **HDL-C** method—a constrained likelihood ratio framework built upon the high-definition likelihood model to test genetic colocalization.

By definition, ${{r}_G} = {{h}_{12}}/\sqrt {h_1^2h_2^2} $, where $h_1^2$ and $h_2^2$ denote the local single-nucleotide polymorphism (SNP) heritabilities of the 2 traits, and ${{h}_{12}}$ is their local genetic covariance. We consider the likelihood function $\mathcal{L}( {h_1^2,h_2^2,{{h}_{12}}\mid {{{{\bf z}}}_1},{{{{\bf z}}}_2}} )$ for the regional genetic association *Z*-scores $( {{{{{\bf z}}}_1},{{{{\bf z}}}_2}} )$, derived under the HDL multivariate normal model. We test whether the magnitude of the local genetic correlation exceeds a biologically meaningful threshold ${{r}_0}\in[ {0,1} )$:


\begin{eqnarray*}
{{H}_0}:\left| {{{r}_G}} \right| \le {{r}_0}\,\, \Leftrightarrow \,\, \left| {{{h}_{12}}} \right| \le {{r}_0}\sqrt {h_1^2h_2^2} ,\quad {{H}_A}:\left| {{{r}_G}} \right| > {{r}_0}.
\end{eqnarray*}

The null hypothesis, therefore, defines a bounded composite region in the parameter space. The *profile* likelihood of the genetic covariance is ${{\mathcal{L}}_p}( {{{h}_{12}}} ) = {\mathrm{ma}}{{{\mathrm{x}}}_{h_1^2,h_2^2}}\mathcal{L}( \theta ) = \mathcal{L}( {{{h}_{12}},\hat{h}_1^2,\hat{h}_2^2} )$, where $\hat{h}_1^2$ and $\hat{h}_2^2$ are the maximum likelihood estimates (MLEs) of the heritabilities. The LRT statistic for genetic covariance is formulated as


\begin{eqnarray*}
{\mathrm{\Lambda }} = - 2{\mathrm{ln}}\left[ {\frac{{{\mathrm{sup}}{{\mathcal{L}}_p}\left( {{{h}_{12}}} \right):\left| {{{h}_{12}}} \right| \le {{r}_0}\sqrt {\hat{h}_1^2\hat{h}_2^2} }}{{{\mathrm{sup}}{{\mathcal{L}}_p}\left( {{{h}_{12}}} \right):\left| {{{h}_{12}}} \right| \le \sqrt {\hat{h}_1^2\hat{h}_2^2} }}} \right].
\end{eqnarray*}

In practice, we profile over ${{h}_{12}}$ while fixing $h_1^2$ and $h_2^2$ at their unconstrained MLEs, which preserves the null constraint $| {{{h}_{12}}} | \le B$, where $B = {{r}_0}\sqrt {\hat{h}_1^2\hat{h}_2^2} $. Because the null involves an inequality constraint, the asymptotic null distribution of ${\mathrm{\Lambda }}$ follows a mixture of


\begin{eqnarray*}
{\mathrm{\Lambda }}\overset{H_{0}}{\rightarrow} 1/2\chi _0^2 + 1/2\chi _1^2,
\end{eqnarray*}

that is, a 50:50 mixture of a point mass at zero and a $\chi _1^2$ distribution [24].

This procedure directly tests whether the local genetic correlation exceeds a biologically meaningful threshold ${{r}_0}$, rather than testing for zero correlation. It can equivalently be viewed as assessing whether the profile-likelihood confidence interval for ${{r}_G}$ lies entirely outside the interval $[ { - {{r}_0},{{r}_0}} ]$. In this sense, HDL-C provides a likelihood-based test of regional colocalization strength, complementing HDL-L, which estimates the magnitude of local correlation.

In contrast to standard colocalization methods, which typically model variant-level causal probabilities under strong prior assumptions, HDL-C exploits the summary-level multivariate Gaussian structure of *Z*-scores and the polygenic covariance encoding in the LD score matrix. HDL-C thus provides a high-dimensional, likelihood-based inference procedure for genetic colocalization that requires only GWAS summary statistics and an LD reference.

In practice, the choice of ${{r}_0}$ reflects the minimum degree of local genetic sharing required to declare colocalization. In biomarker discovery, where near-identical genetic architectures are desired, we recommend a conservative range of ${{r}_0}\in0.5,0.8$; for exploratory scans allowing partial sharing, ${{r}_0} \approx 0$ is reasonable. Based on simulations, the empirical performance of HDL-C was generally robust across choices of ${{r}_0}$, with only minor power gains observed for ${{r}_0} = 0$ under low genetic correlations. Therefore, we suggest reporting results from ${{r}_0} = 0.5$ as a balanced default, accompanied by *P* values and likelihood-based estimates of local genetic correlation to jointly assess statistical significance and biological concordance.

To evaluate the performance of HDL-C, we conducted a series of simulation studies comparing it with COLOC [14], SuSiE [20], and SharePro [21]. Given that COLOC inherently assumes a single causal SNP per region, we designed simulations in 2 scenarios: (i) each region had a single causal SNP, and (ii) each region was simulated under multiple causal-variant scenarios, assuming 3%, 5%, or 10% of SNPs as causal. In addition, to analyze protein molecules and complex diseases, we examined different levels of true regional heritability for the disease trait. For *cis*-pQTL, we estimated the heritability of the top SNP in each *cis*-region (see Methods) and then randomly selected 300 *cis*-pQTL reflecting the heritability distribution of the full set of 2,826 *cis*-pQTL (Supplementary Fig. S1). This subset approach was used to manage computational efficiency, as conducting simulations in all pQTL regions would be excessively computationally intensive. In each simulation replicate, we generated phenotypic data for 2 traits and estimated their local genetic correlation. The true effect sizes of the causal variants were drawn from a bivariate normal distribution, given the true genetic correlation (see Methods). The summary association statistics were then calculated from a genome-wide association analysis by regressing the simulated phenotypic data against the corresponding genotypes at each SNP.

### HDL-C outperforms state-of-the-art methods in detecting genetic colocalization

**Figure 1:**
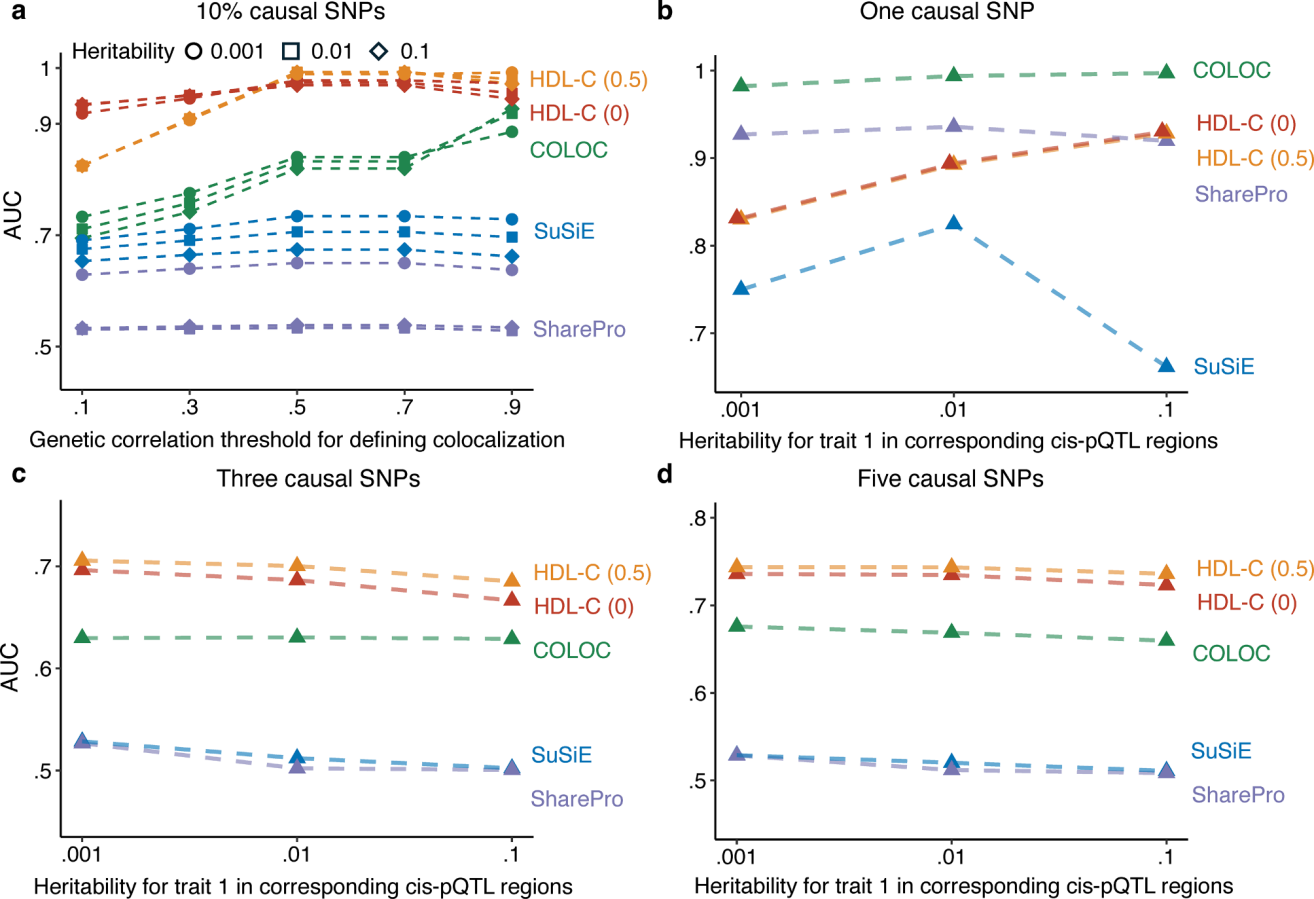
Performance comparison of HDL-C, COLOC, SuSiE, and SharePro in detecting genetic colocalization by AUC. Panels show the area under the ROC curve (AUC) for different methods across simulation settings. The colocalization level was simulated across different true genetic correlation values. (a) Results for 10% causal SNPs, plotted against the genetic correlation threshold used to define colocalization. We examined 3 different levels of true regional heritability for disease traits corresponding to *cis*-pQTL regions. (b–d) Results for 1, 3, and 5 causal SNPs, respectively. HDL-C (0) refers to the setting in which colocalization is assessed by testing whether the local genetic correlation equals zero (${{{\boldsymbol{r}}}_{\boldsymbol{G}}} = 0$), whereas HDL-C (0.5) corresponds to a more stringent criterion that tests whether the local genetic correlation is less than or equal to 0.5 (${{{\boldsymbol{r}}}_{\boldsymbol{G}}} \le 0.5$).

Under the assumption of 10% causal SNPs, HDL-C consistently outperformed COLOC, SuSiE, and SharePro across a range of true genetic correlation thresholds (0–1) and regional heritability levels (Fig. 1A). We evaluated 2 HDL-C thresholds, denoted HDL-C (0) and HDL-C (0.5), corresponding to increasingly stringent definitions of colocalization. The area under the curve (AUC) of HDL-C (0) ranged from 0.92 to 0.98, compared with 0.82 to 0.99 for HDL-C (0.5), 0.70 to 0.93 for COLOC, 0.65 to 0.73 for SuSiE, and 0.53 to 0.65 for SharePro. More specifically, HDL-C achieved higher true-positive rates (TPRs) than COLOC, SuSiE, and SharePro at both 5% and 10% false-positive rate (FPR) thresholds (Supplementary Fig. S2). Under the single-causal-variant setting, where true local genetic correlation is expected to be $\pm 1$ when colocalization exists, COLOC achieved the highest AUC, followed by SharePro, HDL-C, and SuSiE (Fig. 1B). When regional heritability for the disease trait increased to 0.1 in the corresponding *cis*-pQTL region, HDL-C performed comparably to SharePro, with AUCs of 0.93 and 0.92, respectively. In scenarios with 3 and 5 causal variants (Fig. 1C, D), HDL-C again delivered the strongest overall performance across all heritability levels. Notably, when the number of causal variants increased and the true genetic correlation weakened, HDL-C (0) outperformed the more conservative HDL-C (0.5). To assess computational efficiency, we benchmarked per-locus execution time across 50 simulation replicates in 300 *cis*-pQTL regions (15,000 runs per method; Supplementary Fig. S3). All analyses were performed on a single CPU core without parallelization, using a uniform memory allocation of 8 GB for all methods. Median runtime was 0.007 seconds for COLOC, 0.445 seconds for SuSiE, 2.04 seconds for HDL-C, and 4.93 seconds for SharePro. Although HDL-C is not the fastest, it completes within a few seconds per locus and exhibits stable upper-tail performance (95th percentile $< $3 seconds). At this rate, analysis of 1,000 loci requires approximately 34 minutes on a single core. The speed of COLOC reflects its simpler single-causal-variant model.

### HDL-C has higher rediscovery rates in 2 independent samples

**Figure 2:**
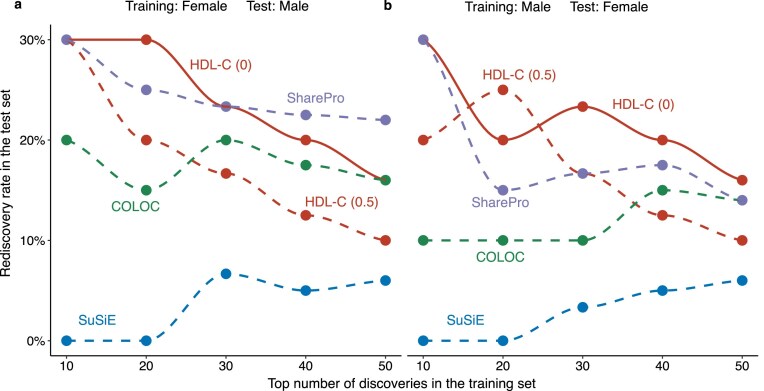
Rediscovery rates of HDL-C, COLOC, SuSiE, and SharePro under 2 validation settings. (a) Training was performed on the female dataset, and testing was performed on the male dataset. (b) Training was performed on the male dataset and testing on the female dataset. The x-axis shows the top N significant results selected in the training set. The y-axis shows the proportion of these signals rediscovered in the test set. We applied HDL-C, COLOC, SuSiE, and SharePro on 200 ICD-10–coded diseases and 2,826 protein summary association statistics in the UK Biobank male and female population. HDL-C (0) refers to the setting in which colocalization is assessed by testing whether the local genetic correlation equals zero (${{{\boldsymbol{r}}}_{\boldsymbol{G}}} = 0$), whereas HDL-C (0.5) corresponds to a more stringent criterion that tests whether the local genetic correlation is less than or equal to 0.5 (${{{\boldsymbol{r}}}_{\boldsymbol{G}}} \le 0.5$).

To demonstrate our theory in real data analyses, we evaluated their ability to detect genetic colocalization between 200 International Classification of Diseases, 10th Revision (ICD-10)–coded diseases and 2,826 proteins in the UK Biobank. We focused on *cis*-pQTL regions to explore the shared genetic architecture between these diseases and proteins in male and female populations. We used 2 validation settings. In the first setting, we used female data for training and male data for testing. In the second setting, we used male data for training and female data for testing. This design allowed us to directly evaluate the reproducibility of findings across sex-stratified cohorts. We provide detailed descriptions of the diseases and their associated proteins in Supplementary Tables S2–S3. The top 50 significant colocalization results from HDL-C, COLOC, SuSiE, and SharePro analyses were selected from the training set, and we examined the rediscovery rates (RDRs) for these methods in the test set as the proportion of overlapping results between the top 50 findings in the test and training sets (Fig. 2, Supplementary Table S4).

HDL-C (0) achieved the highest rediscovery rates among all evaluated methods, except in the female-to-male validation, when the top discoveries exceeded 40, where SharePro performed slightly better (Fig. 2). Based on simulation results, this exception likely reflects that shared causal variants between males and females are often driven by a single dominant causal SNP rather than multiple shared signals. Overall, HDL-C (0.5) performed worse than HDL-C (0), indicating that most protein–disease associations exhibit weak and highly polygenic genetic architectures. Some diseases may have sex-specific patterns: This would influence the RDR [25, 26] but not affect the comparison of performance between the methods. Also, in our sex-stratified GWAS analyses, the first 20 genetic principal components (PCs) were included to account for population structure. These PCs were derived from the entire UK Biobank cohort using genome-wide genotype data, making it unlikely that the observed local genetic correlations were artifacts of population stratification. Taken together, HDL-C (0) demonstrated superior reproducibility and robustness under these validation settings. COLOC and SuSiE could identify overlapping genetic loci but often yielded lower RDR compared to HDL-C. SharePro achieved intermediate performance across both validation directions. This finding showed the advantage of HDL-C’s likelihood-based framework in detecting robust colocalization when applied to real data.

### HDL-C prioritizes drug targets for human complex diseases

**Figure 3:**
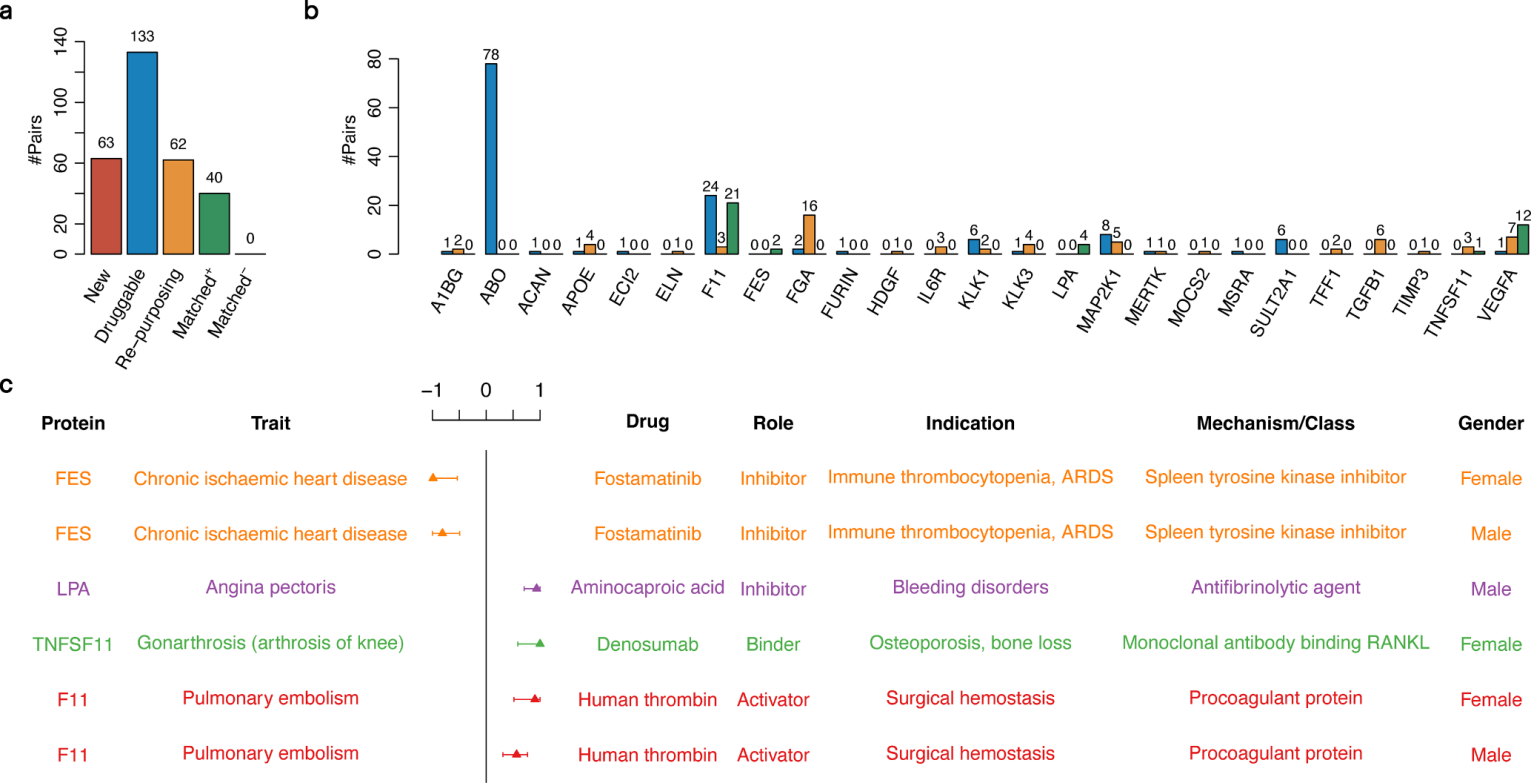
Drug targets inferred by local genetic correlation analysis. (a) Drug-protein–phenotype combinations identified from the top 50 HDL-C results in separate male and female cohorts, grouped into 4 categories: “New,” representing novel protein–disease associations with no existing drugs; “Druggable,” where proteins are under clinical evaluation or considered viable for small-molecule development; “Re-purposing,” where existing drugs are approved for different diseases; and “Validated,” where known drugs affect both the protein and the disease. Validated combinations are further subdivided into “Matched +,” where the drug’s effect direction aligns with the HDL-C estimation, and “Matched −,” where the effect direction differs from the HDL-C findings. (b) Number of distinct categories per protein. (c) Representative examples of validated known targets, including drug descriptions, their primary indications or side effects, and HDL-C effect estimates. The local genetic correlation estimates are shown as filled triangles with $95{\boldsymbol{\% }}$ confidence intervals (whiskers).

We extended our analysis by investigating the top 50 genetically correlated protein–disease pairs identified by HDL-C (0) in the male and female subcohorts, respectively, resulting in 92 unique protein–disease combinations. Each of these combinations was cross-referenced with DrugBank (Supplementary Table S5). Integrating HDL-C discoveries with existing drug information, 40 validated drug-protein–disease combinations were identified, where a given drug targets the same protein and treats the same disease or causes the same side effect (“Matched”). For all of the 40 matched combinations, the HDL-C inferred protein’s causal effect directions were consistent with the corresponding drug action direction (“Matched^+^”) (Fig. 3a). We also identified 62 combinations where the drugs have different approved indications that differ from the diseases or side effects identified in the HDL-C results (“Re-purposing”), suggesting potential re-purposing opportunities. Furthermore, we discovered 63 protein–disease pairs where the proteins are not targeted by any drug in DrugBank (“New”), indicating potential novel therapeutic targets if the potential causal effects can be validated. In addition, we denoted 133 combinations as “Druggable,” which means their ongoing evaluation in clinical trials or their viability for development into small-molecule therapies. We further showed the distribution of these drug-protein–phenotype combinations per protein (Fig. 3b).

For example, we observed that tyrosine-protein kinase Fes/Fps (FES, UniProt P07332) exhibited a protective effect against chronic ischemic heart disease (ICD-10: I25), as indicated by a significant local genetic correlation estimate of −0.99 (95% CI, −0.54 to −1.00) in females and −0.81 (95% CI, −0.49 to −1.00) in males (Fig. 3c). Fostamatinib—marketed as Tavalisse since its approval by the US Food and Drug Administration on 17 April 2018—was developed as a spleen tyrosine kinase (SYK) inhibitor for rheumatoid arthritis and immune thrombocytopenic purpura (ITP). However, studies have demonstrated that its active metabolite (R406) can also inhibit FES, a kinase implicated in the regulation of protective inflammation [27]. While fostamatinib’s anti-inflammatory properties have been investigated for mitigating vascular damage (and even acute respiratory distress syndrome in severe COVID-19), recent clinical evidence points to an increased incidence of cardiovascular side effects, notably hypertension, that may exacerbate ischemic heart conditions. This aligns with our findings of genetic correlation.

Aminocaproic acid is an antifibrinolytic agent that, by inhibiting plasminogen activation, may potentiate the prothrombotic environment in individuals with elevated apolipoprotein(a) (Lp(a)). Higher Lp(a) levels themselves are well-documented risk factors for atherosclerotic disease, including angina pectoris, due to Lp(a)’s structural similarity to plasminogen and resultant interference with normal fibrinolysis. Thus, when aminocaproic acid further restricts fibrinolysis, it can intensify the cardiovascular risk posed by elevated Lp(a), leading to an increased incidence or severity of angina pectoris. This mechanistic interplay aligns with our significant local genetic correlation finding (0.94 with 95% CI, 0.70–1.00) between Lp(a) and angina pectoris (Fig. 3c), underscoring the shared risk pathway involving fibrinolysis inhibition and Lp(a)-related atherogenesis.

TNF superfamily member 11 (TNFSF11) displayed a risk-increasing effect on gonarthrosis (arthrosis of the knee) disease (ICD-10: M17), with a significant local genetic correlation estimation of 1.00 (95% CI, 0.59–1.00) (Fig. 3c). Gonarthrosis is marked by both progressive cartilage breakdown and pathologic remodeling of the subchondral bone. Increasing evidence showed that the RANK–RANKL–OPG axis is a key mediator in this process, with elevated RANKL driving osteoclast activity and contributing to aberrant bone turnover in osteoarthritis [28, 29]. Experimental studies using *in vitro* and animal models suggest that inhibiting RANKL can reduce excessive osteoclast-mediated resorption in the subchondral bone, potentially slowing disease progression [30, 31]. Denosumab, a human monoclonal antibody targeting RANKL, effectively suppresses osteoclast formation and bone resorption and is currently approved for osteoporosis and skeletal metastases [32]. Although its use in arthrosis of the knee remains to be verified through a large-scale clinical trial, these findings provide a plausible rationale for exploring RANKL inhibition as part of a disease-modifying strategy in osteoarthritis management. In summary, these findings demonstrate how integrating genetic correlation signals with drug databases can pinpoint both established and emergent therapeutic opportunities, particularly for complex disorders.

**Figure 4:**
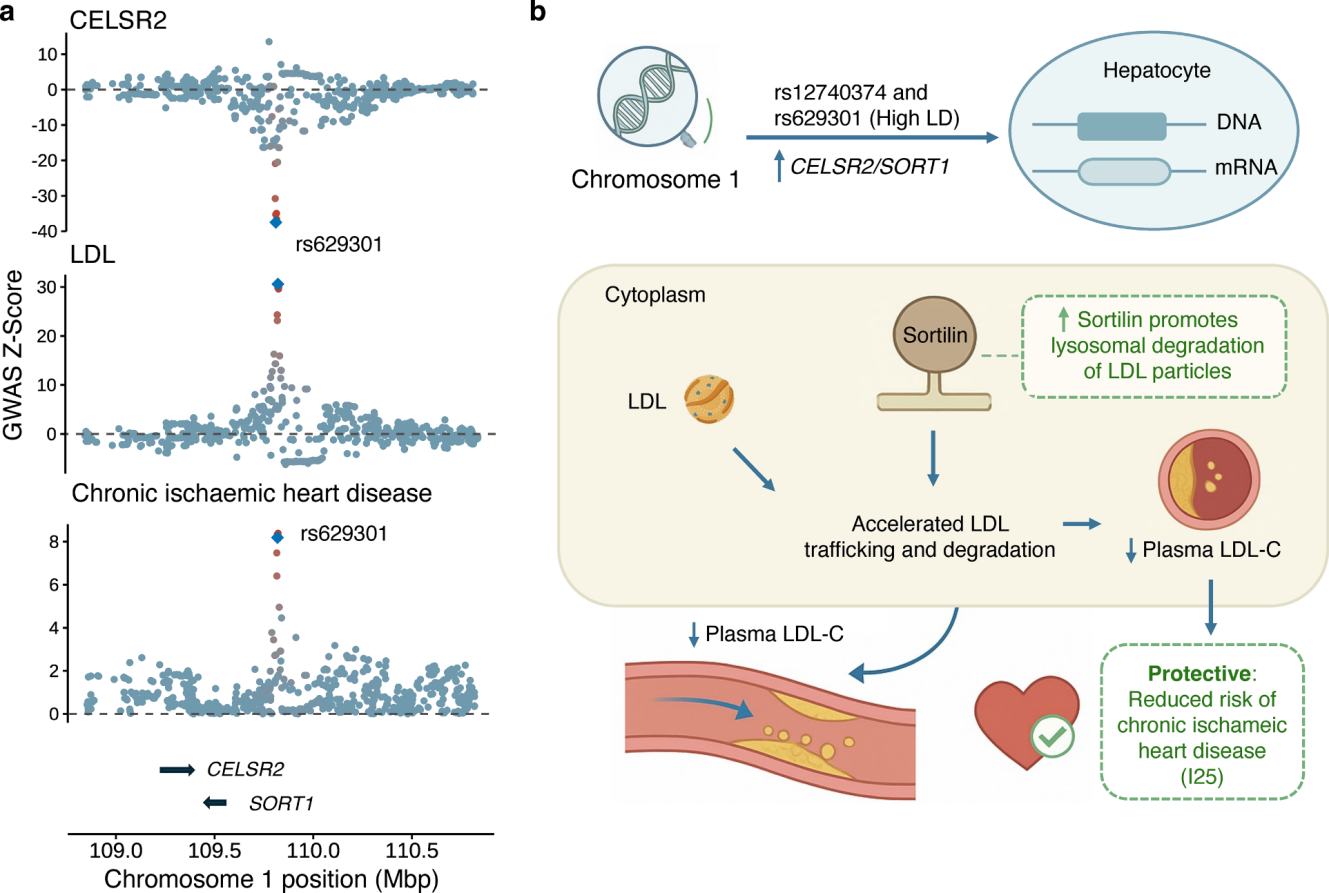
Potential new therapeutic target for chronic ischemic heart disease. (a) Regional association plots around *CELSR2* showing the *cis*-pQTL signal for plasma CELSR2 protein (top), the GWAS signal for LDL (middle), and the GWAS signal for chronic ischemic heart disease (bottom). The blue diamond shows the sentinel pQTL variant. Other variants are colored by LD to the sentinel pQTL. (b) Schematic illustration depicting the role of rs629301 in modulating CELSR2 expression and its downstream impact on LDL-C levels and ischemic heart disease (I25) risk.

Among the 63 newly identified protein–disease pairs, for instance, the HDL-C analysis found that the cadherin EGF LAG seven-pass G-type receptor 2 (CELSR2) was a potential target for chronic ischemic heart disease (ICD-10: I25). In both sexes, the local genetic correlation was nearly −1 (−0.94 in males [95% CI, −0.74 to −1.00] and −1.00 in females [95% CI, −0.62 to −1.00]), driven by rs629301, a variant tightly linked to the well-studied rs12740374. The lead variant rs629301 was associated with CELSR2 protein levels in plasma, indicating a regulatory effect. This modulation likely influenced low-density lipoprotein (LDL) cholesterol levels and contributed to ischemic heart disease risk (Fig. 4a). Existing studies indicate that rs12740374 modulates the hepatic expression of CELSR2 and its neighboring gene sortilin 1 (SORT1), affecting LDL cholesterol levels and coronary event risk. Functional analyses in human hepatocytes and mouse models have shown that elevated sortilin accelerates intracellular trafficking and lysosomal degradation of APOB-containing lipoproteins, reducing atherogenic particle pools [33, 34] (Fig. 4b). These findings suggest CELSR2 as a potential therapeutic target for lipid-lowering therapies in chronic ischemic heart disease.

We further assessed whether the top-ranked HDL-C discoveries in the RDR analysis were preferentially enriched for protein–trait pairs supported by prior experimental or clinical evidence. To address this, we examined the composition of annotated categories across increasing top-*N* discoveries. The results showed that the enrichment for evidence-supported pairs (Matched+) was the highest among the most highly ranked signals and decreased monotonically as *N* increased. This indicated that the strongest, most validated signals were concentrated near the top of the ranking (Supplementary Fig. S4).

## Discussion

We introduced HDL-C, a new extended approach built upon the high-definition likelihood (HDL-L) framework to infer colocalization based on sufficiently high genetic correlation. HDL-L tests for the existence of nonzero local genetic covariance. However, nonzero covariance does not necessarily imply a shared causal architecture: weak or diffuse correlations may arise from LD structure, polygenicity, or multiple distinct causal variants. In contrast, colocalization requires that the genetic effects for 2 traits proportionally align across the underlying causal variants, a condition that is theoretically equivalent to the local genetic correlation exceeding a meaningful threshold. Our analyses indicate that HDL-C outperforms COLOC, SuSiE, and SharePro in detecting genetic colocalization in simulated and real datasets. In the top 50 findings for male and female UK Biobank subcohorts, HDL-C not only demonstrated robust efficacy but also identified previously unrecognized genetic associations between plasma proteins and diseases. The identification of these colocalized protein–disease pairs helped understand the genetic basis of complex diseases. The results prioritized novel protein targets for further investigation, which might lead to the development of new therapeutic strategies and clinical biomarkers.

From a methodological perspective, COLOC employs summary statistics with prior probabilities to infer whether 2 traits share a causal variant. This method distinguishes among multiple hypotheses in a Bayesian framework, notably H3 (indicating distinct causal variants for the 2 traits) and H4 (indicating a shared causal variant for both traits). Although COLOC’s reliance on prior probabilities enhances flexibility, it also introduces potential bias if default priors are arbitrarily selected and not validated by sensitivity analyses. Furthermore, COLOC does not model multiple causal variants simultaneously, limiting its utility in regions of high LD, where distinguishing competing hypotheses (H3 vs. H4) becomes challenging. Thus, careful application and sensitivity analyses are essential to ensure robust conclusions.

SuSiE enhances colocalization inference by explicitly modeling multiple causal signals, improving accuracy over single-variant methods. However, its performance depends critically on high-quality LD estimates. Mismatches between the LD reference panel and the study population can lead to spurious signals. Additionally, selecting the parameter L (the number of allowed causal effects) requires consideration: underestimating L risks missing true signals, while overestimating L may fragment true signals or overfit noise. In our simulations, the default L setting was adopted.

HDL-C addresses these limitations by detecting sufficiently high local genetic correlation using GWAS summary statistics while accounting for LD. This approach captures associations between genetic effect vectors across traits, independent of the number of causal variants. For example, a shared causal variant with pleiotropic effects induces proportional effect estimates across SNPs in LD, producing a strong local genetic correlation (approaching $\pm $1.0 depending on the directionality of the effect). Conversely, unshared distinct causal variants produce low correlations, reflecting independent signals. Another intuitive advantage is that it inherently accounts for the direction and magnitude of effects, not just their existence.

Our comparison between ${{r}_0} = 0$ and ${{r}_0} = 0.5$ of HDL-C showed similar AUC across simulation scenarios, indicating that HDL-C is generally robust to the choice of colocalization threshold. However, under polygenic architectures with weak underlying genetic correlations, HDL-C (0) (equivalent to the unconstrained HDL-L test) would demonstrate slightly higher power. Nevertheless, HDL-C is built upon the high-definition likelihood (HDL-L) framework, with the extension aimed at addressing the specific inferential goal of detecting colocalization rather than general genetic correlation estimation. HDL-L and related approaches estimate or test the existence of nonzero genetic covariance between 2 traits within a region. In contrast, colocalization usually aims to detect whether 2 traits share the same causal architecture within a locus (i.e., whether the genetic effects at the shared causal variants *proportionally align* between the 2 traits). This theoretically corresponds to a sufficiently strong local genetic correlation (i.e., $| {{{r}_G}} |$ exceeds a threshold). HDL-C (with nonzero ${{r}_0}$) tests for a large $| {{{r}_G}} |$ threshold (rather than ${{h}_{12}}\neq 0$). This can avoid reporting situations where small but nonzero covariances are misinterpreted as evidence of colocalization.

In practice, the choice of ${{r}_0}$ should reflect the scientific definition of “colocalization” for the analysis at hand. In biomarker discovery, we typically require near-identical local architectures, so we recommend a conservative default of ${{r}_0}\in[ {0.5,0.8} ]$. For exploratory scans where partial sharing is acceptable, even ${{r}_0} \approx 0$ is reasonable. We suggest 2 data-driven options: (i) rediscovery calibration (e.g., sex-stratified or sample-split replicates) by picking ${{r}_0}$ that maximizes rediscovery at a certain number of discoveries or (ii) choosing ${{r}_0}$, giving a desired empirical false-positive rate using negative controls (trait pairs expected not to share biology or permuted/proxy regions).

We suggest that the RDR should be a standard criterion for validating statistical discovery methods. It is a function that considers both the false-positive rate and power in both the training and validation samples [35], which measures the probability that a declared discovery reappears upon replication. Methods that suffer from inflated type I error often produce nonreplicable signals, leading to a low rediscovery rate. The RDR analysis is a useful design for evaluating methods based on real data, which incorporates realistic information and complications that simulations cannot capture. In the particular RDR analysis of this article, for most top discoveries, HDL-C (0) actually reported more robust/replicable results than HDL-C (0.5), indicating that (i) multiple causal variants are shared between proteins and traits, and (ii) the shared (replicable) local genetic correlations between males and females do not have great magnitudes. Nevertheless, this finding currently only applies to the UK Biobank cohort, and future studies may be needed to validate.

This work presents a novel perspective on colocalization analysis, especially offering a better understanding of the genetic colocalization between human plasma proteins and complex traits. The insights gained from this study are not only valuable for genetics research but also have broad implications for the fields of personalized medicine and drug development.

## Methods

### Theory of the HDL-C method

The HDL-C builds upon the likelihood formulation of local genetic covariance under the bivariate HDL framework [23]. The method provides a likelihood ratio test (LRT) under a constrained null hypothesis for determining whether the local genetic correlation between 2 traits exceeds a biologically meaningful threshold.

For 2 traits with GWAS summary *Z*-score vectors ${{{{\bf z}}}_1}$ and ${{{{\bf z}}}_2}$ measured at *M* SNPs in a given LD block, HDL-C assumes a bivariate Gaussian model:


\begin{eqnarray*}
\left( {\begin{array}{@{}*{1}{c}@{}} {{{{{\bf z}}}_1}}\\ {{{{{\bf z}}}_2}} \end{array}} \right) \sim \mathcal{N}\left( {0,\left( {\begin{array}{@{}*{2}{c}@{}} {{{{{\bf \Sigma }}}_{11}}}&{{{{{\bf \Sigma }}}_{12}}}\\ {{{\bf \Sigma }}_{12}^ \top }&{{{{{\bf \Sigma }}}_{22}}} \end{array}} \right)} \right),
\end{eqnarray*}

where ${{{{\bf \Sigma }}}_{ii}}$ and ${{{{\bf \Sigma }}}_{12}}$ denote the within-trait and cross-trait covariance structures of *Z*-scores, respectively. Specifically,


\begin{eqnarray*}
{{{{\bf \Sigma }}}_{ii}} = \frac{{{{N}_i}h_i^2}}{M}{{\bf L}} + {{\bf R}},\quad {{{{\bf \Sigma }}}_{12}} = \frac{{\sqrt {{{N}_1}{{N}_2}} {{h}_{12}}}}{M}{{\bf L}},
\end{eqnarray*}

where ${{N}_i}$ is the sample size of trait *i*, $h_i^2$ denotes the local SNP heritability, ${{h}_{12}}$ is the local genetic covariance, ${{\bf R}}$ is the LD correlation matrix, and ${{\bf L}} = {{{{\bf R}}}^2}$ is the LD score matrix. This parameterization captures both sampling variance and LD-induced correlation between SNPs. The local genetic correlation is defined as


\begin{eqnarray*}
{{r}_G} = \frac{{{{h}_{12}}}}{{\sqrt {h_1^2h_2^2} }},
\end{eqnarray*}

bounded by $[ { - 1,1} ]$. HDL-C aims to test whether the magnitude of ${{r}_G}$ exceeds a pre-specified colocalization threshold ${{r}_0}\in[ {0,1} )$:


\begin{eqnarray*}
{{H}_0}:\left| {{{r}_G}} \right| \le {{r}_0}\quad \Leftrightarrow \quad \left| {{{h}_{12}}} \right| \le {{r}_0}\sqrt {h_1^2h_2^2} ,\quad{{H}_A}:\left| {{{r}_G}} \right| > {{r}_0}.
\end{eqnarray*}

Let $\mathcal{L}( \theta )$ denote the full likelihood function with parameters $\theta = ( {h_1^2,h_2^2,{{h}_{12}}} )$. To construct the likelihood ratio test, we first estimate the trait-specific heritabilities via maximum likelihood $( {\hat{h}_1^2,\hat{h}_2^2} )$ under the HDL model, treating them as nuisance parameters. Then the profile likelihood of the genetic covariance is


\begin{eqnarray*}
{{\mathcal{L}}_p}\left( {{{h}_{12}}} \right) = \mathop {{\mathrm{max}}}\limits_{h_1^2,h_2^2} \mathcal{L}\left( {h_1^2,h_2^2,{{h}_{12}}} \right) = \mathcal{L}\left( {{{h}_{12}},\hat{h}_1^2,\hat{h}_2^2} \right).
\end{eqnarray*}

The LRT statistic is given by


\begin{eqnarray*}
{\mathrm{\Lambda }} = - 2{\mathrm{ln}}\left[ {\frac{{{\mathrm{sup}}{{\mathcal{L}}_p}\left( {{{h}_{12}}} \right):\left| {{{h}_{12}}} \right| \le {{r}_0}\sqrt {\hat{h}_1^2\hat{h}_2^2} }}{{{\mathrm{sup}}{{\mathcal{L}}_p}\left( {{{h}_{12}}} \right):\left| {{{h}_{12}}} \right| \le \sqrt {h_1^2h_2^2} }}} \right].
\end{eqnarray*}

Because the null hypothesis imposes an inequality constraint on ${{h}_{12}}$, the asymptotic null distribution of ${\mathrm{\Lambda }}$ follows a chi-bar-square mixture [24]:


\begin{eqnarray*}
{\mathrm{\Lambda }}\overset{H_{0}}{\rightarrow} 1/2\chi _0^2 + 1/2\chi _1^2.
\end{eqnarray*}

Accordingly, a conservative *P* value can be computed as $p = 1/2\,{\mathrm{Pr}}( {\chi _1^2 \ge {\mathrm{\Lambda }}} )$.

This formulation directly tests whether the estimated local genetic correlation exceeds ${{r}_0}$, rather than testing for zero correlation. Inference is equivalent to checking whether the likelihood-based confidence interval for ${{r}_G}$ lies entirely outside the interval $[ { - {{r}_0},{{r}_0}} ]$. In our analyses, we consider 2 settings of the HDL-C method (under ${{r}_0} = 0$ and ${{r}_0} = 0.5$), where HDL-C (0) corresponds to the standard local genetic correlation testing method HDL-L.

Compared with standard Bayesian colocalization methods, such as COLOC [14] or SuSiE [20], which rely on variant-level causal priors and enumeration of causal configurations, HDL-C operates purely at the regional level. By exploiting the multivariate normal structure of GWAS summary *Z*-scores and encoding LD information through ${{\bf R}}$ and ${{\bf L}}$, HDL-C provides a high-dimensional, likelihood-based inference framework that scales efficiently to genome-wide analyses using only GWAS summary statistics and an LD reference.

In practical implementation, HDL-C is profiled over ${{h}_{12}}$ with fixed $( {\hat{h}_1^2,\hat{h}_2^2} )$, optimizing the log-likelihood via Newton–Raphson iteration with a convergence tolerance of ${{10}^{ - 6}}$.

### Proteins and their summary association statistics

This study focused on plasma proteins from the Pharma Proteomics Project, a precompetitive biopharmaceutical consortium that characterizes the plasma proteomic profiles of 54,219 UK Biobank participants. The proteome profiling was based on the Olink Proteomics proximity extension assay (PEA) for approximately 3,000 proteins, corresponding to the Olink Explore panel. For data processing, the first step involved downloading the pQTL summary statistics. This dataset provides comprehensive insights into the genetic determinants of protein levels. Subsequently, our attention was directed toward the genetic variants on the autosomes. Specifically, we retained all overlapping SNPs located on these chromosomes, ensuring a comprehensive coverage of autosomal genetic variations. The final step in our data preparation process entailed selecting genes positioned on the autosomes. For each of these genes, we identified and delineated the corresponding *cis* region, extending $\pm $1 Mb from the gene’s physical location. This approach enabled us to precisely target genomic regions that are likely to influence the expression levels of nearby genes, thereby providing a robust foundation for our subsequent analyses of the genetic architecture of protein expression.

### Summary association statistics of diseases

The UK Biobank GWAS summary statistics used in this report were obtained from the second wave of results released in 2018 by Neale’s group. We selected 200 ICD-10–coded diseases from the UK Biobank, each with over 1,000 recorded cases. These diseases span a broad spectrum of diagnostic categories, including malignant neoplasms (e.g., breast, colon, and lung cancer), cardiovascular conditions (e.g., angina pectoris, chronic ischemic heart disease, and atrial fibrillation), and a variety of musculoskeletal disorders (e.g., rheumatoid arthritis, spondylosis, and arthrosis). We also included common genitourinary diseases, endocrine disorders, and gastrointestinal conditions. In addition, we included several dermatological and respiratory diagnoses, as well as injuries and other frequent causes of hospital admission. By focusing on diseases with large case counts, we ensured adequate statistical power for subsequent analyses and captured a representative range of disease phenotypes in the UK Biobank cohort.

### Genome-wide pQTL analysis in males and females

UK Biobank genotyping and imputation (and quality control) were performed as described previously [36]. Individual protein levels (NPX) were inverse-rank normalized, including values below the limit of detection. Before the GWAS, each protein phenotype was adjusted for the following covariates, including age, age${{}^2}$, UK Biobank (UKB) center, UKB genetic array, the time between blood sampling and measurement, and the first 20 genetic principal components to account for population structure. Sex-stratified GWAS analyses were conducted separately in males and females using $REGSCAN$ [37]. Variants with minor allele frequency <0.05 were excluded.

### Simulation

To evaluate the performance of our methodology in detecting colocalization between *cis*-pQTL and disease traits, the simulations were conducted in 2 distinct settings: the first scenario involved 10% SNPs as causal, while the second scenario considered a single causal SNP. We randomly selected 300 *cis*-pQTL regions from the total of 2,826, ensuring that the distribution of *cis*-pQTL heritability ($h_1^2$)—computed from the top associated SNP in each region—closely matched the distribution observed across all regions. The SNP heritability of the top variant in each *cis*-region was calculated using the following formula:


\begin{eqnarray*}
{{h}^2} = \frac{{{{Z}^2}}}{{N + {{Z}^2}}},
\end{eqnarray*}

where *Z* is the GWAS *Z*-score (i.e., the estimated effect divided by its standard error), and *N* is the sample size. This value reflects the proportion of variance in protein abundance explained by the most strongly associated SNP per region.

In the first simulation scenario, we assumed a polygenic architecture with 10% of the SNPs in each region designated as causal. The heritability of the disease trait ($h_2^2$) was varied over the set $\{ {0.001,0.01,0.1} \}$, and the genetic correlation (${{r}_G}$) between the disease and *cis*-pQTL traits was drawn from $\{ {0,0.3,0.5,0.8,1} \}$. In the second scenario, we assumed a single causal variant model for the disease trait. While the *cis*-pQTL heritability remained as calculated from top variants, the heritability of the disease trait ($h_2^2$) was varied over $\{ {1 \times {{{10}}^{ - 5}},5 \times {{{10}}^{ - 4}},1 \times {{{10}}^{ - 4}},1 \times {{{10}}^{ - 3}}} \}$. For this setting, we evaluated genetic correlations ${{r}_G}$ from the set $\{ {0, - 1,1} \}$, representing scenarios of no correlation, perfect negative correlation, and perfect positive correlation between the protein and disease traits.

To simulate the genetic effects and phenotypic data, we followed a polygenic model. For each SNP *j* in the selected *cis*-pQTL region, the genetic effects ${{\beta }_{ij}}$ were drawn from a bivariate normal distribution. The distribution was specified as


\begin{eqnarray*}
\left( {\begin{array}{@{}*{1}{c}@{}} {{{\beta }_{1j}}}\\ {{{\beta }_{2j}}} \end{array}} \right) \sim \mathcal{N}\left( {\left( {\begin{array}{@{}*{1}{c}@{}} 0\\ 0 \end{array}} \right),\left( {\begin{array}{@{}*{2}{c}@{}} {h_1^2/m}&{{{r}_G}\sqrt {h_1^2h_2^2} /m}\\ {{{r}_G}\sqrt {h_1^2h_2^2} /m}&{h_2^2/m} \end{array}} \right)} \right),
\end{eqnarray*}

where $h_1^2$ and $h_2^2$ represent the heritability values for the *cis*-pQTL and disease traits, respectively, and *m* is the total number of causal SNPs selected in each simulation setting. The genetic effects ${{\beta }_{ij}}$ were then used to model the phenotypic data.

The phenotypic data for the 2 traits, ${{{{\bf y}}}_1}$ (*cis*-pQTL) and ${{{{\bf y}}}_2}$ (disease), were generated by applying the polygenic model:


\begin{eqnarray*}
{{{{\bf y}}}_i} = \mathop \sum \limits_{j = 1}^m {{{{\bf x}}}_{ij}}{{\beta }_{ij}} + {{\epsilon }_i},\left( {i = 1,2} \right)
\end{eqnarray*}

where ${{{{\bf x}}}_{ij}}$ represents the genotype data for SNP *j*, and ${{\epsilon }_i}$ denotes the residuals. These residuals were sampled from a multivariate normal distribution:


\begin{eqnarray*}
\left( {\begin{array}{@{}*{1}{l}@{}} {{{\epsilon }_1}}\\ {{{\epsilon }_2}} \end{array}} \right) \sim \mathcal{N}\left( {\left( {\begin{array}{@{}*{1}{l}@{}} 0\\ 0 \end{array}} \right),\left( {\begin{array}{@{}*{2}{c}@{}} {\left( {1 - h_1^2} \right){{\bf I}}}&0\\ 0&{\left( {1 - h_2^2} \right){{\bf I}}} \end{array}} \right)} \right),
\end{eqnarray*}

This distribution ensures that the total phenotypic variance for each trait sums to 1. The phenotypic data were generated for each simulation replicate under the specified heritability and genetic correlation settings. The estimation of genetic covariance and genetic correlation between the *cis*-pQTL trait and the disease trait was performed using the method described in the HDL-C study. HDL-C applied a likelihood-based framework to estimate these parameters. The LRT was used to assess the statistical significance of the genetic covariance, and the 95% confidence intervals for the genetic covariance were derived using the likelihood ratio approach, as detailed in the original method section. Each simulation setting was replicated 100 times to ensure robust performance.

### Colocalization analysis

We used the Bayesian colocalization analysis tool COLOC with the posterior probabilities testing the H4 colocalization hypothesis: testing for a single shared causal variant between the pair of traits. The tests were applied to the mapped *cis*-pQTL and the established GWAS summary statistics. SuSiE is a flexible model that estimates the posterior distribution of causal effects at each genomic locus, allowing for the identification of multiple causal variants within a single region. The analysis was performed on the mapped *cis*-pQTL regions and the corresponding GWAS summary statistics, with SuSiE estimating the posterior inclusion probabilities (PIPs) for each SNP in the region. These PIPs were used to assess the strength of evidence for each variant being causal, with the highest PIPs suggesting the most likely causal variants within the identified loci.

### Sensitivity and specificity

To evaluate the diagnostic performance of HDL-C and COLOC, receiver operating characteristic (ROC) curves were constructed. This involved plotting the true-positive rate (sensitivity) against the false-positive rate (1 − specificity) at various threshold settings. The AUC of these ROC curves was then calculated, providing a quantitative measure of the overall diagnostic accuracy of each method. A higher AUC value indicates superior diagnostic performance. To statistically compare the AUCs derived from the 2 methods, we employed the pROC package in R. This package provides a nonparametric approach to assess the significance of the difference between the AUCs.

### Drug target investigation

For the top 50 protein–disease pairs identified by HDL-C in male and female cohorts, we systematically investigated available drugs targeting these proteins using the DrugBank and Drugs.com databases. We aimed to identify therapeutic opportunities by classifying the drug–protein–disease combinations into 4 main categories: new, druggable, re-purposing, and validated. A protein–disease pair was considered validated if an existing drug was known to affect both the protein and the disease. Validated pairs were further classified into “Matched +” if the drug’s impact on the protein and disease was consistent with the effect direction observed in HDL-C analysis and “Matched −” if the effect direction differed from the HDL-C estimation. If a drug targeted the protein but was approved for treating a different disease than the one identified by HDL-C, it was classified as a re-purposing opportunity. This indicates potential to expand the drug’s use into new therapeutic areas. Protein–disease pairs were labeled as druggable if the protein is currently under clinical evaluation or considered viable for development into small-molecule therapies, regardless of existing drug approval. If no known drugs were available for a given protein–disease pair, it was classified as “New,” representing a novel therapeutic target for further exploration. This approach enabled the identification of potentially actionable therapeutic targets based on sex-specific HDL-C results.

## Source Code Availability and Requirements

- Project name: HDL-C
- Project homepage: https://github.com/zhenin/HDL/
- License: GPL-3.0 license
- SciCrunch RRID: SCR_027750
- System requirements
- Operating system: Linux or macOS
- Programming language: R version 3.6.0 or higher
- Package management: Dependencies include R packages dplyr, data.table, tidyverse. R package remotes for installation from GitHub.
- Hardware requirements: Standard computer; analyses in the study were performed on a single CPU core with a uniform memory allocation of 8 GB.

## Supplementary Material

giaf155_Supplemental_Files

giaf155_Authors_Response_To_Reviewer_Comments_original_submission

giaf155_GIGA-D-25-00203_original_submission

giaf155_GIGA-D-25-00203_Revision_1

giaf155_Reviewer_1_Report_original_submission Mingxuan Cai -- 6/18/2025

giaf155_Reviewer_1_Report_revision_1 Mingxuan Cai -- 12/8/2025

giaf155_Reviewer_2_Report_original_submission Jie Zheng -- 8/20/2025

giaf155_Reviewer_2_Report_revision_1 Jie Zheng -- 11/24/2025

## Data Availability

Individual-level genotype and phenotype data are available upon application to the UK Biobank. GWAS summary statistics were generated by the Neale laboratory [38]. Proteogenomic results and summary statistics from the UKB-PPP were accessed through the UKB-PPP portal [10]. The data and scripts used to generate Figs. 1–3, 4A, and Supplementary Figs. S1–S4 are available in the GitHub repository.

## Additional Files

**Supplementary Fig. S1**. Distribution of single–SNP *cis*–heritability estimates for plasma protein. The empirical distribution of the estimated heritability for the lead (top) variant in each *cis*–pQTL region. (a) Histogram of all 2,826 *cis*–pQTL regions. (b) Histogram of a random subset of 300 *cis*–pQTL regions drawn without replacement so that their heritability spectrum mirrors that of the full set. (c) Kernel–density overlays of the complete (blue) and subset (red) distributions. The *P* value was derived by a 2–sample Kolmogorov–Smirnov test, confirming that no systematic bias was introduced by down–sampling.

**Supplementary Fig. S2**. True-positive rate at fixed false-positive rate thresholds. TPR is summarized at FPR = 5% and 10% for different numbers of causal SNPs (10%, 1, 3, 5) and heritability levels (0.001, 0.01, 0.1).

**Supplementary Fig. 3**. Distributions of per-locus execution time of different methods. Boxplots of per-locus execution time (log_10_ seconds) across 50 simulation replicates for 300 *cis*-pQTL regions (i.e., $n = 15,000$ replicates per method). The central line marks the median, and the box spans the interquartile range (IQR); whiskers extend to 1.5$\times $ IQR.

**Supplementary Fig. S4**. Composition and enrichment across top-*N* thresholds. (a) Stacked proportions of category assignments—Druggable, Re-purposing, Matched$+ $, Matched$- $, and New—for the top-*N* HDL-C reported protein–disease pairs ($N\in\{ {10,20,30,40,50} \}$). Proportions are computed as *Count*/*Total*, where *Total* is the number of unique pairs at each *N*. (b) The fraction of Matched$+ $ pairs is shown with 95% confidence intervals, which provide binomially valid coverage for the underlying success proportion.

**Supplementary Table S1**. The AUC values of HDL-C, COLOC, SuSiE, and SharePro in simulation analyses. **Method**: Specifies the method used (HDL-C, COLOC, or SuSiE). **h11**: The simulated heritability level for the disease trait. **rg_threshold**: The threshold for the true genetic correlation used to define colocalization. **AUC**: The area under the receiver operating characteristic curve, reflecting the method’s ability to distinguish between colocalized and non-colocalized regions. **CausalSNPs**: Denotes the simulation scenario—either a single causal SNP or multiple causal SNPs per region.

**Supplementary Table S2**. Description of 200 ICD-10 coded diseases. This table provides detailed information for 200 disease phenotypes based on ICD-10 codes used in the UK Biobank analysis. **phenotype**: ICD-10 code corresponding to each disease. **description**: Full description of the disease. **variable_type**: Indicates whether the phenotype is categorical or numerical. **source**: Source of the phenotype definition (ICD-10). **n_non_missing**: Number of individuals with nonmissing phenotype data. **n_missing**: Number of individuals with missing phenotype data. **n_controls**: Number of individuals without the disease. **n_cases**: Number of individuals diagnosed with the disease.

**Supplementary Table S3**. Description of 2,826 proteins from the UK Biobank Pharma Proteomics Project. This table summarizes annotations and summary association statistics for 2,826 plasma proteins profiled in the UK Biobank Pharma Proteomics Project (UKB-PPP), used in *cis*-pQTL analyses. **UKBPPP_ProteinID**: Unique identifier of the protein, formatted as

$HGNC.\textit{symbol}\_UniProt\_OID\_Panel\_chr.\textit{Position}$ . **CHR, POS19, POS38**: Chromosome and position of the top associated SNP in GRCh37 (POS19) and GRCh38 (POS38) coordinates. **REF, ALT**: Reference and alternative alleles for the top SNP. **rsid**: dbSNP identifier of the top SNP. **BETA, SE**: Estimated effect size and standard error from *cis*-pQTL association testing. **ALTFREQ**: Frequency of the alternative allele. **N**: Sample size used in the association analysis. **Z**: *Z*-score, computed as the ratio of BETA to SE. **h^2^**: SNP heritability of the top variant in each *cis*-region.

**Supplementary Table S4**. Top 50 results of rediscovery analysis across methods and sex-specific UK Biobank populations. This Excel file contains the top 50 significant colocalization analysis results between 2,826 proteins and 200 ICD-10 coded disease traits, stratified by sex. Results were obtained using 4 methods: HDL-C, COLOC, SuSiE, and SharePro. Each method was applied separately to the male and female datasets from the UK Biobank. The **threshold** denotes the training-derived significance cutoff corresponding to the 50th-ranked signal, **measure_test** provides the value of the same ranking statistic in the opposite-sex test cohort, and **rediscovered** indicates whether the locus surpasses the training threshold in the test dataset. The **Method** and **Direction** columns specify the statistical framework used (HDL-C, HDL-L, Coloc, SuSiE, or SharePro) and the direction of replication.

**Supplementary Table S5**. Top 50 significant protein–disease associations from HDL-C analysis and cross-referencing with DrugBank. This table summarizes results from an extended HDL-C (0) analysis in which we investigated the top 50 significant protein–disease associations separately in male and female cohorts. The combined set included 92 unique protein–disease pairs. Each entry includes local genetic correlation estimates and inference statistics for HDL-C, along with DrugBank annotations for the corresponding protein targets. The file contains 4 sheets: matched, druggable, re-purposing, and new. **uniprot, protein**: UniProt accession and gene/protein name of the cis-pQTL. **phenotype, description.x**: ICD-10 code and disease description. **Heritability_1, Heritability_2**: Local SNP heritability estimates for the protein and disease traits, respectively. **Genetic_Covariance, Genetic_Correlation**: Local genetic covariance and correlation estimates from HDL-C. **Lower_bound_rg, Upper_bound_rg, P**: Likelihood-based confidence interval and *P* value for local genetic correlation. **Gender**: Indicates whether the result is from the male or female cohort. **drugbank_id, name, description.y**: DrugBank ID, compound name, and description of known compounds targeting the protein. **known_action, gene_name, cellular_location**: Drug–target interaction metadata and protein localization from DrugBank.

## Abbreviations

AUC: area under the curve; FPR: false-positive rate; GWAS: genome-wide association study; HDL: high-definition likelihood; HDL-C: high-definition likelihood for colocalization inference; ICD-10: International Classification of Diseases, 10th Revision; ITP: immune thrombocytopenic purpura; LD: linkage disequilibrium; LDL: low-density lipoprotein; Lp(a): apolipoprotein(a); LRT: likelihood ratio test; PC: principal component; PEA: proximity extension assay; PIP: posterior inclusion probability; pQTL: protein quantitative trait loci; QTL: quantitative trait loci; RDR: rediscovery rate; ROC: receiver operating characteristic; SNP: single-nucleotide polymorphism; SuSiE: Sum of Single Effects; TPR: true-positive rate.
