# Supplementary figures and images for: High-definition likelihood inference of genetic colocalization reveals protein biomarkers for human complex diseases
Source: Gigascience. 2026 Jan 23;15:giaf155. doi: 10.1093/gigascience/giaf155 (PMC12916012; doi:10.1093/gigascience/giaf155)

Full set (N = 2,826)    Simulation subset (n = 300)

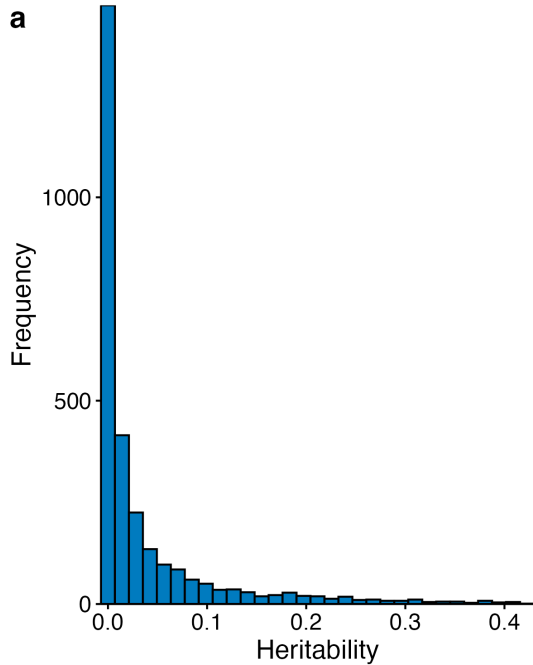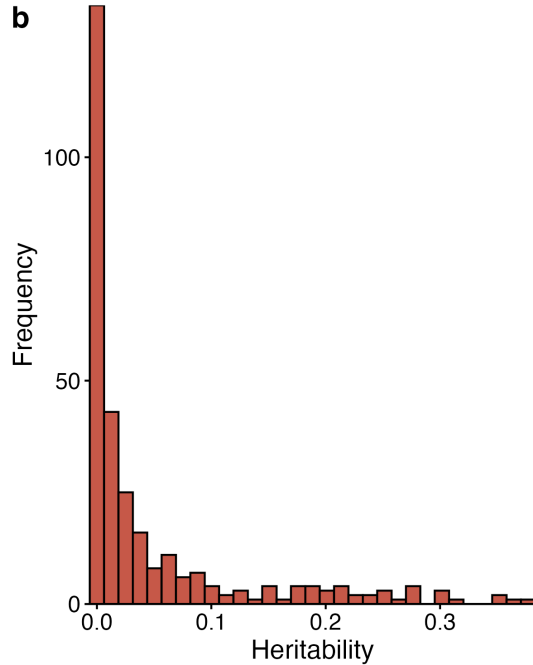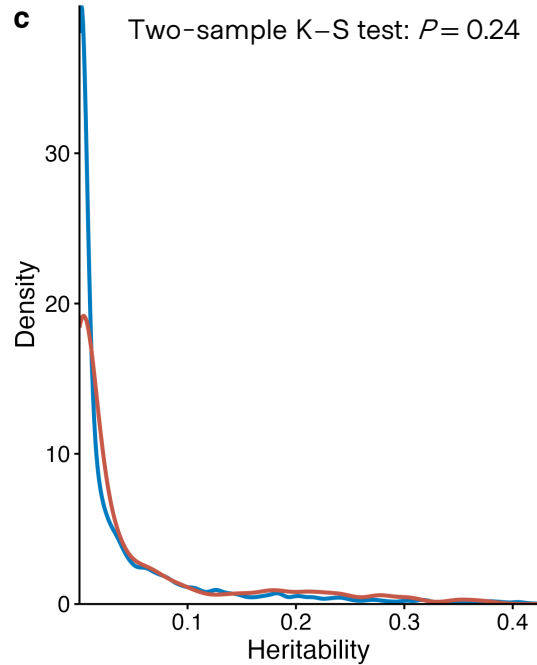

Supplement: giaf155_Supplemental_Files [file giaf155_supplemental_files.zip › SupFigure1.pdf]

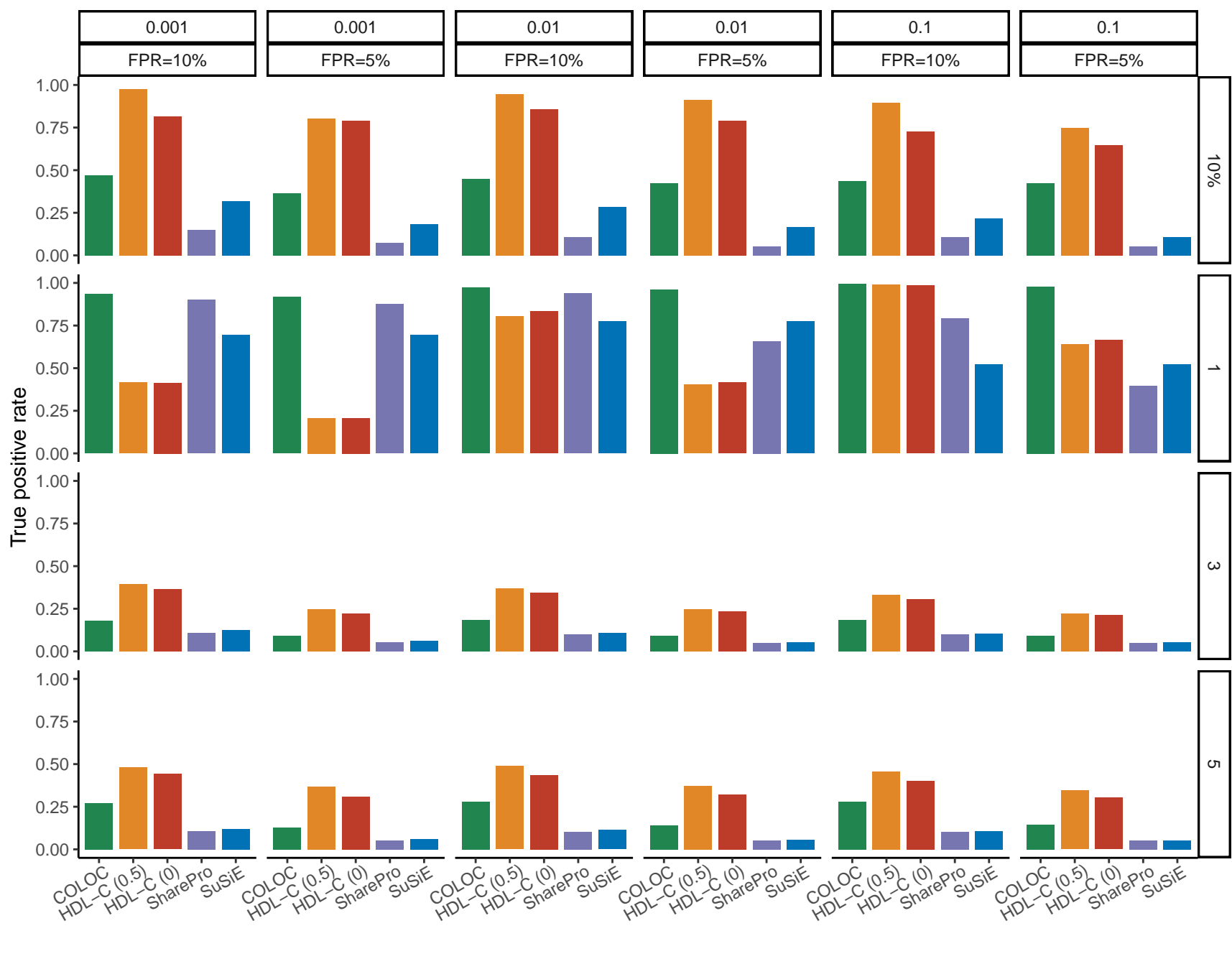

Supplement: giaf155_Supplemental_Files [file giaf155_supplemental_files.zip › SupFigure2.pdf]

Elapsed time (seconds, log scale)

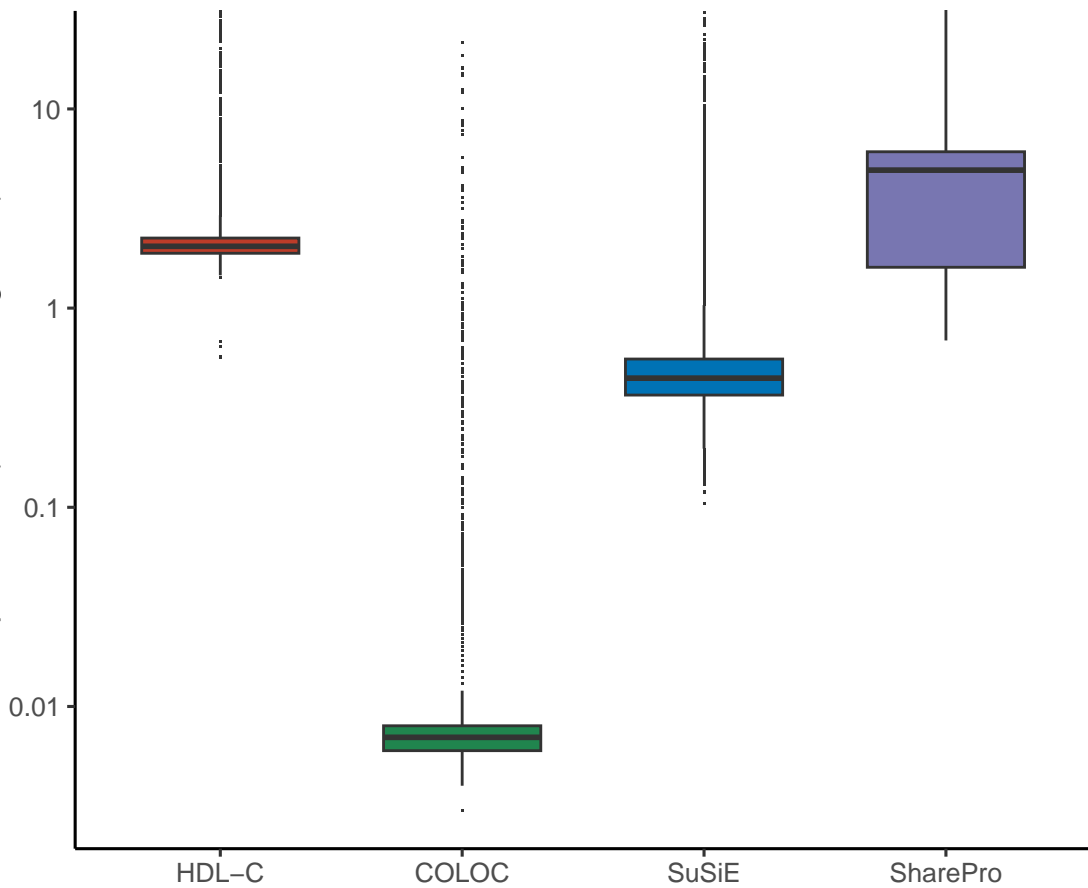

Supplement: giaf155_Supplemental_Files [file giaf155_supplemental_files.zip › SupFigure3.pdf]

**a**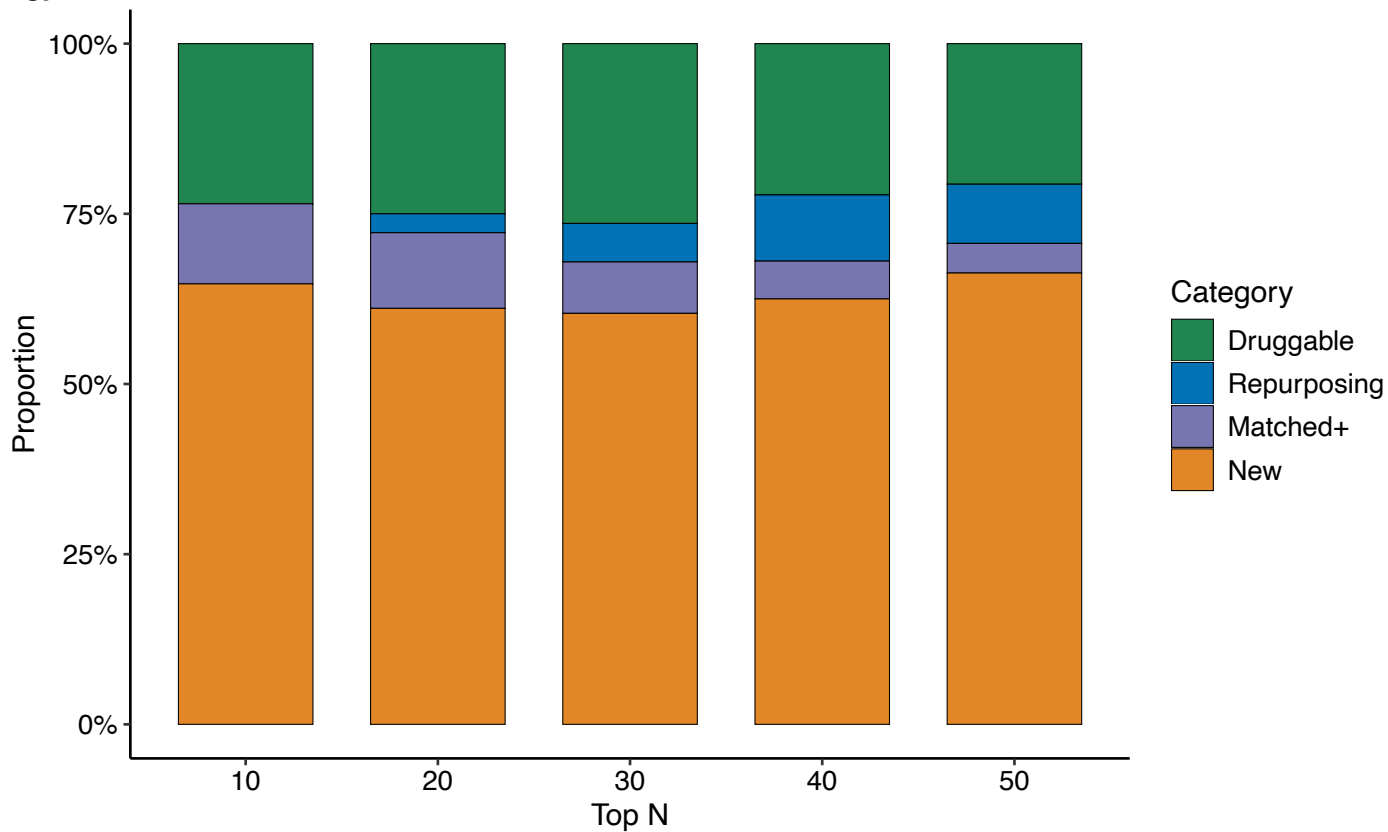**b**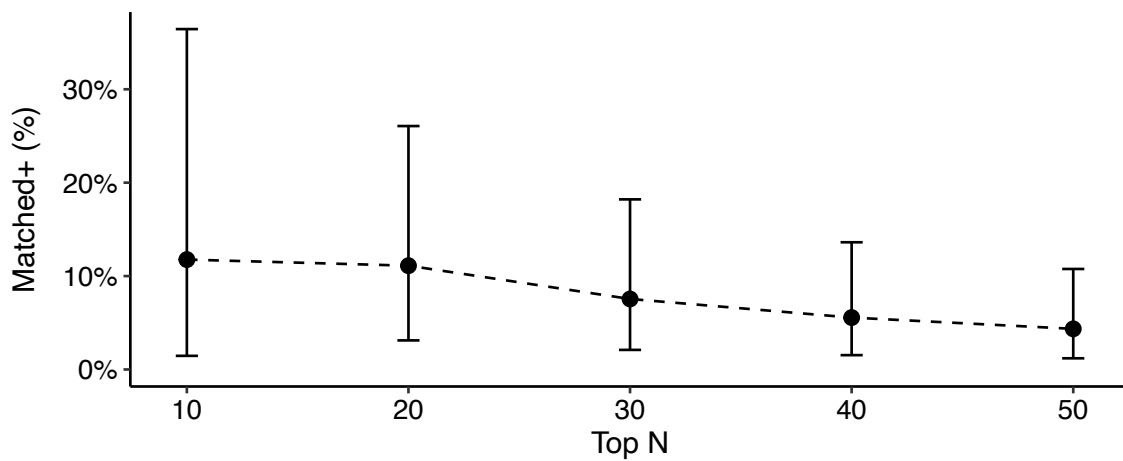

Supplement: giaf155_Supplemental_Files [file giaf155_supplemental_files.zip › SupFigure4.pdf]
