# Supplementary material for: High-definition likelihood inference of genetic colocalization reveals protein biomarkers for human complex diseases
Source: Gigascience. 2026 Jan 23;15:giaf155. doi: 10.1093/gigascience/giaf155 (PMC12916012; doi:10.1093/gigascience/giaf155)
Supplement: giaf155_GIGA-D-25-00203_original_submission [file giaf155_giga-d-25-00203_original_submission.pdf]

GigaScience

High-definition likelihood inference of colocalization reveals protein biomarkers for human complex diseases

--Manuscript Draft--

**Manuscript Number:**	GIGA-D-25-00203
**Full Title:**	High-definition likelihood inference of colocalization reveals protein biomarkers for human complex diseases
**Article Type:**	Technical Note
**Funding Information:**	National Natural Science Foundation of China (12171495) — Dr. Xia Shen<br>Key Technologies Research and Development Program (2022YFF1202105) — Dr. Xia Shen<br>Vetenskapsrådet (2022-01309) — Dr. Xia Shen
**Abstract:**	Background: Genetic colocalization analysis is essential for understanding the shared genetic basis between phenotypic traits. Such an analysis is particularly useful for discovering plasma proteins that have potential as therapeutic targets or clinical biomarkers. Improvement of existing tools is needed for better inference of potentially causal biomarkers.<br>Findings: We develop HDL-C, a high-definition likelihood inference method for genetic colocalization analysis. Based on simulations and observed rediscovery rates in real data analyses, we demonstrate that the HDL-C approach outperforms state-of-the-art methods, COLOC and SuSiE, in detecting genetic colocalization, thus enabling a more complete understanding of genetic connections at specific loci. Analyses of the top 50 protein–disease pairs identified by HDL-C in the male and female cohorts of the UK Biobank uncovered 40 previously validated drug-protein–disease combinations with approved drugs matching the phenotypes and 62 combinations with potential drug repurposing opportunities. Additionally, we identified 63 novel protein–disease pairs that suggest promising candidates for future therapeutic interventions.<br>Conclusion: This research establishes a robust framework for detecting colocalization signals, enabling the prioritization of disease-relevant protein targets and informing therapeutic development strategies.
**Corresponding Author:**	Xia Shen<br>Fudan University<br>Guangzhou, Please select CHINA
**Corresponding Author Secondary Information:**	
**Corresponding Author's Institution:**	Fudan University
**Corresponding Author's Secondary Institution:**	
**First Author:**	Yuying Li
**First Author Secondary Information:**	
**Order of Authors:**	Yuying Li<br>Ranran Zhai<br>Zhijian Yang<br>Ting Li<br>Yudi Pawitan<br>Xia Shen
**Order of Authors Secondary Information:**	

Powered by Editorial Manager® and ProduXion Manager® from Aries Systems Corporation

Additional Information:	
**Question**	**Response**
Are you submitting this manuscript to a special series or article collection?	No
**Experimental design and statistics**<br><br>Full details of the experimental design and statistical methods used should be given in the Methods section, as detailed in our Minimum Standards Reporting Checklist. Information essential to interpreting the data presented should be made available in the figure legends.<br><br>Have you included all the information requested in your manuscript?	Yes
**Resources**<br><br>A description of all resources used, including antibodies, cell lines, animals and software tools, with enough information to allow them to be uniquely identified, should be included in the Methods section. Authors are strongly encouraged to cite Research Resource Identifiers (RRIDs) for antibodies, model organisms and tools, where possible.<br><br>Have you included the information requested as detailed in our Minimum Standards Reporting Checklist?	Yes
**Availability of data and materials**<br><br>All datasets and code on which the conclusions of the paper rely must be either included in your submission or deposited in publicly available repositories (where available and ethically appropriate), referencing such data using a unique identifier in the references and in the “Availability of Data and Materials” section of your manuscript.	Yes

Powered by Editorial Manager® and ProduXion Manager® from Aries Systems Corporation

Have you have met the above requirement as detailed in our Minimum Standards Reporting Checklist?	
GigaScience has policies and guidelines in place for the use of generative AI-writing tools such as ChatGPT. If you have used such writing tools to assist with writing the manuscript this must be declared and cited in the text. Authors should not list AI-writing tools and other AI-assisted technologies as an author or co-author and should acknowledge that they are fully responsible for text generated or refined by AI-writing tools.<p><br><br>A summary of use (particularly in the introduction or among methods) needs to be included at the end of the paper, and the outputs should also be included as a supplementary file hosted in GigaDB or other open repositories. Please read our guidelines for more information. <p><br><br>By submitting to GigaScience, you are aware of the journal's AI-writing tools policy, and if you have declared use of such tools below, you have acknowledged this where appropriate in your manuscript and have made a summary of use and outputs available. <p><br><b>AI-assisted writing tools have been used in the preparation of this manuscript?	No

Powered by Editorial Manager® and ProduXion Manager® from Aries Systems Corporation

Manuscript Click here to access/download;Manuscript;FullText_250522.pdf

High-definition likelihood inference of colocalization reveals protein biomarkers for human complex diseases

Yuying Li[1,2,†], Ranran Zhai[2,3,†],Zhijian Yang[2,5], Ting Li[2,3], Yudi Pawitan[1], Xia Shen[1,2,3,4,*]

[1]Department of Medical Epidemiology and Biostatistics, Karolinska Institutet, Stockholm, Sweden

[2]Center for Intelligent Medicine Research, Greater Bay Area Institute of Precision Medicine (Guangzhou), Fudan University, Guangzhou, China

[3]State Key Laboratory of Genetic Engineering, Center for Evolutionary Biology, School of Life Sciences, Fudan University, Shanghai, China

[4]Centre for Global Health Research, Usher Institute, University of Edinburgh, Edinburgh, UK

[5]Institute for Molecular Medicine Finland (FIMM), HiLIFE, University of Helsinki, Helsinki, Finland

*Correspondence should be addressed to: shenxia911@gmail.com

†These authors contributed equally to this work.

Abstract

Background: Genetic colocalization analysis is essential for understanding the shared genetic basis between phenotypic traits. Such an analysis is particularly useful for discovering plasma proteins that have potential as therapeutic targets or clinical biomarkers. Improvement of existing tools is needed for better inference of potentially causal biomarkers.

Findings: We develop HDL-C, a high-definition likelihood inference method for genetic colocalization analysis. Based on simulations and observed rediscovery rates in real data analyses, we demonstrate that the HDL-C approach outperforms state-of-the-art methods, COLOC and SuSiE, in detecting genetic colocalization, thus enabling a more complete understanding of genetic connections at specific loci. Analyses of the top 50 protein–disease pairs identified by HDL-C in the male and female cohorts of the UK Biobank uncovered 40 previously validated drug-protein–disease combinations with approved drugs matching the phenotypes and 62 combinations with potential drug repurposing opportunities. Additionally, we identified 63 novel protein–disease pairs that suggest promising candidates for future therapeutic interventions.

Conclusion: This research establishes a robust framework for detecting colocalization signals, enabling the prioritization of disease-relevant protein targets and informing therapeutic development strategies.

Keywords

HDL-C, Colocalization, Genetic correlation, Proteomics, Complex diseases, Therapeutic targets

Introduction

Genetic influences underlying human diseases and traits remain an important area of investigation in genomics. Genome-wide association studies (GWAS) have significantly advanced genetic research by identifying numerous genomic regions linked to various traits and disease susceptibilities[1–5]. A key aspect of this exploration is understanding how variations in the genome correlate with variations in phenotypic traits, including those associated with complex diseases. This understanding not only reveals the genetic architecture of these traits, but also helps to identify potential therapeutic targets and biomarkers for disease prediction and management.

Plasma proteins, given their critical roles in various biological processes and disease pathways, serve as valuable biomarkers and therapeutic targets. The measured proteome encompasses pro-

teins secreted or shed in the blood circulation, playing the main roles in various molecular processes and mediating cross-tissue communications [6]. Their expression levels, often influenced by genetic variations, can provide insights into the molecular mechanisms of diseases. Recent technological advancements in high-throughput quantification of circulating proteins have led to large-scale studies of protein quantitative trait loci (pQTL) [7–13]. These studies have highlighted the potential of associating protein levels with DNA sequence variants that colocalize with risk alleles for common diseases. Such colocalizations can reveal disease-associated pathways, offering novel insights into drug targets and translational biomarkers.

Therefore, methods for better inference of genetic colocalization are essential in the joint analysis of molecular traits and complex diseases. COLOC is one of the most widely used methods for colocalization analysis, which aims to detect genetic colocalization between pairs of traits, such as the analysis of complex traits at specific molecular quantitative trait loci (QTL) [14]. This Bayesian model makes restrictive assumptions about the underlying shared genetic architecture at the given locus, e.g., one causal variant per trait. Such an assumption may not always hold in real-world datasets. While extensions of COLOC [15] using conditional regression have been attempted to address the issue of multiple variants, they rely on assumptions of independence among causal variants, which may not hold true, especially in the presence of extensive linkage disequilibrium (LD)[16,17]. The Sum of Single Effects (SuSiE) framework, integrated into the COLOC package, has addressed these limitations by enabling robust fine-mapping of multiple causal variants [18–20]. However, this approach primarily focuses on fine-mapping rather than quantifying colocalization itself.

As an alternative strategy, we propose the inference of *sufficiently high* estimated regional genetic correlations between two phenotypes at specific genomic loci to detect genetic colocalization. Unlike existing methods, this strategy quantifies colocalization through a single genetic correlation parameter without strict assumptions about the underlying genetic architecture. We previously developed the high-definition likelihood (HDL) method as a robust approach for estimating genetic correlations using GWAS summary statistics[21] and the recent local version of this method, HDL-L, to estimate local genetic correlations[22]. This advancement allows for a more granular exploration of genetic correlations at specific loci. Nevertheless, for the detection of colocalization, inference must be based on a conditional likelihood given a sufficiently high regional genetic correlation estimate.

In this study, we (i) develop the theory and implement high-definition likelihood for colocalization inference (HDL-C), (ii) demonstrate that HDL-C performs better than COLOC and SuSiE in detecting genetic colocalization, and (iii) apply the HDL-C method to investigate the colocalization between plasma proteins and complex diseases using data from the UK Biobank. Specifically,

we prioritize drug targets focusing on 2,826 plasma proteins and their colocalization with 200 diseases. This approach offers a new opportunity to explore the genetic basis of disease-protein associations, potentially uncovering novel insights into disease mechanisms.

Results

Overview of the HDL-C method

Developing regional genetic correlation in the context of causal variant sharing, we introduce a conditional likelihood-based framework. The HDL-C method extends HDL-L by optimizing the likelihood conditioning on a high point estimate of local genetic correlation and assesses the statistical evidence that the two traits not only exhibit correlated genetic effects but are likely influenced by shared causal variants.

Let the estimated genetic correlation in a given region be $\hat{r}_G = \hat{h}_{12}/\sqrt{\hat{h}_1^2\hat{h}_2^2}$, where $\hat{h}_1^2$, $\hat{h}_2^2$, $\hat{h}_{12}$, are the regional heritabilities for the two traits and their genetic covariance, with $|\hat{r}_G| > r_0$ for a pre-specified threshold $r_0 \in (0, 1)$, e.g., $r_0 = 0.5$. Under this strong correlation constraint, we test the hypothesis of colocalization by evaluating the significance of the estimated genetic covariance under the assumption that a strong genetic correlation implies a high probability of shared causal architecture. Let $\mathcal{L}(h_{12} \mid \mathbf{z}_1, \mathbf{z}_2, \hat{h}_1^2, \hat{h}_2^2)$ be the conditional likelihood. HDL-C constructs the conditional likelihood ratio test statistic under the constraint $(\hat{r}_G| > r_0)$ as:

$$T_{\text{HDL-C}} = -2\log\mathcal{R}(0 \mid |\hat{r}_G| > r_0) = -2\log\left[\frac{\mathcal{L}(h_{12} = 0 \mid \mathbf{z}_1, \mathbf{z}_2, \hat{h}_1^2, \hat{h}_2^2; |\hat{r}_G| > r_0)}{\mathcal{L}(\hat{h}_{12} \mid \mathbf{z}_1, \mathbf{z}_2, \hat{h}_1^2, \hat{h}_2^2; |\hat{r}_G| > r_0)}\right],$$

subject to the constraint $|\hat{r}_G| > r_0$, which ensures the region is likely to harbor aligned genetic effects. We define the HDL-C p-value as:

$$p_{\text{HDL-C}} = \begin{cases} P\left(W \geq T_{\text{HDL-C}}\right), & \text{if } |\hat{r}_G| > r_0, \\ 1, & \text{otherwise}, \end{cases}$$

where $W \sim \chi^2(1)$ follows a chi-squared distribution with 1 degree of freedom. This formulation ensures that only regions with sufficiently strong observed genetic correlation are subject to statistical testing for colocalization (see **Methods** for the likelihood reasoning of the procedure). For these regions, a significant $p_{\text{HDL-C}}$ indicates that the observed Z-score vectors $\mathbf{z}_1, \mathbf{z}_2$ are better explained by a model with non-zero h_{12} under strong genetic correlation, suggesting a high probability of shared causal variants driving the local genetic signal in both traits.

In contrast to standard colocalization methods, which typically model variant-level causal probabilities under strong prior assumptions, HDL-C exploits the summary-level multivariate Gaussian structure of Z-scores and the polygenic covariance encoding in the LD score matrix. HDL-C thus provides a high-dimensional, likelihood-based inference procedure for genetic colocalization, requiring only GWAS summary statistics and LD reference.

In practice, we recommend using $r_0 = 0.5$ or higher to balance power and specificity. The resulting HDL-C p-values can be reported alongside HDL-C estimates to identify regions where strong genetic correlation co-occurs with statistically significant covariance, thereby reinforcing causal inference.

To evaluate the performance of HDL-C, we conducted a series of simulation studies comparing it with COLOC and SuSiE. Given that COLOC inherently assumes a single causal single nucleotide polymorphism (SNP) per region, we designed simulations in two scenarios: (i) each region with a single causal SNP and (ii) each region with multiple causal SNPs, where we assumed that 10% of the SNPs were causal. In addition, for the purpose of analyzing protein molecules and complex diseases, we examined different levels of true regional heritability for the disease trait. For cis-pQTLs, we estimated the heritability of the top SNP in each cis-region (see **Methods**) and then randomly selected 300 cis-pQTLs reflecting the heritability distribution of the full set of 2,826 cis-pQTLs (Supplementary Fig. 1). This subset approach was used to manage computational efficiency, as conducting simulations in all pQTL regions would be excessively computationally intensive. In each simulation replicate, we generated phenotypic data for two traits and estimated their local genetic correlation. The true effect sizes of the causal variants were drawn from a bivariate normal distribution, given the true genetic correlation (see Methods). The summary association statistics were then calculated from a genome-wide association analysis by regressing the simulated phenotypic data against the corresponding genotypes at each SNP.

HDL-C outperforms COLOC and SuSiE in detecting genetic colocalization

Under multiple causal SNPs assumption, across varying thresholds for the absolute true genetic correlation values to define true genetic colocalization (0 to 1) and true regional heritability levels, HDL-C outperformed COLOC and SuSiE (**Fig. 1a**). The Area Under the Curve (AUC) of HDL-C ranged from 0.92 to 0.98, compared to 0.69 to 0.93 for COLOC and 0.68 to 0.76 for SuSiE. Under one causal SNP assumption, since the true local genetic correlation would be 1 or -1 if there was colocalization, we compared the performance under different true regional heritability values (**Fig. 1b**). When the regional heritability was relatively small, i.e., 1×10^{-5}, SuSiE performed the best. As the heritability increased to 0.001, these three methods achieved similar performance, with AUC values of 0.99 for

SuSiE, 0.98 for HDL-C, and 0.97 for COLOC.

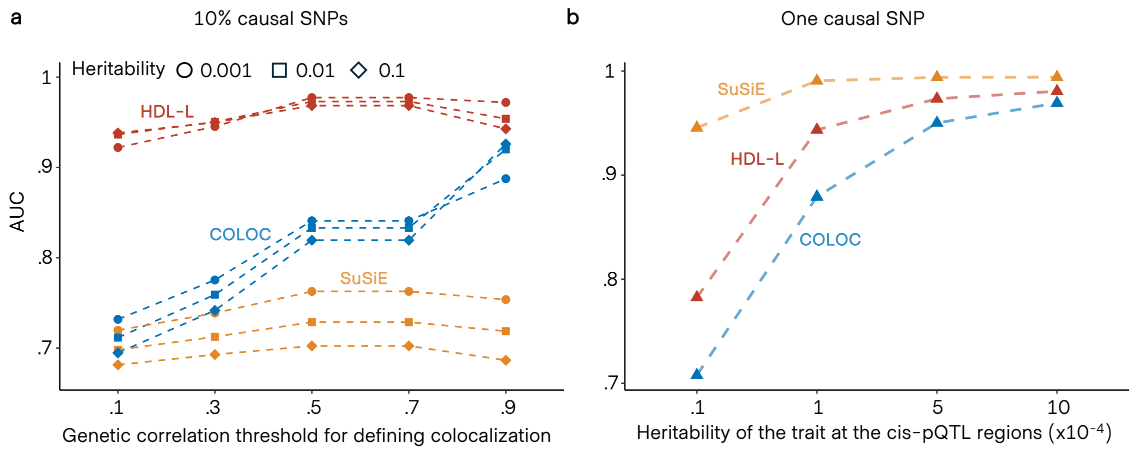

Figure 1: Performance Comparison of HDL-C, COLOC, and SuSiE in detecting genetic colocalization by AUC. This figure illustrates the performance of HDL-C, COLOC, and SuSiE in the detection of genetic colocalization under simulation. The colocalization level was simulated across different true genetic correlation values. (a) In the multiple causal variant scenario, where 10% of the SNPs were designated as causal, the x-axis represents the absolute true genetic correlation values used to define genetic colocalization. We examined three different levels of true regional heritability for disease traits corresponding to cis-pQTL regions. (b) In the single causal variant scenario, we randomly selected one causal SNP within each cis-pQTL region and simulated four distinct levels of true heritability for the disease traits, confined to the same genomic region.

HDL-C has higher rediscovery rates in two independent samples

To demonstrate our theory in real data analyses, we evaluated their abilities to detect genetic colocalization between 200 ICD-10 coded diseases and 2,826 proteins in the UK Biobank. We focused on cis-pQTL regions to explore the shared genetic architecture between these diseases and proteins in male and female populations. We used two validation settings. In the first setting, we used female data for training and male data for testing. In the second setting, we used male data for training and female data for testing. This design allowed us to evaluate the reproducibility of findings across sex-stratified cohorts directly. We provide detailed descriptions of the diseases and their associated proteins in Supplementary Tables 2-3. The top 50 significant colocalization results from HDL-C, COLOC, and SuSiE analyses were selected from the training set (Supplementary Table 4), and we examined the rediscovery rates (RDR) for these methods in the test set as the proportion of overlapping results between the top 50 findings in the test and training sets (**Fig. 2**).

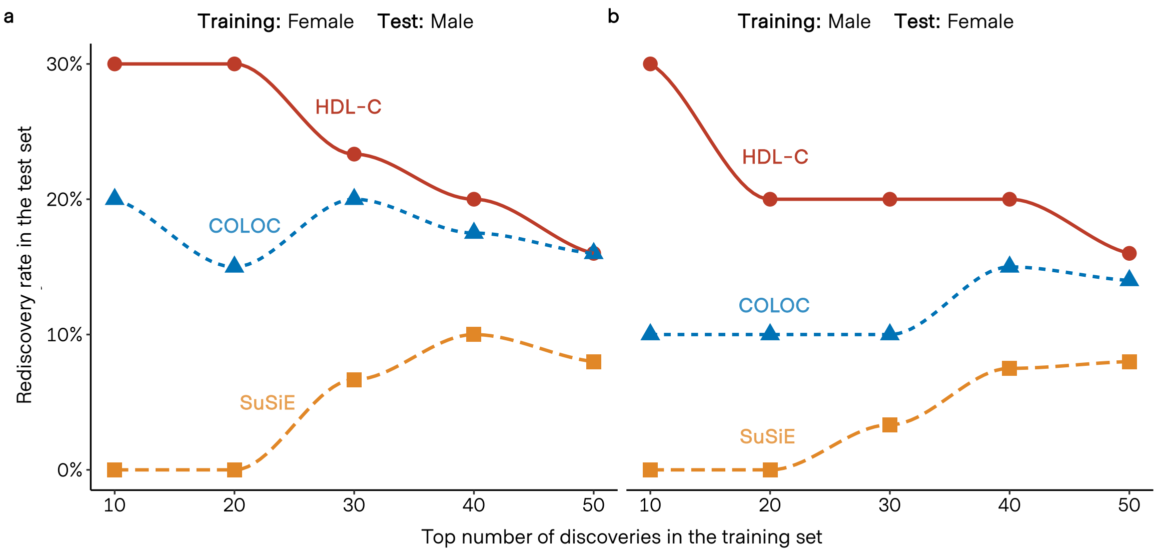

Figure 2: Rediscovery rates of HDL-C, COLOC, and SuSiE under two validation settings. (a) Training was performed on the female dataset and testing on the male dataset. (b) Training was performed on the male dataset and testing on the female dataset. The x-axis shows the top N significant results selected in the training set. The y-axis shows the proportion of these signals rediscovered in the test set. We applied HDL-C, COLOC, and SuSiE on ICD-10 coded 200 diseases and 2,826 proteins summary association statistics in the UK Biobank male and female population.

HDL-C achieved higher RDR than COLOC and SuSiE in both cross-validation scenarios. This result suggests that HDL-C can robustly detect shared genetic signals and adapt to sex-specific genetic variation. Some diseases may have sex-specific patterns: This would influence the RDR[23,24], but not affect the comparison of performance between the methods. Overall, HDL-C showed better performance under these conditions. COLOC and SuSiE could identify overlapping genetic loci but often yielded lower RDR compared to HDL-C. This finding showed the advantage of HDL-C's likelihood-based framework in detecting robust colocalization when applied to real data.

HDL-C prioritizes drug targets for human complex diseases

We extended our analysis by investigating the top 50 genetically correlated protein–disease pairs identified by HDL-C in the male and female subcohorts, respectively, resulting in 92 unique protein–disease combinations. Each of these combinations was cross-referenced with DrugBank (Supplementary Table 5). Integrating HDL-C discoveries with existing drug information, there were 40 validated drug-protein-disease combinations where a given drug targets the same protein and treats

the same disease or causes the same side effect ("Matched"). For all of the 40 matched combinations, the HDL-C inferred protein's causal effect directions were consistent with the corresponding drug action direction ("Matched $^{+}$") (**Fig. 3a**). We also identified 62 combinations where the drugs have different approved indications that differ from the diseases or side effects identified in the HDL-C results ("Re-purposing"), suggesting potential re-purposing opportunities. Furthermore, we discovered 63 protein–disease pairs where the proteins are not targeted by any drug in DrugBank ("New"), indicating potential novel therapeutic targets if the potential causal effects can be validated. In addition, we denoted 133 combinations as "Druggable", which means their ongoing evaluation in clinical trials or their viability for development into small-molecule therapies. We further showed the distribution of these drug-protein–phenotype combinations per protein (**Fig. 3b**).

For example, we observed that Tyrosine-protein kinase Fes/Fps (FES, UniProt P07332) exhibited a protective effect against chronic ischemic heart disease (ICD10: I25), as indicated by a significant local genetic correlation estimate of −0.99 (95% CI, −0.54 to −1.00) in females and −0.81 (95% CI, −0.49 to −1.00) in males (**Fig. 3c**). Fostamatinib - marketed as Tavalisse since its FDA approval on April 17, 2018 - was developed as a spleen tyrosine kinase (SYK) inhibitor for rheumatoid arthritis and immune thrombocytopenic purpura (ITP). However, studies have demonstrated that its active metabolite (R406) can also inhibit FES, a kinase implicated in protective inflammatory regulation[25]. While fostamatinib's anti-inflammatory properties have been investigated for mitigating vascular damage (and even acute respiratory distress syndrome in severe COVID-19), recent clinical evidence points to an increased incidence of cardiovascular side effects, notably hypertension, that may exacerbate ischemic heart conditions. This aligns with our findings of genetic correlation.

Aminocaproic acid is an antifibrinolytic agent that, by inhibiting plasminogen activation, may potentiate the prothrombotic environment in individuals with elevated apolipoprotein(a) [Lp(a)]. Higher Lp(a) levels themselves are well-documented risk factors for atherosclerotic disease, including angina pectoris, due to Lp(a)'s structural similarity to plasminogen and resultant interference with normal fibrinolysis. Thus, when aminocaproic acid further restricts fibrinolysis, it can intensify the cardiovascular risk posed by elevated Lp(a), leading to an increased incidence or severity of angina pectoris. This mechanistic interplay aligns with our significant local genetic correlation finding (0.94 with 95% CI (0.70,1.00) between Lp(a) and Angina pectoris (**Fig. 3c**), underscoring the shared risk pathway involving fibrinolysis inhibition and Lp(a)-related atherogenesis.

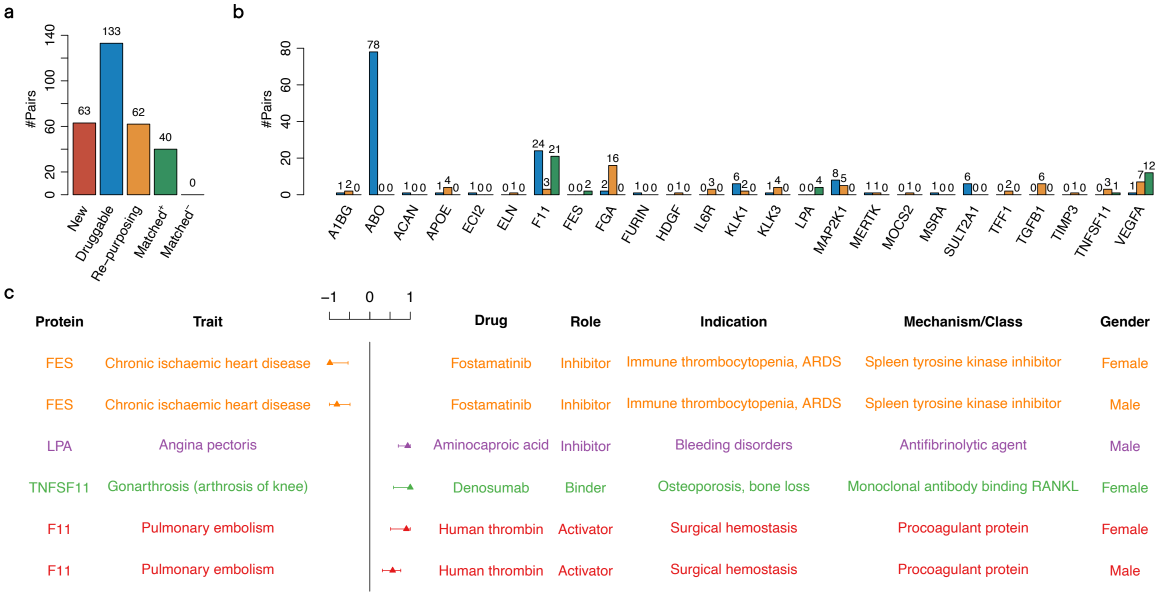

Figure 3: Drug targets inferred by local genetic correlation analysis. (a) Drug-protein–phenotype combinations identified from the top 50 HDL-C results in separate male and female cohorts, grouped into four categories: ”New”, representing novel protein–disease associations with no existing drugs; ”Druggable”, where proteins are under clinical evaluation or considered viable for small-molecule development; ”Repurposing”, where existing drugs are approved for different diseases; and “Validated”, where known drugs affect both the protein and the disease. Validated combinations are further subdivided into ”Matched +”, where the drug's effect direction aligns with the HDL-C estimation, and ”Matched −”, where the effect direction differs from the HDL-C findings. (b) Number of distinct categories per protein. (c) Representative examples of validated known targets, including drug descriptions, their primary indications or side effects, and HDL-C effect estimates. The local genetic correlation estimates are shown as solid circles with 95% confidence intervals (whiskers).

TNF superfamily member 11 (TNFSF11) displayed a risk-increasing effect on gonarthrosis (arthrosis of the knee) disease (ICD10: M17), with a significant local genetic correlation estimation of 1.00 and 95% CI (0.59, 1.00) (**Fig. 3c**). Gonarthrosis is marked by both progressive cartilage breakdown and pathologic remodeling of the subchondral bone. Increasing evidence showed that the RANK–RANKL–OPG axis is a key mediator in this process, with elevated RANKL driving osteoclast activity and contributing to aberrant bone turnover in Osteoarthritis [26,27]. Experimental studies using in vitro and animal models suggest that inhibiting RANKL can reduce excessive osteoclast-mediated resorption in the subchondral bone, potentially slowing disease progression [28,29]. Denosumab, a human monoclonal antibody targeting RANKL, effectively suppresses osteoclast formation and bone resorption and is currently approved for osteoporosis and skeletal metastases [30]. Although its use in arthrosis of the knee remains a large-scale clinical trial to verify, these findings illustrate

a plausible rationale for exploring RANKL inhibition as part of a disease-modifying strategy in osteoarthritis management. In summary, these findings demonstrate how integrating genetic correlation signals with drug databases can pinpoint both established and emergent therapeutic opportunities, particularly for complex disorders.

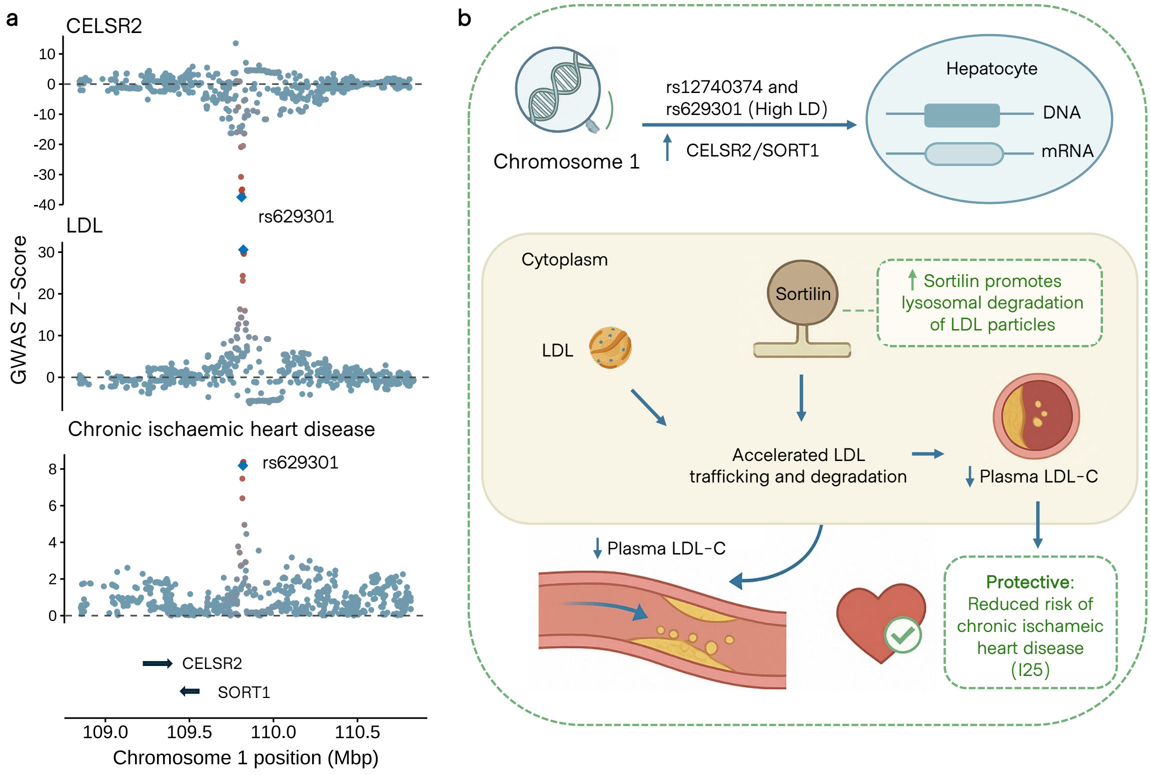

Figure 4: Potential new therapeutic target for chronic ischemic heart disease. (a) Regional association plots around CELSR2 showing: The cis-pQTL signal for plasma CELSR2 protein (top), the GWAS signal for LDL (medium), and the GWAS signal for chronic ischemic heart disease (bottom). The blue diamond shows the sentinel pQTL variant. Other variants are colored by LD to the sentinel pQTL. (b) Schematic illustration depicting the role of rs629301 in modulating CELSR2 expression and its downstream impact on LDL-C levels and ischemic heart disease (I25) risk.

Among the 63 newly identified protein–disease pairs, for instance, the HDL-C analysis found that the cadherin EGF LAG seven-pass G-type receptor 2 (CELSR2) was a potential target for chronic ischemic heart disease (ICD-10: I25). In both sexes, the local genetic correlation was nearly -1 (−0.94 in males, 95 % CI: −0.74 to −1.00; and −1.00 in females, 95 % CI: −0.62 to −1.00), driven by rs629301, a variant tightly linked to the well-studied rs12740374. The lead variant rs629301 was associated with CELSR2 protein levels in plasma, indicating a regulatory effect. This modulation likely influenced LDL-cholesterol levels and contributed to ischemic heart disease risk (**Fig. 4a**). Existing studies

indicate that rs12740374 modulates the hepatic expression of CELSR2 and its neighboring gene Sortilin 1 (SORT1), affecting LDL-cholesterol levels and coronary event risk. Functional analyses in human hepatocytes and mouse models have shown that elevated sortilin accelerates intracellular trafficking and lysosomal degradation of APOB-containing lipoproteins, reducing atherogenic particle pools [31,32] (**Fig. 4b**). These findings suggest CELSR2 as a potential therapeutic target for lipid-lowering therapies in chronic ischemic heart disease.

Discussion

We introduced HDL-C, a new and natural approach to infer colocalization based on sufficiently high genetic correlation. The analyses indicate that HDL-C outperforms both COLOC and SuSiE in detecting genetic colocalization in simulated and real datasets. In the top 50 findings for male and female UK Biobank subcohorts, HDL-C not only demonstrated robust efficacy but also identified previously unrecognized genetic associations between plasma proteins and diseases. The identification of these colocalized protein-disease pairs helped understand the genetic basis of complex diseases. The results prioritized novel protein targets for further investigation, which might lead to the development of new therapeutic strategies and clinical biomarkers.

From a methodological perspective, COLOC employs summary statistics with prior probabilities to infer whether two traits share a causal variant. This method distinguishes among multiple hypotheses in a Bayesian framework, notably H3 (indicating distinct causal variants for the two traits) and H4 (indicating a shared causal variant for both traits). Although COLOC's reliance on prior probabilities enhances flexibility, it also introduces potential bias if default priors are arbitrarily selected and not validated by sensitivity analyses. Furthermore, COLOC does not model multiple causal variants simultaneously, limiting its utility in regions of high LD, where distinguishing competing hypotheses (H3 vs H4) becomes challenging. Thus, careful application and sensitivity analyses are essential to ensure robust conclusions.

SuSiE enhances colocalization inference by explicitly modeling multiple causal signals, improving accuracy over single-variant methods. However, its performance depends critically on high-quality LD estimates. Mismatches between the LD reference panel and the study population can lead to spurious signals. Additionally, selecting the parameter L (the number of allowed causal effects) requires consideration: underestimating L risks missing true signals, while overestimating L may fragment true signals or overfit noise. In our simulations, the default L setting was adopted.

HDL-C addresses these limitations by detecting sufficiently high local genetic correlation using GWAS summary statistics while accounting for LD. This approach captures associations between

genetic effect vectors across traits, independent of the number of causal variants. For example, a shared causal variant with pleiotropic effects induces proportional effect estimates across SNPs in LD, producing a strong local genetic correlation (approaching ± 1.0 depending on the directionality of the effect). Conversely, unshared distinct causal variants produce low correlations, reflecting independent signals. Another intuitive advantage is that it inherently accounts for the direction and magnitude of effects, not just their existence.

This work presents a novel perspective on colocalization analysis, especially offering a better understanding of the genetic colocalization between human plasma proteins and complex traits. The insights gained from this study are not only valuable for genetics research but also have broad implications for the fields of personalized medicine and drug development.

Methods

Theory of the HDL-C method The high-definition likelihood inference of genetic colocalization (HDL-C) is conceptually grounded in the likelihood function of local genetic covariance, derived from GWAS summary statistics under a bivariate Gaussian model. For two traits with local z-score vectors $\mathbf{z}_1$ and $\mathbf{z}_2$, we model their joint distribution as:

$$\begin{bmatrix} \mathbf{z}_1 \\ \mathbf{z}_2 \end{bmatrix} \sim \mathcal{N}\left(0, \begin{bmatrix} \Sigma_{11} & \Sigma_{12} \\ \Sigma_{12}^{\top} & \Sigma_{22} \end{bmatrix}\right),$$

where $\Sigma_{ii} = \frac{N_i h_i^2}{M}\mathbf{L} + \mathbf{R}$ and $\Sigma_{12} = \frac{\sqrt{N_1 N_2} h_{12}}{M}\mathbf{L}$, with h_{12} denoting the local genetic covariance, and $\mathbf{L} = \mathbf{R}^2$ and $\mathbf{R}$ the LD score and LD correlation matrices, respectively. Let $\mathcal{L}(h_{12} \mid \mathbf{z}, \hat{h}_1^2, \hat{h}_2^2)$ denote the conditional likelihood of the genetic covariance. The theoretical definition of HDL-C interprets *colocalization evidence* as the normalized likelihood mass above a user-defined genetic correlation threshold r_0, that is:

$$\text{HDL-C score} = \int_{h_{12} > r_0\sqrt{\hat{h}_1^2 \hat{h}_2^2}} \frac{\mathcal{L}(h_{12} \mid \mathbf{z}, \hat{h}_1^2, \hat{h}_2^2)}{\int_{-\infty}^{\infty} \mathcal{L}(h_{12} \mid \mathbf{z}, \hat{h}_1^2, \hat{h}_2^2)\, \mathrm{d}h_{12}}\, \mathrm{d}h_{12}.$$

This quantity represents the posterior weight (under uniform prior) assigned to the hypothesis that the two traits share a sufficiently large positive genetic correlation, and thus likely a shared causal signal.

However, in practice, especially when the likelihood function is sharply peaked or the genetic correlation estimate $\hat{r}_G$ is large, this area-based proportion often approaches 1, providing little in-

ferential resolution, particularly under multiple testing across genomic regions. Therefore, we propose a practical inference procedure based on the *conditional likelihood ratio test*, under the constraint $|\hat{r}_G| > r_0$. Specifically, we test:

$$H_0 : h_{12} = 0 \quad \text{vs.} \quad H_1 : h_{12} \neq 0, \quad \text{conditional on } |\hat{r}_G| > r_0.$$

This yields the HDL-C p-value:

$$p_{\text{HDL-C}} = \begin{cases} P\left(W \geq -2\log \frac{\mathcal{L}(0)}{\mathcal{L}(\hat{h}_{12})}\right), & \text{if } |\hat{r}_G| > r_0 \\ 1, & \text{otherwise,} \end{cases}$$

where $W \sim \chi^2(1)$ follows a chi-squared distribution with 1 degree of freedom, and the likelihoods are evaluated conditionally on the estimated $\hat{h}_1^2$ and $\hat{h}_2^2$, as justified in Ning et al. [21].

This conditional framework maintains the theoretical grounding in likelihood theory while providing statistically discriminative p-values suitable for genome-wide inference, with robustness to sharp likelihood peaks and multiplicity effects. It complements and extends existing colocalization methods by incorporating both effect size magnitude and likelihood-based uncertainty.

Proteins and their summary association statistics This study focused on plasma proteins from the Pharma Proteomics Project, which is a precompetitive biopharmaceutical consortium characterizing the plasma proteomic profiles of 54,219 UK Biobank participants. The proteome profiling was based on the Olink©Proteomics' proximity extension assay (PEA) for about 3,000 proteins, corresponding to the Olink©Explore panel. For data processing, the first step involved downloading the protein quantitative trait loci (pQTL) summary statistics. This dataset provides comprehensive insights into the genetic determinants of protein levels. Subsequently, our attention was directed towards the genetic variants on the autosomes. Specifically, we retained all overlapping SNPs located on these chromosomes, ensuring a comprehensive coverage of autosomal genetic variations. The final step in our data preparation process entailed selecting genes positioned on the autosomes. For each of these genes, we identified and delineated the corresponding cis Region, extending ± 1 Mb from the gene's physical location. This approach allowed us to precisely target genomic regions that are likely to influence the expression levels of the nearby genes, thereby providing a robust foundation for our subsequent analyses in understanding the genetic architecture of protein expression.

Summary association statistics of diseases The UK Biobank GWAS summary statistics used in this report were obtained from the second wave of results released in 2018 by Neale's group. We selected 200 ICD-10-coded diseases from the UK Biobank, each with over 1,000 recorded cases. These diseases span a broad spectrum of diagnostic categories, including malignant neoplasms (for example, breast, colon, and lung cancer), cardiovascular conditions (such as angina pectoris, chronic ischemic heart disease, and atrial fibrillation), and a variety of musculoskeletal disorders (for instance, rheumatoid arthritis, spondylosis, and arthrosis). We also included common genitourinary diseases, endocrine disorders, and gastrointestinal conditions. In addition, we included several dermatological and respiratory diagnoses, as well as injuries and other frequent causes of hospital admission. By focusing on diseases with large case counts, we ensured adequate statistical power for subsequent analyses and captured a representative range of disease phenotypes in the UK Biobank cohort.

Genome-wide pQTL analysis in males and females UK Biobank genotyping and imputation (and quality control) were performed as described previously[33]. Individual protein levels (NPX) were inverse-rank normalized, including values below the limit of detection (LOD). Before the genome-wide association study (GWAS), each protein phenotype was adjusted for the following covariates, including age, age^2, UK Biobank center, UKB genetic array, the time between blood sampling and measurement, and the first 20 genetic principal components to account for population structure. Sex-stratified GWAS analyses were conducted separately in males and females using *REGSCAN*[34]. Variants with minor allele frequency < 0.05 were excluded.

Simulation To evaluate the performance of our methodology in detecting colocalization between cis-pQTLs and disease traits. The simulations were carried out in two distinct settings: the first scenario involved 10% SNPs as causal, while the second scenario considered a single causal SNP. We randomly selected 300 cis-pQTL regions from the total of 2,826, ensuring that the distribution of cis-pQTL heritability (h_1^2)—computed from the top associated SNP in each region—closely matched the distribution observed across all regions. The SNP heritability of the top variant in each cis-region was calculated using the formula:

$$h^2 = \frac{Z^2}{N + Z^2},$$

where Z is the GWAS Z-score (i.e., the estimated effect divided by its standard error) and N is the sample size. This value reflects the proportion of variance in protein abundance explained by the most strongly associated SNP per region.

In the first simulation scenario, we assumed a polygenic architecture with 10% of the SNPs in each region designated as causal. The heritability of the disease trait (h_2^2) was varied over the set $\{0.001, 0.01, 0.1\}$, and the genetic correlation (r_G) between the disease and cis-pQTL traits was drawn from $\{0, 0.3, 0.5, 0.8, 1\}$. In the second scenario, we assumed a single causal variant model for the disease trait. While the cis-pQTL heritability remained as calculated from top variants, the heritability of the disease trait (h_2^2) was varied over $\{1 \times 10^{-5}, 5 \times 10^{-4}, 1 \times 10^{-4}, 1 \times 10^{-3}\}$. For this setting, we evaluated genetic correlations r_G from the set $\{0, -1, 1\}$, representing scenarios of no correlation, perfect negative correlation, and perfect positive correlation between the protein and disease traits.

To simulate the genetic effects and phenotypic data, we followed a polygenic model. For each SNP j in the selected cis-pQTL region, the genetic effects β_{ij} were drawn from a bivariate normal distribution. The distribution was specified as:

$$\begin{pmatrix} \beta_{1j} \\ \beta_{2j} \end{pmatrix} \sim \mathcal{N}\left(\begin{pmatrix} 0 \\ 0 \end{pmatrix}, \begin{pmatrix} h_1^2/m & r_G\sqrt{h_1^2 h_2^2}/m \\ r_G\sqrt{h_1^2 h_2^2}/m & h_2^2/m \end{pmatrix} \right), \tag{1}$$

where h_1^2 and h_2^2 represent the heritability values for the cis-pQTL and disease traits, respectively, and m is the total number of causal SNPs selected in each simulation setting. The genetic effects β_{ij} were then used to model the phenotypic data.

The phenotypic data for the two traits, $\mathbf{y}_1$ (cis-pQTL) and $\mathbf{y}_2$ (disease), were generated by applying the polygenic model:

$$\mathbf{y}_i = \sum_{j=1}^{m} \mathbf{x}_{ij}\beta_{ij} + \varepsilon_i, (i = 1, 2) \tag{2}$$

where $\mathbf{x}_{ij}$ represents the genotype data for SNP j and ε_i denotes the residuals. These residuals were sampled from a multivariate normal distribution:

$$\begin{pmatrix} \varepsilon_1 \\ \varepsilon_2 \end{pmatrix} \sim \mathcal{N}\left(\begin{pmatrix} 0 \\ 0 \end{pmatrix}, \begin{pmatrix} (1-h_1^2)\mathbf{I} & 0 \\ 0 & (1-h_2^2)\mathbf{I} \end{pmatrix} \right), \tag{3}$$

This distribution ensures that the total phenotypic variance for each trait sums to 1. The phenotypic data were generated for each simulation replicate under the specified heritability and genetic correlation settings. The estimation of genetic covariance and genetic correlation between the cis-pQTL trait and the disease trait was performed using the method described in the HDL-C paper . HDL-C applied a likelihood-based framework to estimate these parameters. The likelihood ratio test (LRT) was used to assess the statistical significance of the genetic covariance, and the 95%

confidence intervals for the genetic covariance were derived using the likelihood ratio approach, as detailed in the original method section. Each simulation setting was replicated 100 times to ensure robust performance.

Colocalization analysis We used the Bayesian colocalization analysis tool COLOC with the posterior probabilities testing the H4 colocalization hypothesis: testing for a single shared causal variant between the pair of traits. The tests were applied to the mapped cis-pQTL and the established GWAS summary statistics. SuSiE is a flexible model that estimates the posterior distribution of causal effects at each genomic locus, allowing for the identification of multiple causal variants within a single region. The analysis was performed on the mapped cis-pQTL regions and the corresponding GWAS summary statistics, with SuSiE estimating the posterior inclusion probabilities (PIPs) for each SNP in the region. These PIPs were used to assess the strength of evidence for each variant being causal, with the highest PIPs suggesting the most likely causal variants within the identified loci.

Area under receiver operating characteristic curve To evaluate the diagnostic performance of HDL-C and COLOC, Receiver Operating Characteristic (ROC) curves were constructed. This involved plotting the true positive rate (sensitivity) against the false positive rate (1-specificity) at various threshold settings. The Area Under the Curve (AUC) of these ROC curves was then calculated, providing a quantitative measure of the overall diagnostic accuracy of each method. A higher AUC value indicates superior diagnostic performance. To statistically compare the AUCs derived from the two methods, we employed the pROC package in R. This package facilitates a non-parametric approach to compute the significance of the difference between the AUCs.

Drug target investigation For the top 50 protein-disease pairs identified by HDL-C in male and female cohorts, we systematically investigated available drugs targeting these proteins using the DrugBank and Drugs.com databases. It aimed to identify therapeutic opportunities by classifying the drug–protein–disease combinations into four main categories:New, druggable, re-purposing and validated. A protein–disease pair was considered validated if there was an existing drug known to influence both the protein and the disease. Validated pairs were further classified into ”Matched +” if the drug's impact on the protein and disease was consistent with the effect direction observed in HDL-C analysis, and ”Matched -” if the effect direction differed from the HDL-C estimation. If a drug targeted the protein but was approved for treating a different disease than the one identified by HDL-C, it was classified as a repurposing opportunity. This indicates potential for expanding the drug's use to new therapeutic areas. Protein–disease pairs were labeled as druggable if the protein is currently under clinical evaluation or considered viable for development into small-

molecule therapies, regardless of existing drug approval. If no known drugs were available for a given protein–disease pair, it was classified as ”New,” representing a novel therapeutic target for further exploration. This approach allowed for the identification of potentially actionable therapeutic targets based on the sex-specific results of the HDL-C analysis.

Code availability

HDL-C is included in the HDL project available at https://github.com/YuyingLi-X/HDL-C. COLOC and SuSiE software are available at https://chr1swallace.github.io/coloc/. PLINK 2.0 (https://www.cog-genomics.org/plink/2.0/) was used to extract individual-level data of imputed SNPs from the UKBB. PLINK 1.9 (https://www.cog-genomics.org/plink/) and LDAK (http://dougspeed.com/ldak/) were used in LD correlation calculation and simulations. REGSCAN is available at https://genomics.ut.ee/en/tools.

Data availability

The individual-level genotype and phenotype data are available by application from the UKBB (http://www.ukbiobank.ac.uk/). The UKBB GWAS summary statistics by the Neale laboratory can be obtained from http://www.nealelab.is/uk-biobank/. The UKB-PPP proteogenomic results and summary association data are available through an interactive portal at http://ukb-ppp.gwas.eu. Source data are provided in this paper.

Acknowledgements

X.S. was in receipt of a National Natural Science Foundation of China (NSFC) grant (No. 12171495), a National Key Research and Development Program grant (No. 2022YFF1202105), and a Swedish Research Council (Vetenskapsrådet) grant (No. 2022-01309).

Author contributions

X.S. and Y.P. initiated and supervised the study. Y.L., R.Z., Z.Y., and T.L. performed the analysis. Y.L., Y.P., and X.S. contributed to method development. Y.L. and X.S. wrote the paper, and all the authors approved the final version.

Competing interests statement

The authors declare no competing financial interests.

References

[1] Uffelmann, E. *et al.* Genome-wide association studies. *Nature Reviews Methods Primers* **1**, 1–21 (2021). URL https://www.nature.com/articles/s43586-021-00056-9.

[2] Schmitt, A. D., Hu, M. & Ren, B. Genome-wide mapping and analysis of chromosome architecture. *Nature Reviews Molecular Cell Biology* **17**, 743–755 (2016). URL http://www.nature.com/articles/nrm.2016.104.

[3] Visscher, P. M. *et al.* 10 Years of GWAS Discovery: Biology, Function, and Translation. *American Journal of Human Genetics* **101**, 5–22 (2017). URL https://www.ncbi.nlm.nih.gov/pmc/articles/PMC5501872/.

[4] Folkersen, L. *et al.* Genomic and drug target evaluation of 90 cardiovascular proteins in 30,931 individuals. *Nature Metabolism* **2**, 1135–1148 (2020). URL https://www.nature.com/articles/s42255-020-00287-2. Publisher: Nature Publishing Group.

[5] Repetto, L. *et al.* The genetic landscape of neuro-related proteins in human plasma. *Nature Human Behaviour* **8**, 2222–2234 (2024). URL https://www.nature.com/articles/s41562-024-01963-z. Publisher: Nature Publishing Group.

[6] Anderson, N. L. & Anderson, N. G. The human plasma proteome: history, character, and diagnostic prospects. *Molecular & cellular proteomics: MCP* **1**, 845–867 (2002).

[7] Emilsson, V. *et al.* Co-regulatory networks of human serum proteins link genetics to disease. *Science (New York, N.Y.)* **361**, 769–773 (2018).

[8] Sun, B. B. *et al.* Genomic atlas of the human plasma proteome. *Nature* **558**, 73–79 (2018). URL https://www.nature.com/articles/s41586-018-0175-2.

[9] Suhre, K., McCarthy, M. I. & Schwenk, J. M. Genetics meets proteomics: perspectives for large population-based studies. *Nature Reviews Genetics* **22**, 19–37 (2021). URL https://www.nature.com/articles/s41576-020-0268-2.

[10] Sun, B. B. *et al.* Plasma proteomic associations with genetics and health in the UK Biobank. *Nature* **622**, 329–338 (2023). URL https://www.nature.com/articles/s41586-023-06592-6.

[11] Benson, M. D. *et al.* Genetic Architecture of the Cardiovascular Risk Proteome. *Circulation* **137**, 1158–1172 (2018).

[12] Zhernakova, D. V. *et al.* Individual variations in cardiovascular-disease-related protein levels are driven by genetics and gut microbiome. *Nature Genetics* **50**, 1524–1532 (2018).

[13] Yao, C. *et al.* Genome-wide mapping of plasma protein QTLs identifies putatively causal genes and pathways for cardiovascular disease. *Nature Communications* **9**, 3268 (2018).

[14] Giambartolomei, C. *et al.* Bayesian Test for Colocalisation between Pairs of Genetic Association Studies Using Summary Statistics. *PLOS Genetics* **10**, e1004383 (2014). URL https://journals.plos.org/plosgenetics/article?id=10.1371/journal.pgen.1004383. Publisher: Public Library of Science.

[15] Wallace, C. Eliciting priors and relaxing the single causal variant assumption in colocalisation analyses. *PLOS Genetics* **16**, e1008720 (2020). URL https://journals.plos.org/plosgenetics/article?id=10.1371/journal.pgen.1008720. Publisher: Public Library of Science.

[16] Miller, A. J. Selection of Subsets of Regression Variables. *Journal of the Royal Statistical Society. Series A (General)* **147**, 389–425 (1984). URL https://www.jstor.org/stable/2981576. Publisher: [Royal Statistical Society, Wiley].

[17] Asimit, J. L. *et al.* Stochastic search and joint fine-mapping increases accuracy and identifies previously unreported associations in immune-mediated diseases. *Nature Communications* **10**, 3216 (2019). URL https://www.nature.com/articles/s41467-019-11271-0. Publisher: Nature Publishing Group.

[18] Wang, G., Sarkar, A., Carbonetto, P. & Stephens, M. A Simple New Approach to Variable Selection in Regression, with Application to Genetic Fine Mapping. *Journal of the Royal Statistical Society Series B: Statistical Methodology* **82**, 1273–1300 (2020). URL https://academic.oup.com/jrsssb/article/82/5/1273/7056114.

[19] Zhu, X. & Stephens, M. BAYESIAN LARGE-SCALE MULTIPLE REGRESSION WITH SUMMARY STATISTICS FROM GENOME-WIDE ASSOCIATION STUDIES. *The annals of applied statistics* **11**, 1561–1592 (2017). URL https://www.ncbi.nlm.nih.gov/pmc/articles/PMC5796536/.

[20] Wallace, C. A more accurate method for colocalisation analysis allowing for multiple causal variants. *PLOS Genetics* **17**, e1009440 (2021). URL https://journals.plos.org/plosgenetics/article?id=10.1371/journal.pgen.1009440. Publisher: Public Library of Science.

[21] Ning, Z., Pawitan, Y. & Shen, X. High-definition likelihood inference of genetic correlations across human complex traits. *Nature Genetics* **52**, 859–864 (2020). URL http://www.nature.com/articles/s41588-020-0653-y.

[22] Li, Y., Pawitan, Y. & Shen, X. An enhanced framework for local genetic correlation analysis. *Nature Genetics* **57**, 1053–1058 (2025). URL https://www.nature.com/articles/s41588-025-02123-3. Publisher: Nature Publishing Group.

[23] Ly, D., Forman, D., Ferlay, J., Brinton, L. A. & Cook, M. B. An International Comparison of Male and Female Breast Cancer Incidence Rates. *International journal of cancer. Journal international du cancer* **132**, 1918–1926 (2013). URL https://www.ncbi.nlm.nih.gov/pmc/articles/PMC3553266/.

[24] Bray, F. *et al.* Global cancer statistics 2022: GLOBOCAN estimates of incidence and mortality worldwide for 36 cancers in 185 countries. *CA: A Cancer Journal for Clinicians* **74**, 229–263 (2024). URL https://onlinelibrary.wiley.com/doi/abs/10.3322/caac.21834. _eprint: https://onlinelibrary.wiley.com/doi/pdf/10.3322/caac.21834.

[25] Karaman, M. W. *et al.* A quantitative analysis of kinase inhibitor selectivity. *Nature Biotechnology* **26**, 127–132 (2008).

[26] Lories, R. J. & Luyten, F. P. The bone–cartilage unit in osteoarthritis. *Nature Reviews Rheumatology* **7**, 43–49 (2011). URL https://www.nature.com/articles/nrrheum.2010.197. Publisher: Nature Publishing Group.

[27] Jura-Półtorak, A., Szeremeta, A., Olczyk, K., Zoń-Giebel, A. & Komosińska-Vassev, K. Bone Metabolism and RANKL/OPG Ratio in Rheumatoid Arthritis Women Treated with TNF-α Inhibitors. *Journal of Clinical Medicine* **10**, 2905 (2021). URL https://www.ncbi.nlm.nih.gov/pmc/articles/PMC8267676/.

[28] Shangguan, L., Ding, M., Wang, Y., Xu, H. & Liao, B. Denosumab ameliorates osteoarthritis by protecting cartilage against degradation and modulating subchondral bone remodeling. *Regenerative Therapy* **27**, 181–190 (2024).

[29] Nakashima, T., Wada, T. & Penninger, J. M. RANKL and RANK as novel therapeutic targets for arthritis. *Current Opinion in Rheumatology* **15**, 280–287 (2003).

[30] Lu, J. *et al.* Current comprehensive understanding of denosumab (the RANKL neutralizing antibody) in the treatment of bone metastasis of malignant tumors, including pharmacolog-

ical mechanism and clinical trials. *Frontiers in Oncology* **13**, 1133828 (2023). URL https://www.ncbi.nlm.nih.gov/pmc/articles/PMC9969102/.

[31] Kjolby, M. *et al.* Sort1, encoded by the cardiovascular risk locus 1p13.3, is a regulator of hepatic lipoprotein export. *Cell Metabolism* **12**, 213–223 (2010).

[32] Musunuru, K. *et al.* From noncoding variant to phenotype via SORT1 at the 1p13 cholesterol locus. *Nature* **466**, 714–719 (2010). URL https://www.ncbi.nlm.nih.gov/pmc/articles/PMC3062476/.

[33] Bycroft, C. *et al.* The UK Biobank resource with deep phenotyping and genomic data. *Nature* **562**, 203–209 (2018). URL https://www.nature.com/articles/s41586-018-0579-z. Publisher: Nature Publishing Group.

[34] Haller, T., Kals, M., Esko, T., Mägi, R. & Fischer, K. RegScan: a GWAS tool for quick estimation of allele effects on continuous traits and their combinations. *Briefings in Bioinformatics* **16**, 39–44 (2015). URL https://doi.org/10.1093/bib/bbt066.

Supplementary Figures

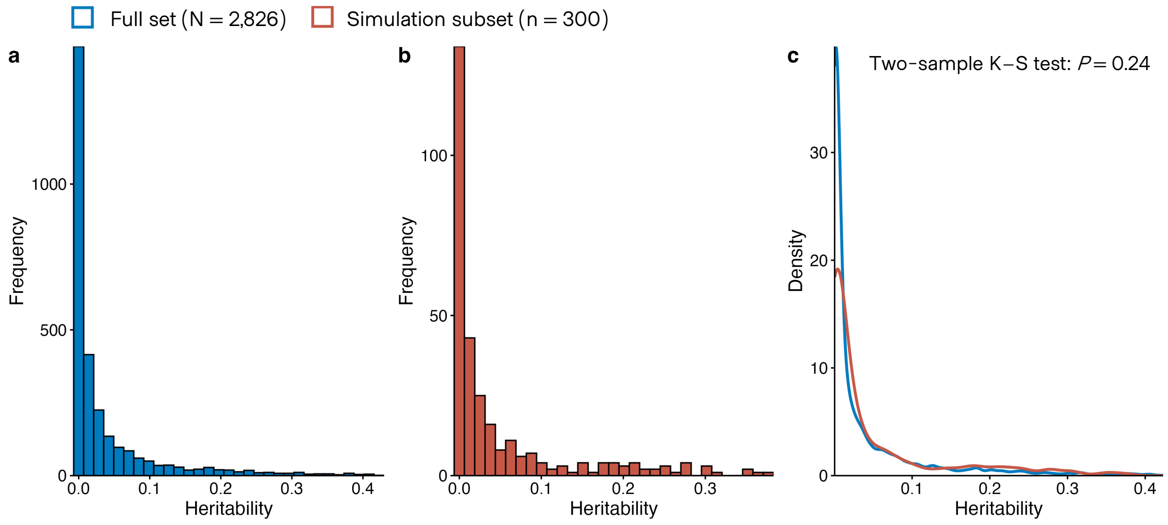

Supplementary Figure 1. Distribution of single SNP cis heritability estimates for plasma protein. The empirical distribution of the estimated heritability for the lead (top) variant in each cis pQTL region. (a) Histogram of all 2,826 cis pQTL regions. (b) Histogram of a random subset of 300 cis pQTL regions drawn without replacement so that their heritability spectrum mirrors that of the full set. (c) Kernel density overlays of the complete (blue) and subset (red) distributions. The P-value was derived by a two sample Kolmogorov–Smirnov test, confirming that no systematic bias was introduced by down sampling.

Supplementary Tables

Supplementary Table 1. The AUC values and *P*-values of HDL-C, COLOC, and SuSiE in simulation analyses. **Method**: Specifies the method used (HDL-C, COLOC, or SuSiE); **h11**: The simulated heritability level for the disease trait; **rg_threshold**: The threshold for the true genetic correlation used to define colocalization; **AUC**: The area under the receiver operating characteristic curve, reflecting the method's ability to distinguish between colocalized and non-colocalized regions; **CausalSNPs**: Denotes the simulation scenario—either a single causal SNP or multiple causal SNPs per region.

Supplementary Table 2. Description of 200 ICD-10 coded diseases. This table provides detailed information for 200 disease phenotypes based on ICD-10 codes used in the UK Biobank analysis. **phenotype**: ICD-10 code corresponding to each disease; **description**: Full description of the disease; **variable_type**: Indicates whether the phenotype is categorical or numerical; **source**: Source of the phenotype definition (ICD-10); **n_non_missing**: Number of individuals with non-missing phenotype data; **n_missing**: Number of individuals with missing phenotype data; **n_controls**: Number of individuals without the disease; **n_cases**: Number of individuals diagnosed with the disease.

Supplementary Table 3. Description of 2,826 proteins from the UK Biobank Pharma Proteomics Project. This table summarizes annotations and summary association statistics for 2,826 plasma proteins profiled in the UK Biobank Pharma Proteomics Project (UKB-PPP), used in cis-pQTL analyses. **UKBPPP_ProteinID**: Unique identifier of the protein, formatted as
HGNC.symbol_UniProt_OID_Panel_chr.Position.
CHR, POS19, POS38: Chromosome and position of the top associated SNP in GRCh37 (POS19) and GRCh38 (POS38) coordinates; **REF, ALT**: Reference and alternative alleles for the top SNP; **rsid**: dbSNP identifier of the top SNP; **BETA, SE**: Estimated effect size and standard error from cis-pQTL association testing; **ALTFREQ**: Frequency of the alternative allele; **N**: Sample size used in the association analysis; **Z**: Z-score, computed as the ratio of BETA to SE; $\mathbf{h^2}$: SNP heritability of the top variant in each cis-region.

Supplementary Table 4. Colocalization results from HDL-C, COLOC, and SuSiE across sex-stratified UK Biobank populations. This Excel file contains top 50 significant colocalization analysis results between 2,826 proteins and 200 ICD-10 coded disease traits, stratified by sex. Results were obtained using three methods: HDL-C, COLOC, and SuSiE. Each method was applied separately to the male and female datasets from the UK Biobank. The file contains six sheets

- **HDL_male** and **HDL_female**: Local genetic correlation estimates from HDL-C, applied to the male and female datasets, respectively.
- **COLOC_male** and **COLOC_female**: Posterior probabilities of five hypotheses estimated by COLOC, based on sex-stratified GWAS summary statistics.
- **SuSiE_male** and **SuSiE_female**: Fine-mapped colocalization results using the SuSiE regression model, reporting credible sets and posterior support for shared causal variants in males and females.

Supplementary Table 5. Top 50 significant protein–disease associations from HDL-C analysis and cross-referencing with DrugBank. This table summarizes results from an extended HDL-C analysis in which we investigated the top 50 significant protein–disease associations separately in male and female cohorts. The combined set included 92 unique protein–disease pairs. Each entry includes local genetic correlation estimates and inference statistics from HDL-C, alongside DrugBank annotation for corresponding protein targets. The file contains four sheets: Matched, druggable,re-purposing, and new.
uniprot, **protein**: UniProt accession and gene/protein name of the cis-pQTL.
phenotype, **description.x**: ICD-10 code and disease description.
Heritability_1, **Heritability_2**: Local SNP heritability estimates for the protein and disease traits, respectively.
Genetic_Covariance, **Genetic_Correlation**: Local genetic covariance and correlation estimates from HDL-C.
Lower_bound_rg, **Upper_bound_rg**, **P**: Likelihood-based confidence interval and P-value for local genetic correlation.
Gender: Indicates whether the result is from the male or female cohort.
drugbank_id, **name**, **description.y**: DrugBank ID, compound name, and description of known compounds targeting the protein.
known_action, **gene_name**, **cellular_location**: Drug–target interaction metadata and protein localization from DrugBank.

Supplementary Table 1

Click here to access/download
Supplementary Material
Supplementary Table 1.xlsx

Supplementary Table 2

Click here to access/download
Supplementary Material
Supplementary Table 2.xlsx

Supplementary Table 3

Click here to access/download
Supplementary Material
Supplementary Table 3.xlsx

Supplementary Table 4

Click here to access/download
Supplementary Material
Supplementary Table 4.xlsx

Supplementary Table 5

Click here to access/download
Supplementary Material
Supplementary Table 5.xlsx

Cover Letter
Click here to access/download;Personal Cover;CoverLetter_250522.pdf

Prof. Xia Shen, PhD

Department of Medical Epidemiology and Biostatistics, Karolinska Institutet
Nobels väg 12A, SE-17 177, Stockholm, Sweden
Centre for Global Health Research, University of Edinburgh Medical School
Teviot Place, Edinburgh, EH8 9AG, UK
Biostatistics Lab, School of Life Sciences, Fudan University
Songhu Road 2005, Shanghai, China

xia.shen@ed.ac.uk

Thursday, 22 May 2025

To the Editor, Hongling Zhou
GigaScience

Dear Hongling:

We hereby submit our work, "*High-definition likelihood inference of colocalization reveals protein biomarkers for human complex diseases*," to *GigaScience* for publication as a Technical Note.

The method presented is called **HDL-C, i.e., high-definition likelihood for colocalization analysis**. Colocalization is one of the most essential analyses in current genetics, as it reveals important biomarkers for complex traits, which you can also tell from the citations of the state-of-the-art colocalization analysis methods, including COLOC (with 3,000 citations in 10 years) and SuSiE (with 300 citations in 4 years). **HDL-C is another extension of our HDL toolkit**, where the initial HDL method (genetic correlation analysis) was published in *Nature Genetics* (**52**, 859-864), the HDL-L method (local genetic correlation analysis) also in *Nature Genetics* (**57**, pp. 1053–1058), and the HDL-S method (stratified heritability analysis) currently under review (https://doi.org/10.21203/rs.3.rs-5204355/v1). Our main results are:

1. **HDL-C outperforms COLOC and SuSiE** in detecting genetic colocalization;
2. **HDL-C has a higher rediscovery rate** in independent samples, showing the **highest robustness**;
3. Application of **HDL-C prioritizes drug targets** for human complex diseases, as demonstrated by examples of both established drugs and potential new targets.

As this work directly improves the highly popular genetic analysis tools, we foresee a significant impact of our message. We believe the message ought to be disseminated widely, so we decided to submit it to the well-received open-access journal *GigaScience*.

The study has been selected for an oral presentation at the 2025 European Society of Human Genetics (ESHG) conference – an especially noteworthy distinction for first author Yuying Li, who will present the work as a PhD student.

Please see below for suggested and conflicted reviewers. We look forward to hearing from you.

Best regards,

Dr. Xia Shen

Suggested Referees:

1. Prof. **Xin Jin** (jinxin@genomics.cn) - Prof. Xin Jin is Vice Dean of BGI-Shenzhen and Chief Scientist in the field of population genomics at the BGI Life Science Research Institute, as well as Professor at South China University of Technology. With a strong background in genomics and bioinformatics, his expertise lies in large-scale genomic data analysis, population genetics, and precision health research. Given his leadership in both academic and translational genomics research, along with his contributions to high-impact national projects and bioinformatics training, Prof. Jin would be a highly qualified reviewer for our manuscript focused on genetic colocalization and protein biomarker discovery.
2. Prof. **Chaolong Wang** (chaolong@hust.edu.cn) - Prof. Chaolong Wang at Huangzhou University of Science and Technology is a recipient of the National Science Fund for Distinguished Young Scholars. Prof. Wang's research focuses on statistical population genetics and integrative genomic analysis, with key contributions to the genetic architecture of complex diseases and infectious disease modeling. Prof. Wang would be an excellent reviewer for our manuscript on local genetic correlation and protein biomarker discovery.
3. Prof. **Miaoxin Li** (limiaoxin@mail.sysu.edu.cn) - Prof. Miaoxin Li is the Director of the Department of Medical Informatics and a leading expert in statistical genetics and bioinformatics. His group has developed widely adopted tools such as the KGG software suite. Given his extensive expertise in genetic epidemiology and method development, particularly in computational approaches for gene mapping and colocalization, Prof. Li would be a highly suitable and insightful reviewer for our manuscript.

Referees to Exclude:

As we directly challenge the popular tools COLOC and SuSiE, please exclude the main authors of those tools from reviewing this work, including **Claudia Giambartolomei, Damjan Vukcevic, Eric E. Schadt, Lude Franke, Aroon D. Hingorani, Chris Wallace, Vincent Plagnol,** and their close colleagues.
