# Supplementary material for: High-definition likelihood inference of genetic colocalization reveals protein biomarkers for human complex diseases
Source: Gigascience. 2026 Jan 23;15:giaf155. doi: 10.1093/gigascience/giaf155 (PMC12916012; doi:10.1093/gigascience/giaf155)
Supplement: giaf155_GIGA-D-25-00203_Revision_1 [file giaf155_giga-d-25-00203_revision_1.pdf]

GigaScience

High-definition likelihood inference of colocalization reveals protein biomarkers for human complex diseases

--Manuscript Draft--

**Manuscript Number:**	GIGA-D-25-00203R1
**Full Title:**	High-definition likelihood inference of colocalization reveals protein biomarkers for human complex diseases
**Article Type:**	Technical Note
**Funding Information:**	National Natural Science Foundation of China (12171495) — Dr. Xia Shen<br>Key Technologies Research and Development Program (2022YFF1202105) — Dr. Xia Shen<br>Vetenskapsrådet (2022-01309) — Dr. Xia Shen
**Abstract:**	Background: Genetic colocalization analysis is essential for understanding the shared genetic basis between phenotypic traits. Such an analysis is particularly useful for identifying plasma proteins with potential as therapeutic targets or clinical biomarkers. Improvements to existing tools are needed for more accurate inference of potentially causal biomarkers.<br>Findings: We develop HDL-C, a high-definition likelihood inference method for genetic colocalization analysis. Based on simulations and observed rediscovery rates in real data analyses, we demonstrate that the HDL-C approach outperforms state-of-the-art methods, COLOC, SuSiE, and SharePro, in detecting genetic colocalization, thus enabling a more complete understanding of genetic connections at specific loci. Analyses of the top 50 protein–disease pairs identified by HDL-C in the male and female cohorts of the UK Biobank uncovered 40 previously validated drug-protein–disease combinations with approved drugs matching the phenotypes and 62 combinations with potential drug repurposing opportunities. Additionally, we identified 63 novel protein–disease pairs that suggest promising candidates for future therapeutic interventions.<br>Conclusion: This research establishes a robust framework for detecting colocalization signals, enabling the prioritization of disease-relevant protein targets and informing therapeutic development strategies.
**Corresponding Author:**	Xia Shen<br>Fudan University<br>Guangzhou, Please select CHINA
**Corresponding Author Secondary Information:**	
**Corresponding Author's Institution:**	Fudan University
**Corresponding Author's Secondary Institution:**	
**First Author:**	Yuying Li
**First Author Secondary Information:**	
**Order of Authors:**	Yuying Li<br>Ranran Zhai<br>Zhijian Yang<br>Ting Li<br>Yudi Pawitan<br>Xia Shen

Powered by Editorial Manager® and ProduXion Manager® from Aries Systems Corporation

Order of Authors Secondary Information:	
Response to Reviewers:	See the Responses to Reviewers in the PDF document.
Additional Information:	
Question	Response
Are you submitting this manuscript to a special series or article collection?	No
**Experimental design and statistics**<br><br>Full details of the experimental design and statistical methods used should be given in the Methods section, as detailed in our Minimum Standards Reporting Checklist. Information essential to interpreting the data presented should be made available in the figure legends.<br><br>Have you included all the information requested in your manuscript?	Yes
**Resources**<br><br>A description of all resources used, including antibodies, cell lines, animals and software tools, with enough information to allow them to be uniquely identified, should be included in the Methods section. Authors are strongly encouraged to cite Research Resource Identifiers (RRIDs) for antibodies, model organisms and tools, where possible.<br><br>Have you included the information requested as detailed in our Minimum Standards Reporting Checklist?	Yes
**Availability of data and materials**<br><br>All datasets and code on which the conclusions of the paper rely must be either included in your submission or deposited in publicly available repositories (where available and ethically appropriate), referencing such data using a unique identifier in the references and in	Yes

Powered by Editorial Manager® and ProduXion Manager® from Aries Systems Corporation

the “Availability of Data and Materials” section of your manuscript.<br><br>Have you have met the above requirement as detailed in our Minimum Standards Reporting Checklist?	
GigaScience has policies and guidelines in place for the use of generative AI-writing tools such as ChatGPT. If you have used such writing tools to assist with writing the manuscript this must be declared and cited in the text. Authors should not list AI-writing tools and other AI-assisted technologies as an author or co-author and should acknowledge that they are fully responsible for text generated or refined by AI-writing tools.<p><br><br>A summary of use (particularly in the introduction or among methods) needs to be included at the end of the paper, and the outputs should also be included as a supplementary file hosted in GigaDB or other open repositories. Please read our guidelines for more information. <p><br><br>By submitting to GigaScience, you are aware of the journal's AI-writing tools policy, and if you have declared use of such tools below, you have acknowledged this where appropriate in your manuscript and have made a summary of use and outputs available. <p><br><b>AI-assisted writing tools have been used in the preparation of this manuscript?	No

Powered by Editorial Manager® and ProduXion Manager® from Aries Systems Corporation

This piece of the submission is being sent via mail.

Manuscript

Click here to access/download;Manuscript;Revision_FullText_251118.pdf

High-definition likelihood inference of colocalization reveals protein biomarkers for human complex diseases

Yuying Li[1,2,†], Ranran Zhai[2,3,†],Zhijian Yang[2,5], Ting Li[2,3], Yudi Pawitan[1], Xia Shen[1,2,3,4,*]

[1]Department of Medical Epidemiology and Biostatistics, Karolinska Institutet, Stockholm, Sweden

[2]Center for Intelligent Medicine Research, Greater Bay Area Institute of Precision Medicine (Guangzhou), Fudan University, Guangzhou, China

[3]State Key Laboratory of Genetic Engineering, Center for Evolutionary Biology, School of Life Sciences, Fudan University, Shanghai, China

[4]Centre for Global Health Research, Usher Institute, University of Edinburgh, Edinburgh, UK

[5]Institute for Molecular Medicine Finland (FIMM), HiLIFE, University of Helsinki, Helsinki, Finland

[*]Correspondence should be addressed to: shenxia911@gmail.com

[†]These authors contributed equally to this work.

Abstract

Background: Genetic colocalization analysis is essential for understanding the shared genetic basis between phenotypic traits. Such an analysis is particularly useful for identifying plasma proteins with potential as therapeutic targets or clinical biomarkers. Improvements to existing tools are needed for more accurate inference of potentially causal biomarkers.

Findings: We develop HDL-C, a high-definition likelihood inference method for genetic colocalization analysis. Based on simulations and observed rediscovery rates in real data analyses, we demonstrate that the HDL-C approach outperforms state-of-the-art methods, COLOC, SuSiE, and SharePro, in detecting genetic colocalization, thus enabling a more complete understanding of genetic connections at specific loci. Analyses of the top 50 protein–disease pairs identified by HDL-C in the male and female cohorts of the UK Biobank uncovered 40 previously validated drug-protein–disease combinations with approved drugs matching the phenotypes and 62 combinations with potential drug repurposing opportunities. Additionally, we identified 63 novel protein–disease pairs that suggest promising candidates for future therapeutic interventions.

Conclusion: This research establishes a robust framework for detecting colocalization signals, enabling the prioritization of disease-relevant protein targets and informing therapeutic development strategies.

Keywords

HDL-C, Colocalization, Genetic correlation, Proteomics, Complex diseases, Therapeutic targets

Introduction

Genetic influences underlying human diseases and traits remain an important area of investigation in genomics. Genome-wide association studies (GWAS) have significantly advanced genetic research by identifying numerous genomic regions linked to various traits and disease susceptibilities [1–5]. A key aspect of this exploration is understanding how variations in the genome correlate with variations in phenotypic traits, including those associated with complex diseases. This understanding not only reveals the genetic architecture of these traits but also helps to identify potential therapeutic targets and biomarkers for disease prediction and management.

Plasma proteins, given their critical roles in various biological processes and disease pathways, serve as valuable biomarkers and therapeutic targets. The measured proteome encompasses pro-

teins secreted or shed into the blood circulation, which play major roles in various molecular processes and mediate cross-tissue communication [6]. Their expression levels, often influenced by genetic variations, can provide insights into the molecular mechanisms of diseases. Recent technological advancements in high-throughput quantification of circulating proteins have led to large-scale studies of protein quantitative trait loci (pQTL) [7–13]. These studies have highlighted the potential of associating protein levels with DNA sequence variants that colocalize with risk alleles for common diseases. Such colocalizations can reveal disease-associated pathways, offering novel insights into drug targets and translational biomarkers.

Therefore, methods for more accurate inference of genetic colocalization are essential for the joint analysis of molecular traits and complex diseases. COLOC is one of the most widely used methods for colocalization analysis, which aims to detect genetic colocalization between pairs of traits, such as the analysis of complex traits at specific molecular quantitative trait loci (QTL) [14]. This Bayesian model makes restrictive assumptions about the underlying shared genetic architecture at the given locus, e.g., one causal variant per trait. Such an assumption may not always hold in real-world datasets. While extensions of COLOC [15] using conditional regression have been attempted to address the issue of multiple variants, they rely on assumptions of independence among causal variants, which may not hold true, especially in the presence of extensive linkage disequilibrium (LD)[16,17]. The Sum of Single Effects (SuSiE) framework, integrated into the COLOC package, has addressed these limitations by enabling robust fine-mapping of multiple causal variants [18–20]. However, this approach primarily focuses on fine-mapping rather than quantifying colocalization itself. A more recent method is SharePro, which explicitly models multiple causal variants and joint fine-mapping [21].

As an alternative strategy, we propose the inference of a *sufficiently high* estimated regional genetic correlation (r_G) between two phenotypes at a specific genomic locus to detect genetic colocalization. Unlike existing methods, this strategy quantifies colocalization through a single genetic correlation parameter without strict assumptions about the underlying genetic architecture. We previously developed the high-definition likelihood (HDL) method as a robust approach for estimating genetic correlations using GWAS summary statistics[22] and the recent local version of this method, HDL-L, to estimate local genetic correlations[23]. This advancement enables more granular exploration of genetic correlations at specific loci. Nevertheless, for colocalization detection, inference must be based on a conditional likelihood given a sufficiently high regional genetic correlation estimate.

In this study, we (i) develop the theory and implement high-definition likelihood for colocalization inference (HDL-C), (ii) demonstrate that HDL-C performs better than COLOC, SuSiE, and

SharePro in detecting genetic colocalization, and (iii) apply the HDL-C method to investigate the colocalization between plasma proteins and complex diseases using data from the UK Biobank. Specifically, we prioritize drug targets focusing on 2,826 plasma proteins and their colocalization with 200 diseases. This approach offers a new opportunity to explore the genetic basis of disease-protein associations, potentially uncovering novel insights into disease mechanisms.

Results

Overview of the HDL-C method

We define regional colocalization as the presence of a nonzero local genetic correlation between two traits. When the estimated correlation r_G equals zero, the local genetic effects are uncorrelated and there is no evidence of colocalization. Conversely, a significantly large r_G indicates that the traits share a consistent pattern of genetic effects within the region. In practice, one may regard a region as colocalized either when r_G is significantly different from zero, or when it exceeds a prespecified threshold $r_0 > 0$ that reflects a biologically meaningful level of correlation.

To formally test whether the local genetic correlation exceeds such a threshold, we develop the **HDL-C** method—a constrained likelihood ratio framework built upon the high-definition likelihood model to test genetic colocalization.

By definition, $r_G = h_{12}/\sqrt{h_1^2 h_2^2}$, where h_1^2 and h_2^2 denote the local SNP-heritabilities of the two traits, and h_{12} their local genetic covariance. We consider the likelihood function $\mathcal{L}(h_1^2, h_2^2, h_{12} \mid \mathbf{z}_1, \mathbf{z}_2)$ for the regional genetic association Z-scores $(\mathbf{z}_1, \mathbf{z}_2)$, derived under the HDL multivariate normal model. We test whether the magnitude of the local genetic correlation exceeds a biologically meaningful threshold $r_0 \in [0, 1)$:

$$H_0 : |r_G| \leq r_0 \quad \Longleftrightarrow \quad |h_{12}| \leq r_0 \sqrt{h_1^2 h_2^2}, \qquad H_A : |r_G| > r_0.$$

The null hypothesis therefore defines a bounded composite region in the parameter space. The *profile* likelihood of the genetic covariance is $\mathcal{L}_p(h_{12}) = \max_{h_1^2, h_2^2} \mathcal{L}(\theta) = \mathcal{L}(h_{12}, \hat{h}_1^2, \hat{h}_2^2)$, where $\hat{h}_1^2$ and $\hat{h}_2^2$ are the MLEs of the heritabilities. The LRT statistic for genetic covariance is formulated as

$$\Lambda = -2 \ln \left[\frac{\sup \mathcal{L}_p(h_{12}) : |h_{12}| \leq r_0 \sqrt{\hat{h}_1^2 \hat{h}_2^2}}{\sup \mathcal{L}_p(h_{12}) : |h_{12}| \leq \sqrt{\hat{h}_1^2 \hat{h}_2^2}} \right].$$

In practice, we profile over h_{12} while fixing h_1^2 and h_2^2 at their unconstrained MLEs, which pre-

serves the null constraint $|h_{12}| \leq B$, where $B = r_0\sqrt{\widehat{h}_1^2\widehat{h}_2^2}$. Because the null involves an inequality constraint, the asymptotic null distribution of Λ follows a mixture of

$$\Lambda \xrightarrow{H_0} \tfrac{1}{2}\chi_0^2 + \tfrac{1}{2}\chi_1^2,$$

i.e., a 50:50 mixture of a point mass at zero and a χ_1^2 distribution [24].

This procedure directly tests whether the local genetic correlation exceeds a biologically meaningful threshold r_0, rather than testing for zero correlation. It can equivalently be viewed as assessing whether the profile-likelihood confidence interval for r_G lies entirely outside the interval $[-r_0, r_0]$. In this sense, HDL-C provides a likelihood-based test of regional colocalization strength, complementing HDL-L, which estimates the magnitude of local correlation.

In contrast to standard colocalization methods, which typically model variant-level causal probabilities under strong prior assumptions, HDL-C exploits the summary-level multivariate Gaussian structure of Z-scores and the polygenic covariance encoding in the LD score matrix. HDL-C thus provides a high-dimensional, likelihood-based inference procedure for genetic colocalization that requires only GWAS summary statistics and an LD reference.

In practice, the choice of r_0 reflects the minimum degree of local genetic sharing required to declare colocalization. In biomarker discovery, where near-identical genetic architectures are desired, we recommend a conservative range of $r_0 \in 0.5, 0.8$; for exploratory scans allowing partial sharing, $r_0 \approx 0$ is reasonable. Based on simulations, the empirical performance of HDL-C was generally robust across choices of r_0, with only minor power gains observed for $r_0 = 0$ under low genetic correlations. Therefore, we suggest reporting results from $r_0 = 0.5$ as a balanced default, accompanied by p-values and likelihood-based estimates of local genetic correlation to jointly assess statistical significance and biological concordance.

To evaluate the performance of HDL-C, we conducted a series of simulation studies comparing it with COLOC[14], SuSiE[20] and SharePro[21]. Given that COLOC inherently assumes a single causal single nucleotide polymorphism (SNP) per region, we designed simulations in two scenarios: (i) each region with a single causal SNP and (ii) each region was simulated under multiple causal-variant scenarios, assuming either 3, 5, or 10% of SNPs as causal.. In addition, to analyze protein molecules and complex diseases, we examined different levels of true regional heritability for the disease trait. For cis-pQTLs, we estimated the heritability of the top SNP in each cis-region (see **Methods**) and then randomly selected 300 cis-pQTLs reflecting the heritability distribution of the full set of 2,826 cis-pQTLs (Supplementary Fig. 1). This subset approach was used to manage computational efficiency, as conducting simulations in all pQTL regions would be excessively computationally in-

tensive. In each simulation replicate, we generated phenotypic data for two traits and estimated their local genetic correlation. The true effect sizes of the causal variants were drawn from a bivariate normal distribution, given the true genetic correlation (see Methods). The summary association statistics were then calculated from a genome-wide association analysis by regressing the simulated phenotypic data against the corresponding genotypes at each SNP.

HDL-C outperforms state-of-the-art methods in detecting genetic colocalization

Under the assumption of 10% causal SNPs, HDL-C consistently outperformed COLOC, SuSiE, and SharePro across a range of true genetic correlation thresholds (0–1) and regional heritability levels (**Fig. 1a**). We evaluated two HDL-C thresholds, denoted HDL-C(0) and HDL-C(0.5), corresponding to increasingly stringent definitions of colocalization. The Area Under the Curve (AUC) of HDL-C(0) ranged from 0.92 to 0.98, compared with 0.82–0.99 for HDL-C(0.5), 0.70–0.93 for COLOC, 0.65–0.73 for SuSiE, and 0.53–0.65 for SharePro. More specifically, HDL-C achieved higher true positive rates (TPR) than COLOC, SuSiE, and SharePro at both 5% and 10% false positive rate (FPR) thresholds (Supplementary Fig. 2). Under the single-causal-variant setting, where true local genetic correlation is expected to be ± 1 when colocalization exists, COLOC achieved the highest AUC, followed by SharePro, HDL-C, and SuSiE (**Fig. 1b**). When regional heritability for the disease trait increased to 0.1 in the corresponding cis-pQTL region, HDL-C performed comparably to SharePro, with AUCs of 0.93 and 0.92, respectively. In scenarios with three and five causal variants (**Fig. 1c,d**), HDL-C again delivered the strongest overall performance across all heritability levels. Notably, when the number of causal variants increased and the true genetic correlation weakened, HDL-C(0) outperformed the more conservative HDL-C(0.5). To assess computational efficiency, we benchmarked per-locus execution time across 50 simulation replicates in 300 cis-pQTL regions (15,000 runs per method; Supplementary Fig. 3). All analyses were performed on a single CPU core without parallelization, using a uniform memory allocation of 8GB for all methods. Median runtime was 0.007s for COLOC, 0.445s for SuSiE, 2.04s for HDL-C, and 4.93s for SharePro. Although HDL-C is not the fastest, it completes within a few seconds per locus and exhibits stable upper-tail performance (95th percentile < 3s). At this rate, analysis of 1,000 loci requires approximately 34 minutes on a single core. The speed of COLOC reflects its simpler single-causal-variant model.

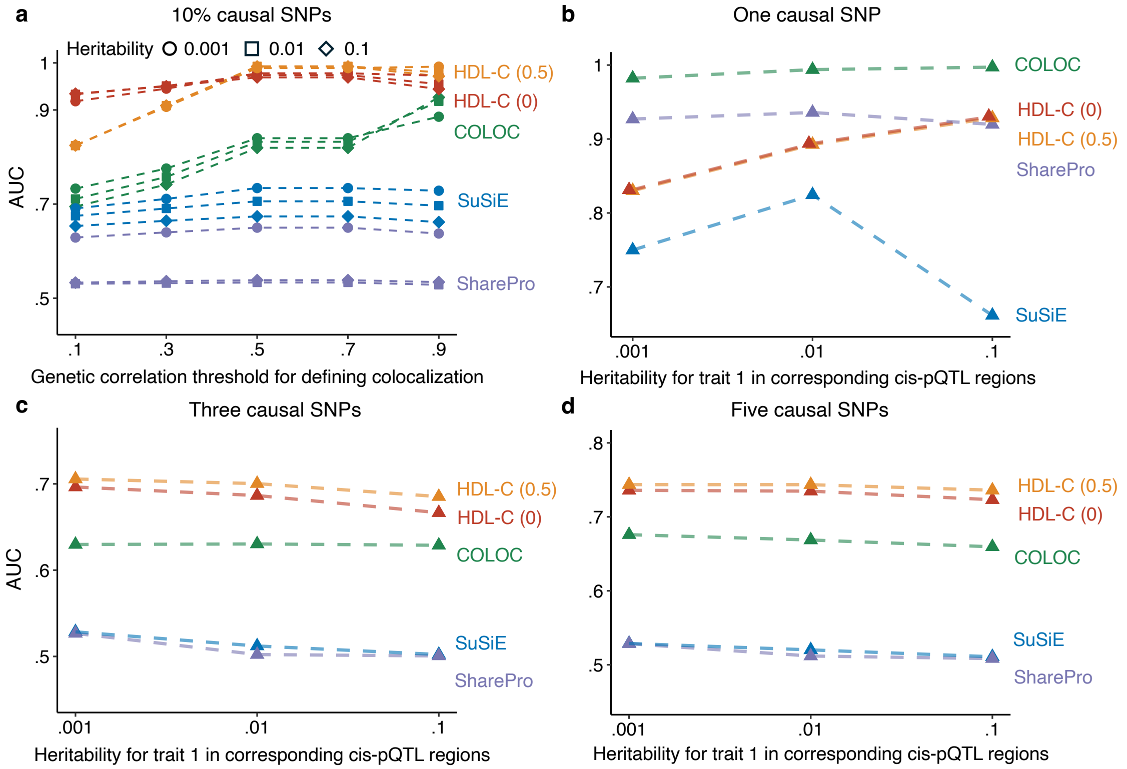

Figure 1: Performance Comparison of HDL-C, COLOC, SuSiE and SharePro in detecting genetic colocalization by AUC. Panels show the area under the ROC curve (AUC) for different methods across simulation settings. The colocalization level was simulated across different true genetic correlation values. (a) Results for 10% causal SNPs, plotted against the genetic correlation threshold used to define colocalization We examined three different levels of true regional heritability for disease traits corresponding to cis-pQTL regions. (b-d) Results for one, three, and five causal SNPs, respectively. HDL-C (0) refers to the setting in which colocalization is assessed by testing whether the local genetic correlation equals zero ($r_G = 0$), whereas HDL-C (0.5) corresponds to a more stringent criterion that tests whether the local genetic correlation is less than or equal to 0.5 ($r_G \leq 0.5$).

HDL-C has higher rediscovery rates in two independent samples

To demonstrate our theory in real data analyses, we evaluated their ability to detect genetic colocalization between 200 ICD-10-coded diseases and 2,826 proteins in the UK Biobank. We focused on cis-pQTL regions to explore the shared genetic architecture between these diseases and proteins in male and female populations. We used two validation settings. In the first setting, we used female data for training and male data for testing. In the second setting, we used male data for training and female data for testing. This design allowed us to directly evaluate the reproducibility of findings across sex-stratified cohorts. We provide detailed descriptions of the diseases and their associated proteins in Supplementary Tables 2-3. The top 50 significant colocalization results from HDL-C, COLOC, SuSiE, and SharePro analyses were selected from the training set, and we examined the rediscovery rates (RDR) for these methods in the test set as the proportion of overlapping results between the top 50 findings in the test and training sets (**Fig. 2**, Supplementary Table 4).

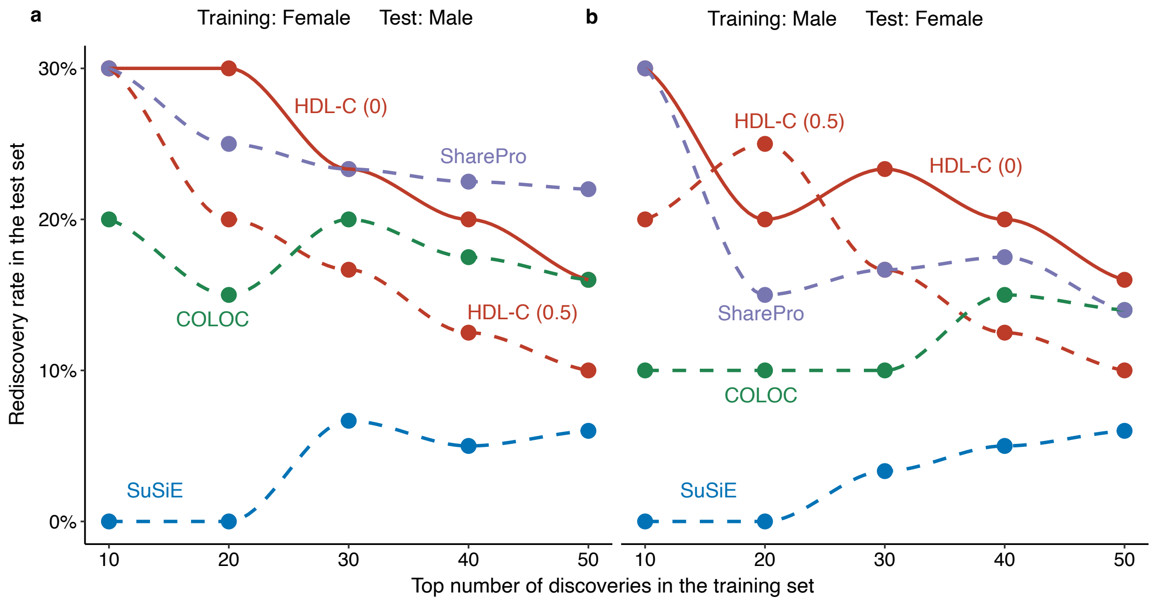

Figure 2: Rediscovery rates of HDL-C, COLOC, SuSiE, and SharePro under two validation settings. (a) Training was performed on the female dataset and testing on the male dataset. (b) Training was performed on the male dataset and testing on the female dataset. The x-axis shows the top N significant results selected in the training set. The y-axis shows the proportion of these signals rediscovered in the test set. We applied HDL-C, COLOC, SuSiE, and SharePro on ICD-10 coded 200 diseases and 2,826 proteins summary association statistics in the UK Biobank male and female population.HDL-C (0) refers to the setting in which colocalization is assessed by testing whether the local genetic correlation equals zero ($r_G = 0$), whereas HDL-C (0.5) corresponds to a more stringent criterion that tests whether the local genetic correlation is less than or equal to 0.5 ($r_G \leq 0.5$).

HDL-C(0) achieved the highest rediscovery rates among all evaluated methods, except in the female-to-male validation, when the top discoveries exceeded 40, where SharePro performed slightly better(**Fig. 2**). Based on simulation results, this exception likely reflects that shared causal variants between males and females are often driven by a single dominant causal SNP rather than multiple shared signals. Overall, HDL-C(0.5) performed worse than HDL-C(0), indicating that most protein–disease associations exhibit weak and highly polygenic genetic architectures. Some diseases may have sex-specific patterns: This would influence the RDR[25,26], but not affect the comparison of performance between the methods. Also, in our sex-stratified GWAS analyses, the first 20 genetic principal components (PCs) were included to account for population structure. These PCs were derived from the entire UK Biobank cohort using genome-wide genotype data, making it unlikely that the observed local genetic correlations were artifacts of population stratification. Taken together, HDL-C(0) demonstrated superior reproducibility and robustness under these validation settings. COLOC and SuSiE could identify overlapping genetic loci, but often yielded lower RDR compared to HDL-C. SharePro achieved intermediate performance across both validation directions. This finding showed the advantage of HDL-C's likelihood-based framework in detecting robust colocalization when applied to real data.

HDL-C prioritizes drug targets for human complex diseases

We extended our analysis by investigating the top 50 genetically correlated protein–disease pairs identified by HDL-C(0) in the male and female subcohorts, respectively, resulting in 92 unique protein–disease combinations. Each of these combinations was cross-referenced with DrugBank (Supplementary Table 5). Integrating HDL-C discoveries with existing drug information, 40 validated drug-protein-disease combinations were identified where a given drug targets the same protein and treats the same disease or causes the same side effect ("Matched"). For all of the 40 matched combinations, the HDL-C inferred protein's causal effect directions were consistent with the corresponding drug action direction ("Matched $^+$") (**Fig. 3a**). We also identified 62 combinations where the drugs have different approved indications that differ from the diseases or side effects identified in the HDL-C results ("Re-purposing"), suggesting potential re-purposing opportunities. Furthermore, we discovered 63 protein–disease pairs where the proteins are not targeted by any drug in DrugBank ("New"), indicating potential novel therapeutic targets if the potential causal effects can be validated. In addition, we denoted 133 combinations as "Druggable", which means their ongoing evaluation in clinical trials or their viability for development into small-molecule therapies. We further showed the distribution of these drug-protein–phenotype combinations per protein (**Fig. 3b**).

For example, we observed that Tyrosine-protein kinase Fes/Fps (FES, UniProt P07332) exhibited a protective effect against chronic ischemic heart disease (ICD10: I25), as indicated by a significant local genetic correlation estimate of −0.99 (95% CI, −0.54 to −1.00) in females and −0.81 (95% CI, −0.49 to −1.00) in males (**Fig. 3c**). Fostamatinib - marketed as Tavalisse since its FDA approval on April 17, 2018 - was developed as a spleen tyrosine kinase (SYK) inhibitor for rheumatoid arthritis and immune thrombocytopenic purpura (ITP). However, studies have demonstrated that its active metabolite (R406) can also inhibit FES, a kinase implicated in the regulation of protective inflammation [27]. While fostamatinib's anti-inflammatory properties have been investigated for mitigating vascular damage (and even acute respiratory distress syndrome in severe COVID-19), recent clinical evidence points to an increased incidence of cardiovascular side effects, notably hypertension, that may exacerbate ischemic heart conditions. This aligns with our findings of genetic correlation.

Aminocaproic acid is an antifibrinolytic agent that, by inhibiting plasminogen activation, may potentiate the prothrombotic environment in individuals with elevated apolipoprotein(a) [Lp(a)]. Higher Lp(a) levels themselves are well-documented risk factors for atherosclerotic disease, including angina pectoris, due to Lp(a)'s structural similarity to plasminogen and resultant interference with normal fibrinolysis. Thus, when aminocaproic acid further restricts fibrinolysis, it can intensify the cardiovascular risk posed by elevated Lp(a), leading to an increased incidence or severity of angina pectoris. This mechanistic interplay aligns with our significant local genetic correlation finding (0.94 with 95% CI (0.70,1.00) between Lp(a) and Angina pectoris (**Fig. 3c**), underscoring the shared risk pathway involving fibrinolysis inhibition and Lp(a)-related atherogenesis.

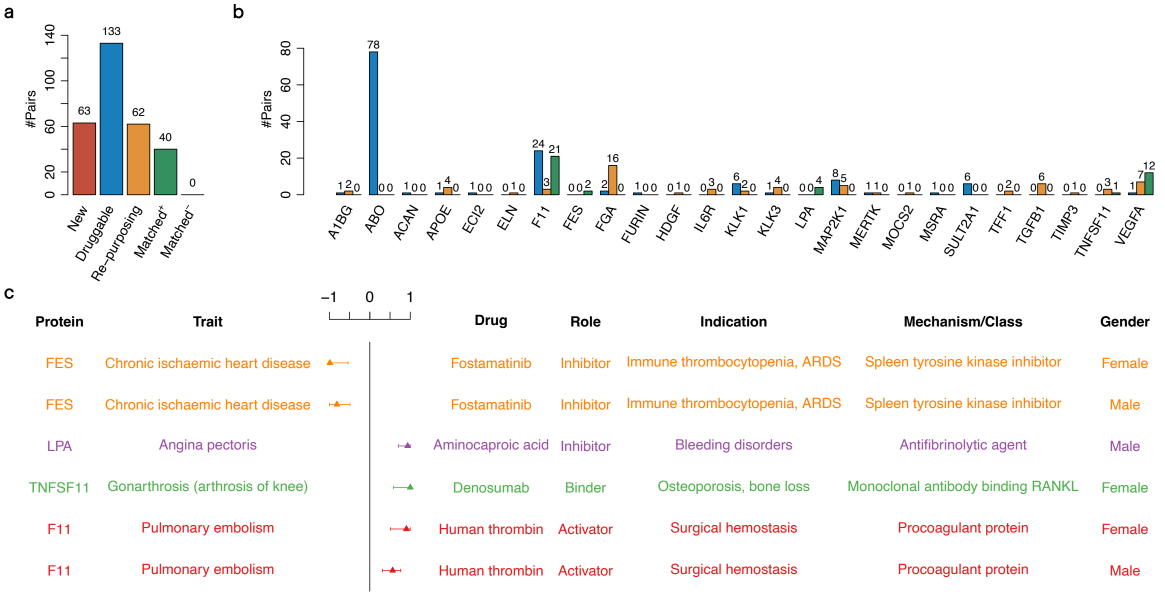

Figure 3: Drug targets inferred by local genetic correlation analysis. (a) Drug-protein–phenotype combinations identified from the top 50 HDL-C results in separate male and female cohorts, grouped into four categories: "New", representing novel protein–disease associations with no existing drugs; "Druggable", where proteins are under clinical evaluation or considered viable for small-molecule development; "Repurposing", where existing drugs are approved for different diseases; and "Validated", where known drugs affect both the protein and the disease. Validated combinations are further subdivided into "Matched +", where the drug's effect direction aligns with the HDL-C estimation, and "Matched −", where the effect direction differs from the HDL-C findings. (b) Number of distinct categories per protein. (c) Representative examples of validated known targets, including drug descriptions, their primary indications or side effects, and HDL-C effect estimates. The local genetic correlation estimates are shown as solid circles with 95% confidence intervals (whiskers).

TNF superfamily member 11 (TNFSF11) displayed a risk-increasing effect on gonarthrosis (arthrosis of the knee) disease (ICD10: M17), with a significant local genetic correlation estimation of 1.00 and 95% CI (0.59, 1.00) (**Fig. 3c**). Gonarthrosis is marked by both progressive cartilage breakdown and pathologic remodeling of the subchondral bone. Increasing evidence showed that the RANK–RANKL–OPG axis is a key mediator in this process, with elevated RANKL driving osteoclast activity and contributing to aberrant bone turnover in Osteoarthritis [28,29]. Experimental studies using in vitro and animal models suggest that inhibiting RANKL can reduce excessive osteoclast-mediated resorption in the subchondral bone, potentially slowing disease progression [30,31]. Denosumab, a human monoclonal antibody targeting RANKL, effectively suppresses osteoclast formation and bone resorption and is currently approved for osteoporosis and skeletal metastases [32]. Although its use in arthrosis of the knee remains to be verified through a large-scale clinical trial, these find-

ings provide a plausible rationale for exploring RANKL inhibition as part of a disease-modifying strategy in osteoarthritis management. In summary, these findings demonstrate how integrating genetic correlation signals with drug databases can pinpoint both established and emergent therapeutic opportunities, particularly for complex disorders.

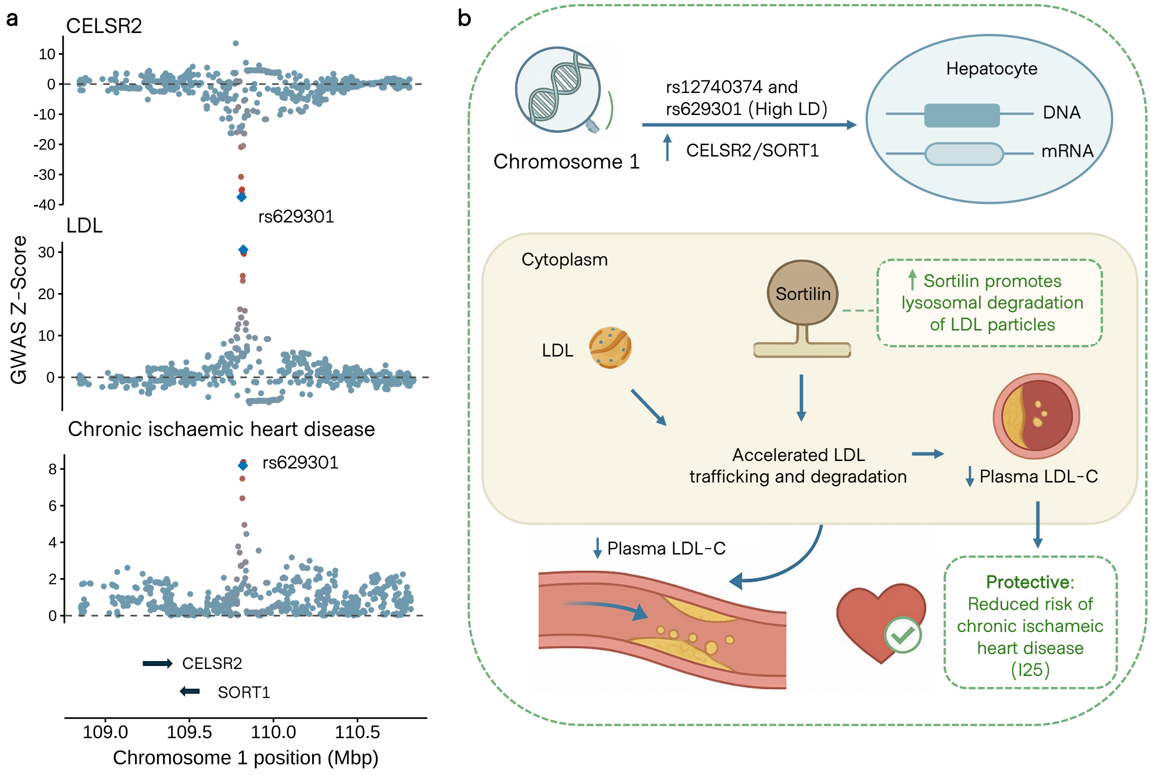

Figure 4: Potential new therapeutic target for chronic ischemic heart disease. (a) Regional association plots around CELSR2 showing: The cis-pQTL signal for plasma CELSR2 protein (top), the GWAS signal for LDL (medium), and the GWAS signal for chronic ischemic heart disease (bottom). The blue diamond shows the sentinel pQTL variant. Other variants are colored by LD to the sentinel pQTL. (b) Schematic illustration depicting the role of rs629301 in modulating CELSR2 expression and its downstream impact on LDL-C levels and ischemic heart disease (I25) risk.

Among the 63 newly identified protein–disease pairs, for instance, the HDL-C analysis found that the cadherin EGF LAG seven-pass G-type receptor 2 (CELSR2) was a potential target for chronic ischemic heart disease (ICD-10: I25). In both sexes, the local genetic correlation was nearly -1 (−0.94 in males, 95 % CI: −0.74 to −1.00; and −1.00 in females, 95 % CI: −0.62 to −1.00), driven by rs629301, a variant tightly linked to the well-studied rs12740374. The lead variant rs629301 was associated with CELSR2 protein levels in plasma, indicating a regulatory effect. This modulation likely influenced LDL-cholesterol levels and contributed to ischemic heart disease risk (**Fig. 4a**). Existing studies

indicate that rs12740374 modulates the hepatic expression of CELSR2 and its neighboring gene Sortilin 1 (SORT1), affecting LDL-cholesterol levels and coronary event risk. Functional analyses in human hepatocytes and mouse models have shown that elevated sortilin accelerates intracellular trafficking and lysosomal degradation of APOB-containing lipoproteins, reducing atherogenic particle pools [33,34] (**Fig. 4b**). These findings suggest CELSR2 as a potential therapeutic target for lipid-lowering therapies in chronic ischemic heart disease.

We further assessed whether the top-ranked HDL-C discoveries in the RDR analysis were preferentially enriched for protein–trait pairs supported by prior experimental or clinical evidence. To address this, we examined the composition of annotated categories across increasing top-N discoveries. The results showed that the enrichment for evidence-supported pairs (Matched+) was the highest among the most highly ranked signals and decreased monotonically as N increased. This indicated that the strongest, most validated signals were concentrated near the top of the ranking (Supplementary Fig. 4).

Discussion

We introduced HDL-C, a new extended approach built upon the high-definition likelihood (HDL-L) framework to infer colocalization based on sufficiently high genetic correlation. HDL-L tests for the existence of non-zero local genetic covariance. However, non-zero covariance does not necessarily imply a shared causal architecture: weak or diffuse correlations may arise from LD structure, polygenicity, or multiple distinct causal variants. In contrast, colocalization requires that the genetic effects for two traits proportionally align across the underlying causal variants, a condition that is theoretically equivalent to the local genetic correlation exceeding a meaningful threshold. Our analyses indicate that HDL-C outperforms COLOC, SuSiE, and SharePro in detecting genetic colocalization in simulated and real datasets. In the top 50 findings for male and female UK Biobank subcohorts, HDL-C not only demonstrated robust efficacy but also identified previously unrecognized genetic associations between plasma proteins and diseases. The identification of these colocalized protein-disease pairs helped understand the genetic basis of complex diseases. The results prioritized novel protein targets for further investigation, which might lead to the development of new therapeutic strategies and clinical biomarkers.

From a methodological perspective, COLOC employs summary statistics with prior probabilities to infer whether two traits share a causal variant. This method distinguishes among multiple hypotheses in a Bayesian framework, notably H3 (indicating distinct causal variants for the two traits) and H4 (indicating a shared causal variant for both traits). Although COLOC's reliance on

prior probabilities enhances flexibility, it also introduces potential bias if default priors are arbitrarily selected and not validated by sensitivity analyses. Furthermore, COLOC does not model multiple causal variants simultaneously, limiting its utility in regions of high LD, where distinguishing competing hypotheses (H3 vs H4) becomes challenging. Thus, careful application and sensitivity analyses are essential to ensure robust conclusions.

SuSiE enhances colocalization inference by explicitly modeling multiple causal signals, improving accuracy over single-variant methods. However, its performance depends critically on high-quality LD estimates. Mismatches between the LD reference panel and the study population can lead to spurious signals. Additionally, selecting the parameter L (the number of allowed causal effects) requires consideration: underestimating L risks missing true signals, while overestimating L may fragment true signals or overfit noise. In our simulations, the default L setting was adopted.

HDL-C addresses these limitations by detecting sufficiently high local genetic correlation using GWAS summary statistics while accounting for LD. This approach captures associations between genetic effect vectors across traits, independent of the number of causal variants. For example, a shared causal variant with pleiotropic effects induces proportional effect estimates across SNPs in LD, producing a strong local genetic correlation (approaching ± 1.0 depending on the directionality of the effect). Conversely, unshared distinct causal variants produce low correlations, reflecting independent signals. Another intuitive advantage is that it inherently accounts for the direction and magnitude of effects, not just their existence.

Our comparison between $r_0 = 0$ and $r_0 = 0.5$ of HDL-C showed similar AUC across simulation scenarios, indicating that HDL-C is generally robust to the choice of colocalization threshold. However, under polygenic architectures with weak underlying genetic correlations, HDL-C (0) (equivalent to the unconstrained HDL-L test) would demonstrate slightly higher power. Nevertheless, HDL-C is built upon the high-definition likelihood (HDL-L) framework, with the extension aimed at addressing the specific inferential goal of detecting colocalization rather than general genetic correlation estimation. HDL-L and related approaches estimate or test the existence of non-zero genetic covariance between two traits within a region. In contrast, colocalization usually aims to detect whether two traits share the same causal architecture within a locus, i.e., whether the genetic effects at the shared causal variants *proportionally align* between the two traits. This theoretically corresponds to a sufficiently strong local genetic correlation, i.e., $|r_G|$ exceeds a threshold. HDL-C (with non-zero r_0) tests for a large $|r_G|$ threshold (rather than $h_{12} \neq 0$). This can avoid reporting situations where small but nonzero covariances are misinterpreted as evidence of colocalization.

In practice, the choice of r_0 should reflect the scientific definition of "colocalization" for the analysis at hand. In biomarker discovery, we typically require near-identical local architectures, so

we recommend a conservative default of $r_0 \in [0.5, 0.8]$. For exploratory scans where partial sharing is acceptable, even $r_0 \approx 0$ is reasonable. We suggest two data–driven options: (i) rediscovery calibration (e.g., sex-stratified or sample-split replicates) by picking r_0 that maximizes rediscovery at a certain number of discoveries; or (ii) choosing r_0 giving a desired empirical false positive rate using negative controls (trait pairs expected not to share biology or permuted/proxy regions).

We suggest that the rediscovery rate (RDR) should be a standard criterion for validating statistical discovery methods. It is a function that considers both the false-positive rate and power in both the training and validation samples [35], which measures the probability that a declared discovery reappears upon replication. Methods that suffer from inflated Type I error often produce non-replicable signals, leading to a low rediscovery rate. The RDR analysis is a useful design for evaluating methods based on real data, which incorporates realistic information and complications that simulations cannot capture. In the particular RDR analysis of this paper, for most top discoveries, HDL-C (0) actually reported more robust/replicable results than HDL-C (0.5), indicating that 1) multiple causal variants are shared between proteins and traits; and 2) the shared (replicable) local genetic correlations between males and females do not have great magnitudes. Nevertheless, this finding currently only applies to the UK Biobank cohort, and future studies may be needed to validate.

This work presents a novel perspective on colocalization analysis, especially offering a better understanding of the genetic colocalization between human plasma proteins and complex traits. The insights gained from this study are not only valuable for genetics research but also have broad implications for the fields of personalized medicine and drug development.

Methods

Theory of the HDL-C method The high-definition likelihood inference of genetic colocalization (HDL-C) builds upon the likelihood formulation of local genetic covariance under the bivariate high-definition likelihood (HDL) framework[23]. The method provides a likelihood ratio test (LRT) under a constrained null hypothesis for determining whether the local genetic correlation between two traits exceeds a biologically meaningful threshold.

For two traits with GWAS summary z-score vectors $\mathbf{z}_1$ and $\mathbf{z}_2$ measured at M SNPs in a given linkage disequilibrium (LD) block, HDL-C assumes a bivariate Gaussian model:

$$\begin{bmatrix} \mathbf{z}_1 \\ \mathbf{z}_2 \end{bmatrix} \sim \mathcal{N}\left(0, \begin{bmatrix} \Sigma_{11} & \Sigma_{12} \\ \Sigma_{12}^\top & \Sigma_{22} \end{bmatrix}\right),$$

where Σ_{ii} and Σ_{12} denote the within-trait and cross-trait covariance structures of z-scores, respectively. Specifically,

$$\Sigma_{ii} = \frac{N_i h_i^2}{M}\mathbf{L} + \mathbf{R}, \quad \Sigma_{12} = \frac{\sqrt{N_1 N_2} h_{12}}{M}\mathbf{L},$$

where N_i is the sample size of trait i, h_i^2 denotes the local SNP heritability, h_{12} the local genetic covariance, $\mathbf{R}$ the LD correlation matrix, and $\mathbf{L} = \mathbf{R}^2$ the LD score matrix. This parameterization captures both sampling variance and LD-induced correlation between SNPs. The local genetic correlation is defined as

$$r_G = \frac{h_{12}}{\sqrt{h_1^2 h_2^2}},$$

bounded by $[-1, 1]$. HDL-C aims to test whether the magnitude of r_G exceeds a pre-specified colocalization threshold $r_0 \in [0, 1)$:

$$H_0 : |r_G| \leq r_0 \quad \Longleftrightarrow \quad |h_{12}| \leq r_0\sqrt{h_1^2 h_2^2}, \qquad H_A : |r_G| > r_0.$$

Let $\mathcal{L}(\theta)$ denote the full likelihood function with parameters $\theta = (h_1^2, h_2^2, h_{12})$. To construct the likelihood ratio test, we first estimate the trait-specific heritabilities via maximum likelihood $(\hat{h}_1^2, \hat{h}_2^2)$ under the HDL model, treating them as nuisance parameters. Then the profile liklihood of the genetic covariance is

$$\mathcal{L}_p(h_{12}) = \max_{h_1^2, h_2^2} \mathcal{L}(h_1^2, h_2^2, h_{12}) = \mathcal{L}(h_{12}, \hat{h}_1^2, \hat{h}_2^2).$$

The LRT statistic is given by

$$\Lambda = -2\ln\left[\frac{\sup \mathcal{L}_p(h_{12}) : |h_{12}| \leq r_0\sqrt{\hat{h}_1^2 \hat{h}_2^2}}{\sup \mathcal{L}_p(h_{12}) : |h_{12}| \leq \sqrt{h_1^2 h_2^2}}\right].$$

Because the null hypothesis imposes an inequality constraint on h_{12}, the asymptotic null distribution of Λ follows a chi-bar-square mixture[24]:

$$\Lambda \xrightarrow{H_0} \tfrac{1}{2}\chi_0^2 + \tfrac{1}{2}\chi_1^2.$$

Accordingly, a conservative p-value can be computed as $p = \frac{1}{2}\ \Pr(\chi_1^2 \geq \Lambda)$.

This formulation directly tests whether the estimated local genetic correlation exceeds r_0, rather than testing for zero correlation. Inference is equivalent to checking whether the likelihood-based confidence interval for r_G lies entirely outside the interval $[-r_0, r_0]$. In our analyses, we consider

two settings of the HDL-C method (under $r_0 = 0$ and $r_0 = 0.5$), where HDL-C (0) corresponds to the standard local genetic correlation testing method HDL-L.

Compared with standard Bayesian colocalization methods such as COLOC[14] or SuSiE[20], which rely on variant-level causal priors and enumeration of causal configurations, HDL-C operates purely at the regional level. By exploiting the multivariate normal structure of GWAS summary z-scores and encoding LD information through $\mathbf{R}$ and $\mathbf{L}$, HDL-C provides a high-dimensional, likelihood-based inference framework that scales efficiently to genome-wide analyses using only GWAS summary statistics and an LD reference.

In practical implementation, HDL-C is profiled over h_{12} with fixed $(\hat{h}_1^2, \hat{h}_2^2)$, optimizing the log-likelihood via Newton–Raphson iteration with a convergence tolerance of 10^{-6}.

Proteins and their summary association statistics This study focused on plasma proteins from the Pharma Proteomics Project, a precompetitive biopharmaceutical consortium that characterizes the plasma proteomic profiles of 54,219 UK Biobank participants. The proteome profiling was based on the Olink Proteomics proximity extension assay (PEA) for approximately 3,000 proteins, corresponding to the Olink Explore panel. For data processing, the first step involved downloading the protein quantitative trait loci (pQTL) summary statistics. This dataset provides comprehensive insights into the genetic determinants of protein levels. Subsequently, our attention was directed towards the genetic variants on the autosomes. Specifically, we retained all overlapping SNPs located on these chromosomes, ensuring a comprehensive coverage of autosomal genetic variations. The final step in our data preparation process entailed selecting genes positioned on the autosomes. For each of these genes, we identified and delineated the corresponding cis Region, extending ± 1 Mb from the gene's physical location. This approach enabled us to precisely target genomic regions that are likely to influence the expression levels of nearby genes, thereby providing a robust foundation for our subsequent analyses of the genetic architecture of protein expression.

Summary association statistics of diseases The UK Biobank GWAS summary statistics used in this report were obtained from the second wave of results released in 2018 by Neale's group. We selected 200 ICD-10-coded diseases from the UK Biobank, each with over 1,000 recorded cases. These diseases span a broad spectrum of diagnostic categories, including malignant neoplasms (for example, breast, colon, and lung cancer), cardiovascular conditions (such as angina pectoris, chronic ischemic heart disease, and atrial fibrillation), and a variety of musculoskeletal disorders (for instance, rheumatoid arthritis, spondylosis, and arthrosis). We also included common geni-

tourinary diseases, endocrine disorders, and gastrointestinal conditions. In addition, we included several dermatological and respiratory diagnoses, as well as injuries and other frequent causes of hospital admission. By focusing on diseases with large case counts, we ensured adequate statistical power for subsequent analyses and captured a representative range of disease phenotypes in the UK Biobank cohort.

Genome-wide pQTL analysis in males and females UK Biobank genotyping and imputation (and quality control) were performed as described previously[36]. Individual protein levels (NPX) were inverse-rank normalized, including values below the limit of detection (LOD). Before the genome-wide association study (GWAS), each protein phenotype was adjusted for the following covariates, including age, age^2, UK Biobank center, UKB genetic array, the time between blood sampling and measurement, and the first 20 genetic principal components to account for population structure. Sex-stratified GWAS analyses were conducted separately in males and females using *REGSCAN*[37]. Variants with minor allele frequency < 0.05 were excluded.

Simulation To evaluate the performance of our methodology in detecting colocalization between cis-pQTLs and disease traits. The simulations were conducted in two distinct settings: the first scenario involved 10% SNPs as causal, while the second scenario considered a single causal SNP. We randomly selected 300 cis-pQTL regions from the total of 2,826, ensuring that the distribution of cis-pQTL heritability (h_1^2)—computed from the top associated SNP in each region—closely matched the distribution observed across all regions. The SNP heritability of the top variant in each cis-region was calculated using the formula:

$$h^2 = \frac{Z^2}{N + Z^2},$$

where Z is the GWAS Z-score (i.e., the estimated effect divided by its standard error) and N is the sample size. This value reflects the proportion of variance in protein abundance explained by the most strongly associated SNP per region.

In the first simulation scenario, we assumed a polygenic architecture with 10% of the SNPs in each region designated as causal. The heritability of the disease trait (h_2^2) was varied over the set $\{0.001, 0.01, 0.1\}$, and the genetic correlation (r_G) between the disease and cis-pQTL traits was drawn from $\{0, 0.3, 0.5, 0.8, 1\}$. In the second scenario, we assumed a single causal variant model for the disease trait. While the cis-pQTL heritability remained as calculated from top variants, the heritability of the disease trait (h_2^2) was varied over $\{1 \times 10^{-5}, 5 \times 10^{-4}, 1 \times 10^{-4}, 1 \times 10^{-3}\}$. For this setting, we evaluated genetic correlations r_G from the set $\{0, -1, 1\}$, representing scenarios of no

correlation, perfect negative correlation, and perfect positive correlation between the protein and disease traits.

To simulate the genetic effects and phenotypic data, we followed a polygenic model. For each SNP j in the selected cis-pQTL region, the genetic effects β_{ij} were drawn from a bivariate normal distribution. The distribution was specified as:

$$\begin{pmatrix} \beta_{1j} \\ \beta_{2j} \end{pmatrix} \sim \mathcal{N}\left(\begin{pmatrix} 0 \\ 0 \end{pmatrix}, \begin{pmatrix} h_1^2/m & r_G\sqrt{h_1^2 h_2^2}/m \\ r_G\sqrt{h_1^2 h_2^2}/m & h_2^2/m \end{pmatrix} \right), \tag{1}$$

where h_1^2 and h_2^2 represent the heritability values for the cis-pQTL and disease traits, respectively, and m is the total number of causal SNPs selected in each simulation setting. The genetic effects β_{ij} were then used to model the phenotypic data.

The phenotypic data for the two traits, $\mathbf{y}_1$ (cis-pQTL) and $\mathbf{y}_2$ (disease), were generated by applying the polygenic model:

$$\mathbf{y}_i = \sum_{j=1}^{m} \mathbf{x}_{ij}\beta_{ij} + \varepsilon_i, (i = 1, 2) \tag{2}$$

where $\mathbf{x}_{ij}$ represents the genotype data for SNP j and ε_i denotes the residuals. These residuals were sampled from a multivariate normal distribution:

$$\begin{pmatrix} \varepsilon_1 \\ \varepsilon_2 \end{pmatrix} \sim \mathcal{N}\left(\begin{pmatrix} 0 \\ 0 \end{pmatrix}, \begin{pmatrix} (1-h_1^2)\mathbf{I} & 0 \\ 0 & (1-h_2^2)\mathbf{I} \end{pmatrix} \right), \tag{3}$$

This distribution ensures that the total phenotypic variance for each trait sums to 1. The phenotypic data were generated for each simulation replicate under the specified heritability and genetic correlation settings. The estimation of genetic covariance and genetic correlation between the cis-pQTL trait and the disease trait was performed using the method described in the HDL-C paper . HDL-C applied a likelihood-based framework to estimate these parameters. The likelihood ratio test (LRT) was used to assess the statistical significance of the genetic covariance, and the 95% confidence intervals for the genetic covariance were derived using the likelihood ratio approach, as detailed in the original method section. Each simulation setting was replicated 100 times to ensure robust performance.

Colocalization analysis We used the Bayesian colocalization analysis tool COLOC with the posterior probabilities testing the H4 colocalization hypothesis: testing for a single shared causal variant between the pair of traits. The tests were applied to the mapped cis-pQTL and the established

GWAS summary statistics. SuSiE is a flexible model that estimates the posterior distribution of causal effects at each genomic locus, allowing for the identification of multiple causal variants within a single region. The analysis was performed on the mapped cis-pQTL regions and the corresponding GWAS summary statistics, with SuSiE estimating the posterior inclusion probabilities (PIPs) for each SNP in the region. These PIPs were used to assess the strength of evidence for each variant being causal, with the highest PIPs suggesting the most likely causal variants within the identified loci.

Area under receiver operating characteristic curve To evaluate the diagnostic performance of HDL-C and COLOC, Receiver Operating Characteristic (ROC) curves were constructed. This involved plotting the true positive rate (sensitivity) against the false positive rate (1-specificity) at various threshold settings. The Area Under the Curve (AUC) of these ROC curves was then calculated, providing a quantitative measure of the overall diagnostic accuracy of each method. A higher AUC value indicates superior diagnostic performance. To statistically compare the AUCs derived from the two methods, we employed the `pROC` package in R. This package provides a nonparametric approach to assess the significance of the difference between the AUCs.

Drug target investigation For the top 50 protein-disease pairs identified by HDL-C in male and female cohorts, we systematically investigated available drugs targeting these proteins using the DrugBank and Drugs.com databases. It aimed to identify therapeutic opportunities by classifying the drug–protein–disease combinations into four main categories: New, druggable, re-purposing, and validated. A protein–disease pair was considered validated if an existing drug was known to affect both the protein and the disease. Validated pairs were further classified into ”Matched +” if the drug's impact on the protein and disease was consistent with the effect direction observed in HDL-C analysis, and ”Matched -” if the effect direction differed from the HDL-C estimation. If a drug targeted the protein but was approved for treating a different disease than the one identified by HDL-C, it was classified as a repurposing opportunity. This indicates potential to expand the drug's use into new therapeutic areas. Protein–disease pairs were labeled as druggable if the protein is currently under clinical evaluation or considered viable for development into small-molecule therapies, regardless of existing drug approval. If no known drugs were available for a given protein–disease pair, it was classified as ”New,” representing a novel therapeutic target for further exploration. This approach enabled the identification of potentially actionable therapeutic targets based on sex-specific HDL-C results.

Code availability

HDL-C is included in the HDL project available at https://github.com/YuyingLi-X/HDL-C. COLOC and SuSiE software are available at https://chr1swallace.github.io/coloc/. PLINK 2.0 (https://www.cog-genomics.org/plink/2.0/) was used to extract individual-level data of imputed SNPs from the UKBB. PLINK 1.9 (https://www.cog-genomics.org/plink/) and LDAK (http://dougspeed.com/ldak/) were used in LD correlation calculation and simulations. REGSCAN is available at https://genomics.ut.ee/en/tools.

Data availability

The individual-level genotype and phenotype data are available by application from the UKBB (http://www.ukbiobank.ac.uk/). The UKBB GWAS summary statistics by the Neale laboratory can be obtained from http://www.nealelab.is/uk-biobank/. The UKB-PPP proteogenomic results and summary association data are available through an interactive portal at http://ukb-ppp.gwas.eu. Source data are provided in this paper.

Acknowledgements

X.S. was in receipt of a National Natural Science Foundation of China (NSFC) grant (No. 12171495), a National Key Research and Development Program grant (No. 2022YFF1202105), and a Swedish Research Council (Vetenskapsrådet) grant (No. 2022-01309).

Author contributions

X.S. and Y.P. initiated and supervised the study. Y.L., R.Z., Z.Y., and T.L. performed the analysis. Y.L., Y.P., and X.S. contributed to method development. Y.L. and X.S. wrote the paper, and all the authors approved the final version.

Competing interests statement

The authors declare no competing financial interests.

References

[1] Uffelmann, E. *et al.* Genome-wide association studies. *Nature Reviews Methods Primers* **1**, 1–21 (2021). URL https://www.nature.com/articles/s43586-021-00056-9.

[2] Schmitt, A. D., Hu, M. & Ren, B. Genome-wide mapping and analysis of chromosome architecture. *Nature Reviews Molecular Cell Biology* **17**, 743–755 (2016). URL http://www.nature.com/articles/nrm.2016.104.

[3] Visscher, P. M. *et al.* 10 Years of GWAS Discovery: Biology, Function, and Translation. *American Journal of Human Genetics* **101**, 5–22 (2017). URL https://www.ncbi.nlm.nih.gov/pmc/articles/PMC5501872/.

[4] Folkersen, L. *et al.* Genomic and drug target evaluation of 90 cardiovascular proteins in 30,931 individuals. *Nature Metabolism* **2**, 1135–1148 (2020). URL https://www.nature.com/articles/s42255-020-00287-2. Publisher: Nature Publishing Group.

[5] Repetto, L. *et al.* The genetic landscape of neuro-related proteins in human plasma. *Nature Human Behaviour* **8**, 2222–2234 (2024). URL https://www.nature.com/articles/s41562-024-01963-z. Publisher: Nature Publishing Group.

[6] Anderson, N. L. & Anderson, N. G. The human plasma proteome: history, character, and diagnostic prospects. *Molecular & cellular proteomics: MCP* **1**, 845–867 (2002).

[7] Emilsson, V. *et al.* Co-regulatory networks of human serum proteins link genetics to disease. *Science (New York, N.Y.)* **361**, 769–773 (2018).

[8] Sun, B. B. *et al.* Genomic atlas of the human plasma proteome. *Nature* **558**, 73–79 (2018). URL https://www.nature.com/articles/s41586-018-0175-2.

[9] Suhre, K., McCarthy, M. I. & Schwenk, J. M. Genetics meets proteomics: perspectives for large population-based studies. *Nature Reviews Genetics* **22**, 19–37 (2021). URL https://www.nature.com/articles/s41576-020-0268-2.

[10] Sun, B. B. *et al.* Plasma proteomic associations with genetics and health in the UK Biobank. *Nature* **622**, 329–338 (2023). URL https://www.nature.com/articles/s41586-023-06592-6.

[11] Benson, M. D. *et al.* Genetic Architecture of the Cardiovascular Risk Proteome. *Circulation* **137**, 1158–1172 (2018).

[12] Zhernakova, D. V. *et al.* Individual variations in cardiovascular-disease-related protein levels are driven by genetics and gut microbiome. *Nature Genetics* **50**, 1524–1532 (2018).

[13] Yao, C. *et al.* Genome-wide mapping of plasma protein QTLs identifies putatively causal genes and pathways for cardiovascular disease. *Nature Communications* **9**, 3268 (2018).

[14] Giambartolomei, C. *et al.* Bayesian Test for Colocalisation between Pairs of Genetic Association Studies Using Summary Statistics. *PLOS Genetics* **10**, e1004383 (2014). URL https://journals.plos.org/plosgenetics/article?id=10.1371/journal.pgen.1004383. Publisher: Public Library of Science.

[15] Wallace, C. Eliciting priors and relaxing the single causal variant assumption in colocalisation analyses. *PLOS Genetics* **16**, e1008720 (2020). URL https://journals.plos.org/plosgenetics/article?id=10.1371/journal.pgen.1008720. Publisher: Public Library of Science.

[16] Miller, A. J. Selection of Subsets of Regression Variables. *Journal of the Royal Statistical Society. Series A (General)* **147**, 389–425 (1984). URL https://www.jstor.org/stable/2981576. Publisher: [Royal Statistical Society, Wiley].

[17] Asimit, J. L. *et al.* Stochastic search and joint fine-mapping increases accuracy and identifies previously unreported associations in immune-mediated diseases. *Nature Communications* **10**, 3216 (2019). URL https://www.nature.com/articles/s41467-019-11271-0. Publisher: Nature Publishing Group.

[18] Wang, G., Sarkar, A., Carbonetto, P. & Stephens, M. A Simple New Approach to Variable Selection in Regression, with Application to Genetic Fine Mapping. *Journal of the Royal Statistical Society Series B: Statistical Methodology* **82**, 1273–1300 (2020). URL https://academic.oup.com/jrsssb/article/82/5/1273/7056114.

[19] Zhu, X. & Stephens, M. BAYESIAN LARGE-SCALE MULTIPLE REGRESSION WITH SUMMARY STATISTICS FROM GENOME-WIDE ASSOCIATION STUDIES. *The annals of applied statistics* **11**, 1561–1592 (2017). URL https://www.ncbi.nlm.nih.gov/pmc/articles/PMC5796536/.

[20] Wallace, C. A more accurate method for colocalisation analysis allowing for multiple causal variants. *PLOS Genetics* **17**, e1009440 (2021). URL https://journals.plos.org/plosgenetics/article?id=10.1371/journal.pgen.1009440. Publisher: Public Library of Science.

[21] Zhang, W. *et al.* SharePro: an accurate and efficient genetic colocalization method accounting for multiple causal signals. *Bioinformatics* **40**, btae295 (2024). URL https://doi.org/10.1093/bioinformatics/btae295.

[22] Ning, Z., Pawitan, Y. & Shen, X. High-definition likelihood inference of genetic correlations across human complex traits. *Nature Genetics* **52**, 859–864 (2020). URL http://www.nature.com/articles/s41588-020-0653-y.

[23] Li, Y., Pawitan, Y. & Shen, X. An enhanced framework for local genetic correlation analysis. *Nature Genetics* **57**, 1053–1058 (2025). URL https://www.nature.com/articles/s41588-025-02123-3. Publisher: Nature Publishing Group.

[24] Self, S. G. & Liang, K.-Y. Asymptotic Properties of Maximum Likelihood Estimators and Likelihood Ratio Tests Under Nonstandard Conditions. *Journal of the American Statistical Association* **82**, 605–610 (1987). URL https://www.jstor.org/stable/2289471. Publisher: [American Statistical Association, Taylor & Francis, Ltd.].

[25] Ly, D., Forman, D., Ferlay, J., Brinton, L. A. & Cook, M. B. An International Comparison of Male and Female Breast Cancer Incidence Rates. *International journal of cancer. Journal international du cancer* **132**, 1918–1926 (2013). URL https://www.ncbi.nlm.nih.gov/pmc/articles/PMC3553266/.

[26] Bray, F. *et al.* Global cancer statistics 2022: GLOBOCAN estimates of incidence and mortality worldwide for 36 cancers in 185 countries. *CA: A Cancer Journal for Clinicians* **74**, 229–263 (2024). URL https://onlinelibrary.wiley.com/doi/abs/10.3322/caac.21834. _eprint: https://onlinelibrary.wiley.com/doi/pdf/10.3322/caac.21834.

[27] Karaman, M. W. *et al.* A quantitative analysis of kinase inhibitor selectivity. *Nature Biotechnology* **26**, 127–132 (2008).

[28] Lories, R. J. & Luyten, F. P. The bone–cartilage unit in osteoarthritis. *Nature Reviews Rheumatology* **7**, 43–49 (2011). URL https://www.nature.com/articles/nrrheum.2010.197. Publisher: Nature Publishing Group.

[29] Jura-Półtorak, A., Szeremeta, A., Olczyk, K., Zoń-Giebel, A. & Komosińska-Vassev, K. Bone Metabolism and RANKL/OPG Ratio in Rheumatoid Arthritis Women Treated with TNF-α Inhibitors. *Journal of Clinical Medicine* **10**, 2905 (2021). URL https://www.ncbi.nlm.nih.gov/pmc/articles/PMC8267676/.

[30] Shangguan, L., Ding, M., Wang, Y., Xu, H. & Liao, B. Denosumab ameliorates osteoarthritis by protecting cartilage against degradation and modulating subchondral bone remodeling. *Regenerative Therapy* **27**, 181–190 (2024).

[31] Nakashima, T., Wada, T. & Penninger, J. M. RANKL and RANK as novel therapeutic targets for arthritis. *Current Opinion in Rheumatology* **15**, 280–287 (2003).

[32] Lu, J. *et al.* Current comprehensive understanding of denosumab (the RANKL neutralizing antibody) in the treatment of bone metastasis of malignant tumors, including pharmacological mechanism and clinical trials. *Frontiers in Oncology* **13**, 1133828 (2023). URL https://www.ncbi.nlm.nih.gov/pmc/articles/PMC9969102/.

[33] Kjolby, M. *et al.* Sort1, encoded by the cardiovascular risk locus 1p13.3, is a regulator of hepatic lipoprotein export. *Cell Metabolism* **12**, 213–223 (2010).

[34] Musunuru, K. *et al.* From noncoding variant to phenotype via SORT1 at the 1p13 cholesterol locus. *Nature* **466**, 714–719 (2010). URL https://www.ncbi.nlm.nih.gov/pmc/articles/PMC3062476/.

[35] Ganna, A., Lee, D., Ingelsson, E. & Pawitan, Y. Rediscovery rate estimation for assessing the validation of significant findings in high-throughput studies. *Briefings in Bioinformatics* **16**, 563–575 (2015).

[36] Bycroft, C. *et al.* The UK Biobank resource with deep phenotyping and genomic data. *Nature* **562**, 203–209 (2018). URL https://www.nature.com/articles/s41586-018-0579-z. Publisher: Nature Publishing Group.

[37] Haller, T., Kals, M., Esko, T., Mägi, R. & Fischer, K. RegScan: a GWAS tool for quick estimation of allele effects on continuous traits and their combinations. *Briefings in Bioinformatics* **16**, 39–44 (2015). URL https://doi.org/10.1093/bib/bbt066.

Supplementary Figures

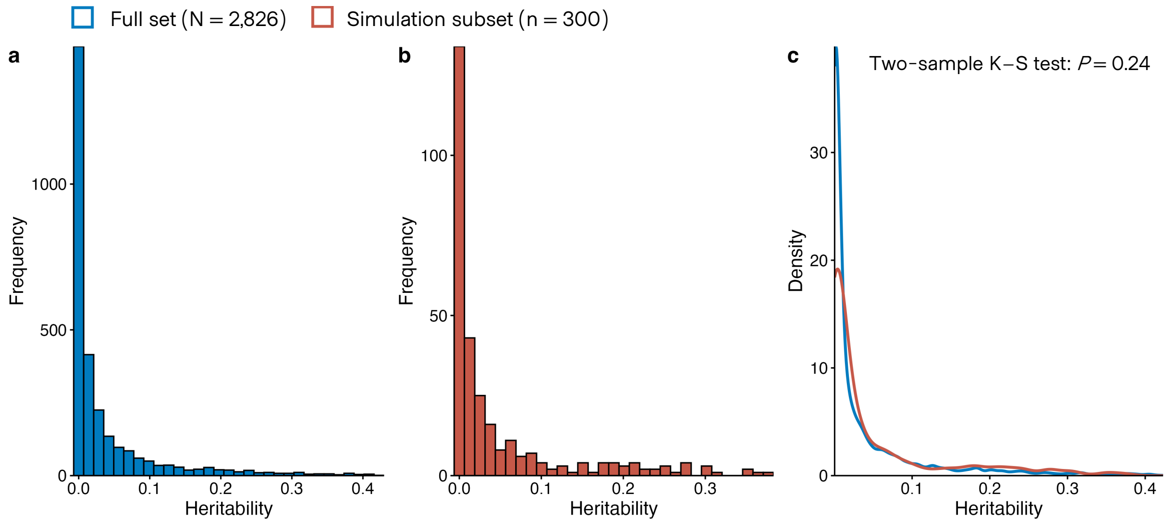

Supplementary Figure 1. Distribution of single SNP cis heritability estimates for plasma protein. The empirical distribution of the estimated heritability for the lead (top) variant in each cis pQTL region. (a) Histogram of all 2,826 cis pQTL regions. (b) Histogram of a random subset of 300 cis pQTL regions drawn without replacement so that their heritability spectrum mirrors that of the full set. (c) Kernel density overlays of the complete (blue) and subset (red) distributions. The P-value was derived by a two sample Kolmogorov–Smirnov test, confirming that no systematic bias was introduced by down sampling.

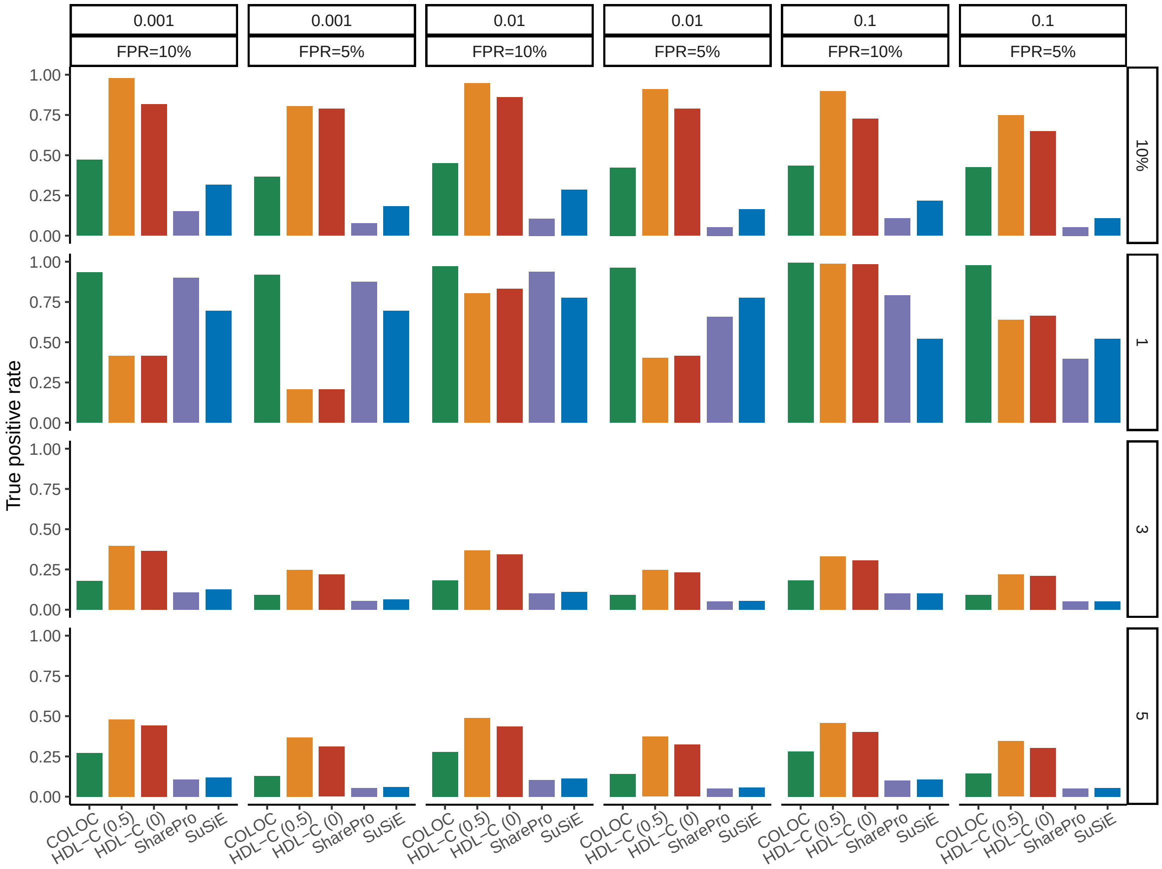

Supplementary Figure 2. True positive rate at fixed false positive rate thresholds. TPR is summarized at FPR = 5% and 10% for different numbers of causal SNPs (10%, 1, 3, 5) and heritability levels (0.001, 0.01, 0.1).

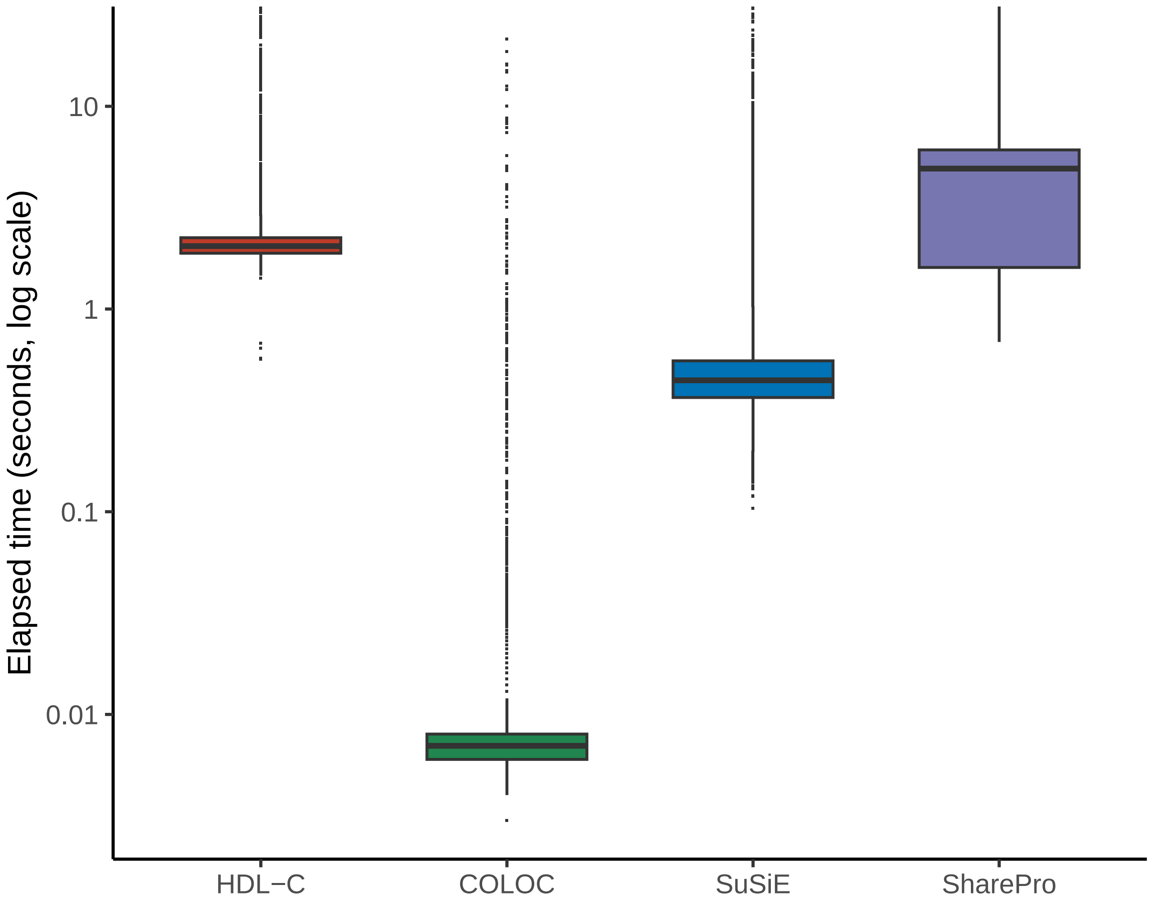

Supplementary Figure 3. Distributions of per-locus execution time of different methods. Box-plots of per-locus execution time ($\log_{10}$ seconds) across 50 simulation replicates for 300 cis-pQTL regions (i.e., $n = 15{,}000$ replicates per method). The central line marks the median and the box spans the inter-quartile range (IQR); whiskers extend to $1.5 \times$ IQR.

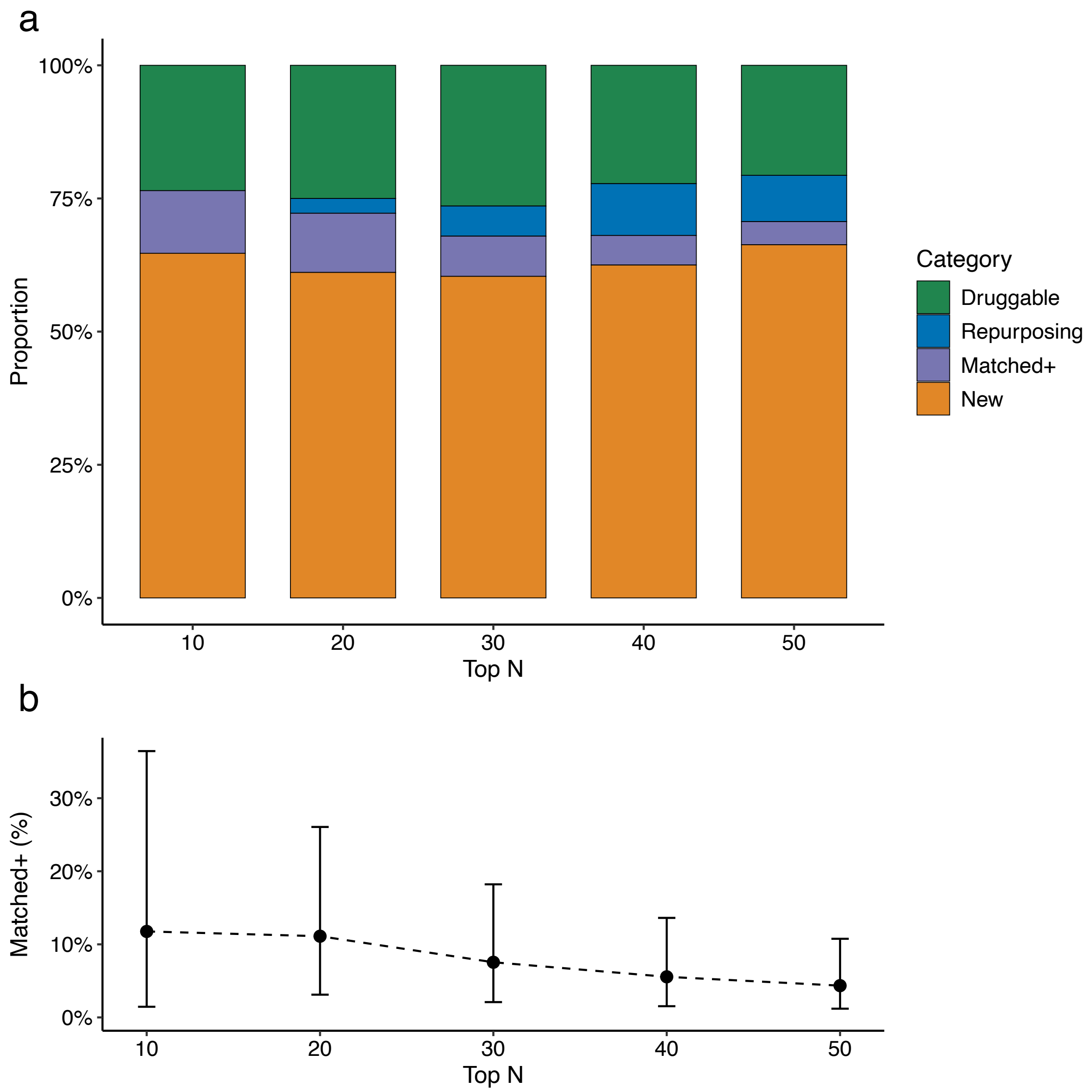

Supplementary Figure 4. Composition and enrichment across top-N thresholds. **(a)** Stacked proportions of category assignments—Druggable, Repurposing, Matched+, Matched−, and New—for the top-N HDL-C reported protein–disease pairs ($N \in \{10, 20, 30, 40, 50\}$). Proportions are computed as $Count/Total$, where $Total$ is the number of unique pairs at each N. **(b)** The fraction of Matched+ pairs is shown with 95% confidence intervals, which provide binomially valid coverage for the underlying success proportion.

Supplementary Tables

Supplementary Table 1. The AUC values of HDL-C, COLOC, SuSiE, and SharePro in simulation analyses. **Method**: Specifies the method used (HDL-C, COLOC, or SuSiE); **h11**: The simulated heritability level for the disease trait; **rg_threshold**: The threshold for the true genetic correlation used to define colocalization; **AUC**: The area under the receiver operating characteristic curve, reflecting the method's ability to distinguish between colocalized and non-colocalized regions; **CausalSNPs**: Denotes the simulation scenario—either a single causal SNP or multiple causal SNPs per region.

Supplementary Table 2. Description of 200 ICD-10 coded diseases. This table provides detailed information for 200 disease phenotypes based on ICD-10 codes used in the UK Biobank analysis. **phenotype**: ICD-10 code corresponding to each disease; **description**: Full description of the disease; **variable_type**: Indicates whether the phenotype is categorical or numerical; **source**: Source of the phenotype definition (ICD-10); **n_non_missing**: Number of individuals with non-missing phenotype data; **n_missing**: Number of individuals with missing phenotype data; **n_controls**: Number of individuals without the disease; **n_cases**: Number of individuals diagnosed with the disease.

Supplementary Table 3. Description of 2,826 proteins from the UK Biobank Pharma Proteomics Project. This table summarizes annotations and summary association statistics for 2,826 plasma proteins profiled in the UK Biobank Pharma Proteomics Project (UKB-PPP), used in cis-pQTL analyses. **UKBPPP_ProteinID**: Unique identifier of the protein, formatted as
HGNC.symbol_UniProt_OID_Panel_chr.Position.
CHR, POS19, POS38: Chromosome and position of the top associated SNP in GRCh37 (POS19) and GRCh38 (POS38) coordinates; **REF, ALT**: Reference and alternative alleles for the top SNP; **rsid**: dbSNP identifier of the top SNP; **BETA, SE**: Estimated effect size and standard error from cis-pQTL association testing; **ALTFREQ**: Frequency of the alternative allele; **N**: Sample size used in the association analysis; **Z**: Z-score, computed as the ratio of BETA to SE; **h^2**: SNP heritability of the top variant in each cis-region.

Supplementary Table 4. Top 50 results of rediscovery analysis across methods and sex-specific UK Biobank populations. This Excel file contains the top 50 significant colocalization analysis results between 2,826 proteins and 200 ICD-10 coded disease traits, stratified by sex. Results were obtained using four methods: HDL-C, COLOC, SuSiE, and SharePro. Each method was applied separately to the male and female datasets from the UK Biobank. The **threshold** denotes the

training-derived significance cutoff corresponding to the 50th-ranked signal; **measuretest** provides the value of the same ranking statistic in the opposite-sex test cohort; and **rediscovered** indicates whether the locus surpasses the training threshold in the test dataset. The **Method** and **Direction** columns specify the statistical framework used (HDL-C, HDL-L, Coloc, SuSiE, or SharePro) and the direction of replication.

Supplementary Table 5. Top 50 significant protein–disease associations from HDL-C analysis and cross-referencing with DrugBank. This table summarizes results from an extended HDL-C(0) analysis in which we investigated the top 50 significant protein–disease associations separately in male and female cohorts. The combined set included 92 unique protein–disease pairs. Each entry includes local genetic correlation estimates and inference statistics for HDL-C, along with DrugBank annotations for the corresponding protein targets. The file contains four sheets: Matched, druggable,re-purposing, and new.

uniprot, **protein**: UniProt accession and gene/protein name of the cis-pQTL.

phenotype, **description.x**: ICD-10 code and disease description.

Heritability_1, **Heritability_2**: Local SNP heritability estimates for the protein and disease traits, respectively.

Genetic_Covariance, **Genetic_Correlation**: Local genetic covariance and correlation estimates from HDL-C.

Lower_bound_rg, **Upper_bound_rg**, **P**: Likelihood-based confidence interval and P-value for local genetic correlation.

Gender: Indicates whether the result is from the male or female cohort.

drugbank_id, **name**, **description.y**: DrugBank ID, compound name, and description of known compounds targeting the protein.

known_action, **gene_name**, **cellular_location**: Drug–target interaction metadata and protein localization from DrugBank.

Supplementary Table 1

Click here to access/download
Supplementary Material
Supplementary Table 1.xlsx

Supplementary Table 2

Click here to access/download
Supplementary Material
Supplementary Table 2.xlsx

Supplementary Table 3

Click here to access/download
Supplementary Material
Supplementary Table 3.xlsx

Supplementary Table 4

Click here to access/download
Supplementary Material
Supplementary Table 4.xlsx

Supplementary Table 5

Click here to access/download
Supplementary Material
Supplementary Table 5.xlsx

Point-to-point responses to reviewers

for "High-definition likelihood inference of colocalization reveals protein biomarkers for human complex diseases" (Revision)

Nov 18, 2025

Contents

1 Summary of the Revision 2

2 Responses to Reviewer #1 3

3 Responses to Reviewer #2 8

1 Summary of the Revision

We appreciate the constructive comments from both reviewers, which have helped us improve the paper. In the revision, we followed the major comments from the two reviewers, conducted a systematic series of new simulations, re-investigated the real data analyses, and conducted careful revision (not limited to the points raised by the reviewers). The main updates include:

1. **Expanded simulations.** We conducted a comprehensive set of new simulations varying the number of causal SNPs (1, 3, 5, or 10% per region), the true genetic correlation ($r_G \in \{0, 0.3, 0.5, 0.8, 1\}$), and the regional heritability of the disease trait ($h_2^2 \in \{0.001, 0.01, 0.1\}$). These analyses confirm that HDL-C remains well-calibrated and robust across a broad range of genetic architectures.

2. **Revised methodological description.** The Methods section has been rewritten to clarify that HDL-C is based on a constrained likelihood ratio test (LRT).

3. **Enhanced comparisons with established tools.** The revised simulations now include *COLOC*, *SuSiE*, and *SharePro* as benchmark methods, allowing for a more balanced comparison across both single- and multiple-causal SNP scenarios.

4. **Sensitivity analysis for colocalization threshold r_0.** We evaluated HDL-C under multiple colocalization thresholds ($r_0 = 0.3, 0.5, 0.6, 0.8$) to assess robustness.

5. **Computational benchmarking.** We compared the computational efficiency of HDL-C with other methods. HDL-C completes within seconds per region and scales efficiently to thousands of loci, while maintaining numerical stability.

Below are our point-to-point responses to the reviewers.

2 Responses to Reviewer #1

This manuscript presents a novel colocalization analysis method based on a conditional likelihood framework. The approach is innovative, and the authors systematically benchmark the method against several widely used colocalization methods using both simulated and real-world data. Furthermore, the study convincingly demonstrates the applicability of the proposed method in practical research settings, providing valuable evidence for drug development across multiple diseases. Overall, the manuscript is of high quality and scientific relevance, with only a few points requiring further clarification; specific comments are as below:

We thank the reviewer for the positive and encouraging evaluation of our work.

1. Line 94-95, when the condition $r_0 > 0$ and $|\hat{r}_G| > r_0$ are satisfied, the scenario of $h_{12} = 0$ does not seem to be valid, which raises some uncertainty regarding the computation of the conditional likelihood function. To improve clarity for readers, I recommend that the authors provide further explanation on how this formula is derived and applied in such cases.

We thank the reviewer for bringing this concern, and we apologize for the unclear derivation. There was a mistake in the mathematical description of the *conditional* likelihood ratio test in the first version, which was supposed to be a *profile* likelihood instead. In the revision, we have revised the **Overview of the HDL-C method** section to explicitly describe that the method is based on a likelihood-ratio test (LRT) under a constrained null hypothesis. Specifically, to assess whether the local genetic correlation exceeds a prespecified colocalization threshold $r_0 \in [0, 1)$, we constructed a likelihood-ratio test (LRT) with a constrained null hypothesis of

$$H_0: \ |r_G| \leq r_0 \quad \Longleftrightarrow \quad |h_{12}| \leq r_0\sqrt{h_1^2 h_2^2},$$

against the alternative $H_A: \ |r_G| > r_0$. Let $\mathcal{L}(\theta)$ denote the full likelihood function with parameters $\theta = (h_1^2, h_2^2, h_{12})$. Then the *profile* likelihood of the genetic covariance is $\mathcal{L}_p(h_{12}) = \max_{h_1^2, h_2^2} \mathcal{L}(\theta) = \mathcal{L}(h_{12}, \hat{h}_1^2, \hat{h}_2^2)$, where $\hat{h}_1^2$ and $\hat{h}_2^2$ are the MLEs of the heritabilities. The LRT statistic for genetic

covariance is formulated as

$$\Lambda = -2\ln\left[\frac{\sup \mathcal{L}_p(h_{12}) : |h_{12}| \le r_0\sqrt{\hat{h}_1^2\hat{h}_2^2}}{\sup \mathcal{L}_p(h_{12}) : |h_{12}| \le \sqrt{h_1^2 h_2^2}}\right].$$

Because the null hypothesis involves an inequality constraint, the asymptotic null distribution of Λ follows a chi-square mixture[1]:

$$\Lambda \xrightarrow{H_0} \tfrac{1}{2}\chi_0^2 + \tfrac{1}{2}\chi_1^2,$$

i.e., an equal mixture of a point mass at zero and a chi-squared distribution with one degree of freedom.

In practice, we profile over the genetic covariance h_{12} while fixing the local heritabilities h_1^2 and h_2^2 at their unconstrained maximum-likelihood estimates. Let $B = r_0\sqrt{\widehat{h}_1^2\,\widehat{h}_2^2}$ denote the admissible interval for h_{12} under H_0. We then maximize the log-likelihood function $\ell_p(h_{12})$ subject to $-B \le h_{12} \le B$ using a bounded L-BFGS-B optimizer with multiple random restarts and small finite-difference steps for numerical stability. This likelihood-based approach ensures accurate and computationally stable testing of colocalization thresholds within the HDL-C framework.

2. It would be helpful if the authors could further compare the robustness or power of detection of HDL-C relative to other methods in the presence of noise within a region, such as when some SNPs exhibit heterogeneity.

We agree that robustness to regional noise and allelic heterogeneity is critical for evaluating colocalization methods. In the revision, we have expanded our simulation study, varying the number of causal SNPs to 1, 3, 5, or 10% (roughly between 30 and 80) within a region, the true genetic correlation $r_G \in \{0, 0.3, 0.5, 0.8, 1\}$, and the regional heritability of the complex trait $h_2^2 \in \{0.001, 0.01, 0.1\}$. We randomly chose 300 cis-regulatory gene regions to conduct the simulation. This design induces both sparsity and allelic heterogeneity of the SNP effects, as multiple causal variants can have different magnitudes and directions of the effect.

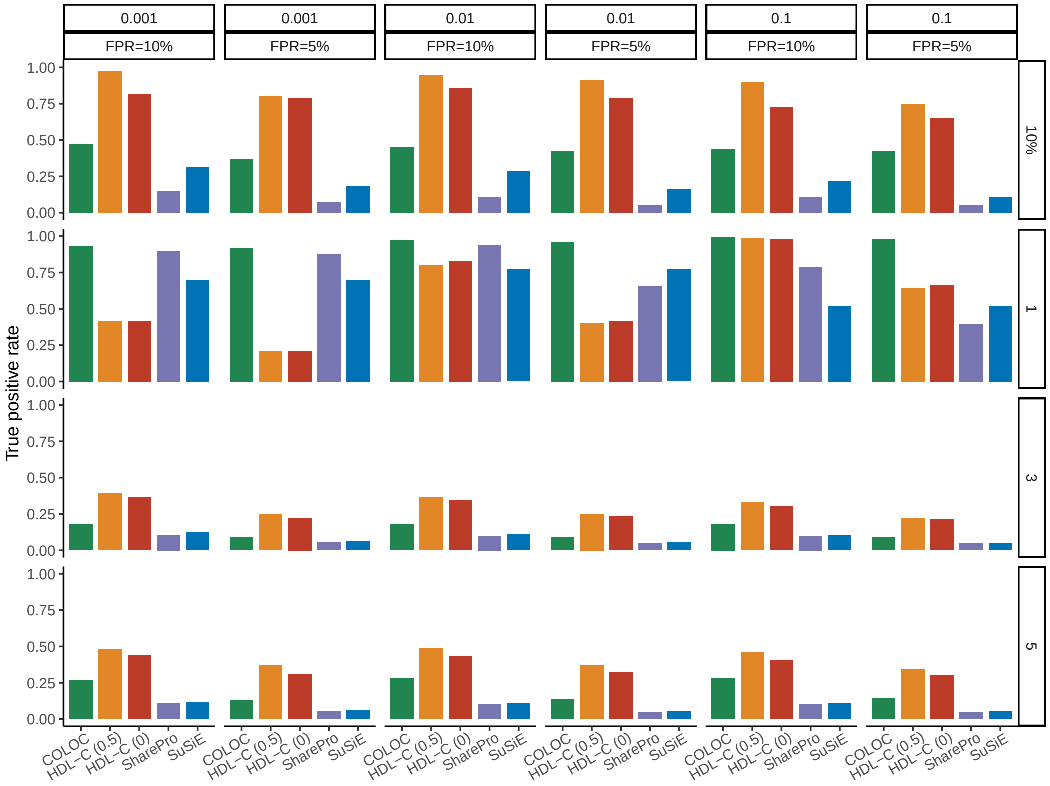

Supplementary Figure 2. True positive rate at fixed false positive rate thresholds. TPR is summarized at FPR = 5% and 10% for different number of causal SNPs (10%, 1, 3, 5) and heritability levels (0.001, 0.01, 0.1).

Also considering the suggestion of Reviewer #2, we assessed the robustness and power of HDL-C, SharePro, COLOC, and SuSiE across a range of simulated genetic architectures (Figure 1 and Supplementary Figure 2). Figure 1 summarizes the area under the ROC curve (AUC) across varying numbers of causal SNPs and regional heritability levels, demonstrating that HDL-C achieves the highest AUC under multi-causal-variant architectures (i.e., with allelic heterogeneity), whereas COLOC performs the best when only a single causal SNP exists – consistent with its single-causal-variant assumption. HDL-C was evaluated under two predefined thresholds of r_0 that determine the strength of evidence for colocalization: 1) $r_0 = 0$, i.e., colocalization is defined when genetic correlation exists; 2) a more stringent criterion of $r_0 = 0.5$, i.e., colocalization is defined when the absolute genetic correlation is statistically greater than 0.5. We denoted these two settings as HDL-C (0) and HDL-C (0.5). Supplementary Figure 2 further quantifies power at

given false positive rate (FPR) levels of 5% and 10%, showing that HDL-C consistently attains the highest power under multi-causal-variant conditions. Together, these results indicate that HDL-C provides both robustness and power, maintaining sensitivity to true colocalization signals even in the presence of within-region allelic heterogeneity.

3. The authors selected the top 50 significant protein-disease pairs in their analysis. Could the authors clarify the rationale for choosing this threshold? If 50 pairs were randomly drawn from all significant results, would HDL-C still demonstrate greater consistency or robustness compared with other methods? Furthermore, would the proportion of results that can be validated in real-world settings differ from those observed when focusing only on the top 50?

First of all, we would emphasize that the primary aim of the rediscovery rate (RDR) analysis is to benchmark different methods using real pQTL and GWAS summary statistics data. Therefore, scientific discovery is the second priority to further justify the developed HDL-C method. Due to severe multiple testing, the empirical false discovery rate even for the top discoveries was very high (over 90% if based on pure mathematical computation); however, the RDR analysis showed that the top 50 protein-trait pairs had over 15% RDR, indicating a degree of true biological effects existed among them. Therefore, we went on to check the DrugBank database and found a significant number of verifiable drug targets. Certainly, the threshold of top discoveries can be more stringent. In the revision, we examined the proportion of verifiable targets with other more stringent thresholds. As shown in Supplementary Figure 4, the enrichment for evidence-supported pairs (Matched+) is highest among the most highly ranked signals and decreases monotonically as N increases from 10 to 50. This indicates that the strongest, most validated signals are concentrated near the top of the ranking. From these, one can predict that a randomly selected set of 50 protein-trait pairs would not show such enrichment. We explained this inferential procedure in the revised Results (lines 257-263).

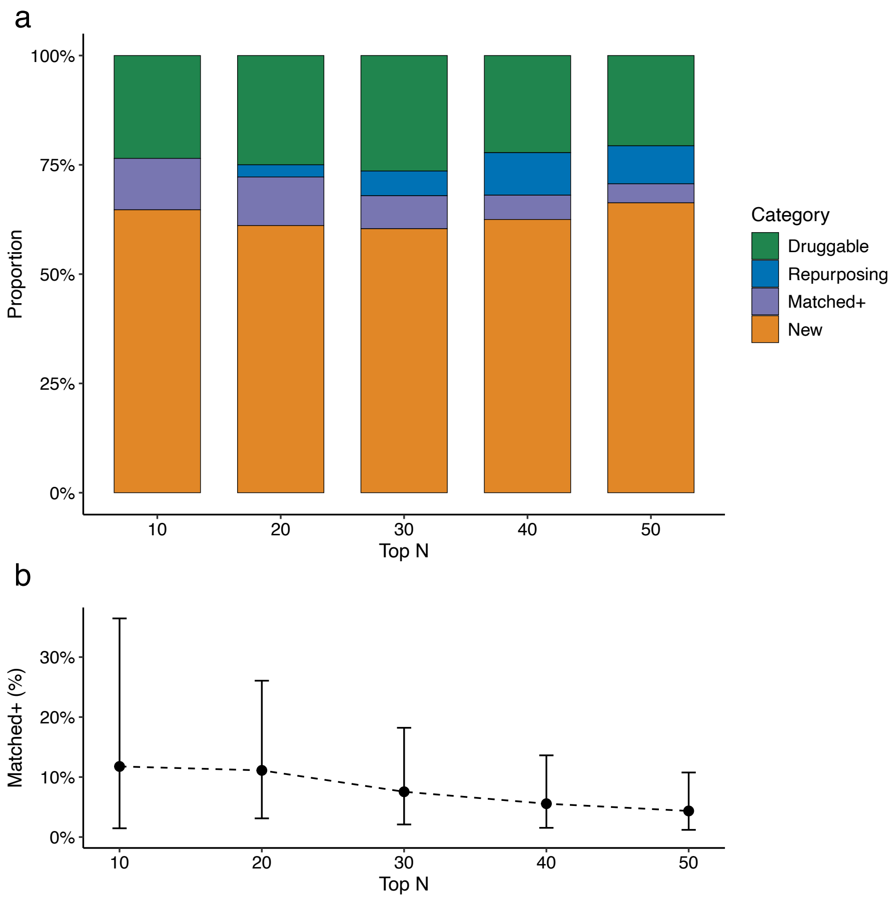

Supplementary Figure 4. Composition and enrichment across top-N thresholds. (a) Stacked proportions of category assignments (druggable, repurposing, matched+, matched−, and new) for the top-N HDL-C reported protein–disease pairs ($N \in \{10, 20, 30, 40, 50\}$). Proportions are computed as *Count*/*Total*, where *Total* is the number of unique pairs at each N. **(b)** The fraction of Matched+ pairs is shown with 95% confidence intervals, which provide binomially valid coverage for the underlying success proportion.

3 Responses to Reviewer #2

This paper presents HDL-C (High-Definition Likelihood for Colocalization), a new method for genetic colocalization analysis that aims to identify shared genetic architecture between traits by leveraging regional genetic correlations. The authors demonstrate that HDL-C outperforms existing methods like COLOC and SuSiE in detecting genetic colocalization through both simulation studies and real data applications using UK Biobank proteomics and disease data. The study has several notable strengths. The research addresses a practically important problem in human genetics, as identifying protein biomarkers through colocalization analysis has direct implications for therapeutic target discovery. The real data analysis is particularly compelling, with the identification of 92 unique protein-disease combinations and their systematic cross-referencing with DrugBank to reveal validated drug targets, repurposing opportunities, and novel therapeutic candidates. The application to sex-stratified cohorts and the rediscovery rate validation approach provide convincing evidence of the method's robustness. The writing is clear and the biological interpretations are well-grounded. However, the simulation studies are incomplete, comparing only against COLOC and SuSiE while omitting other established colocalization methods. Additionally, some simulation results contradict theoretical expectations and require better explanation. The following are specific statements of the problems:

We thank the reviewer for the encouraging feedback on our work. Below, we reply to your specific comments, which we hope improve and clarify the paper.

1. HDL-C appears to be a direct extension of HDL-L. The validity of proposed method is based on the assumption that colocalized loci have substantial genetic correlation. It seems more straightforward if simply testing the significance of genetic covariance (e.g., with HDL-L, LAVA, or superGNOVA). Can the authors clarify the necessity to use large-genetic correlation condition and empirically compare the above methods that directly test genetic covariance?

We thank the reviewer for bringing up this point, which is actually the primary motivation of this work. We agree with the reviewer that it seems simply testing the existence of local/regional

genetic correlation (or covariance) would justify some sort of genetic colocalization between the two traits.

Indeed, HDL-C is built upon the high-definition likelihood (HDL-L) framework, with the extension aimed at addressing the specific inferential goal of detecting colocalization rather than general genetic correlation estimation. HDL-L and related approaches (e.g., LAVA, superGNOVA) estimate or test the existence of non-zero genetic covariance between two traits within a region. In contrast, colocalization aims to detect whether two traits share the same causal architecture within a locus, i.e., whether the genetic effects at the shared causal variants *proportionally align* between the two traits. This theoretically corresponds to a sufficiently strong local genetic correlation, i.e., $|r_G|$ exceeds a threshold. HDL-C tests for a large $|r_G|$ threshold (rather than $h_{12} \neq 0$). This can avoid reporting situations where small but nonzero covariances are misinterpreted as evidence of colocalization. HDL-C formalizes this through a constrained likelihood-ratio test comparing the unconstrained MLE to that under the null constraint $|r_G| \leq r_0$, where r_0 (e.g., 0.5) defines the threshold for declaring strong sharing. We include relevant discussions in the revision (lines 302-314).

In the revision, we consider two settings of the HDL-C method (under $r_0 = 0$ and $r_0 = 0.5$), where HDL-C (0) corresponds to the standard local genetic correlation testing method HDL-L. We do not include LAVA and superGNOVA in the simulations, because: 1) LAVA has recently been found to be not only a severely inflated but also an even wrong method for genetic correlation analysis (see our HDL-L paper `https://www.nature.com/articles/s41588-025-02123-3` and our further preprint `https://www.preprints.org/manuscript/202508.1096`); 2) superGNOVA has not been updated for approximately four years and is not working at present – the required Plink bfiles and partition files are not available since 2024 on their GitHub page, and the authors stated in 2021 that they didn't allow the user to use their specified reference panel or LD score.

2. The simulation setting with multiple causal SNPs scenario is unrealistic and unfair. Using 10% SNPs as causal could favor the correlation-based method. The number of causal cis-pQTL SNPs is usually less than 5 in practice.

We thank the reviewer for bringing this point to our attention. We have performed additional

simulations in our revised manuscript. Specifically, we included scenarios with 1, 3, and 5 causal SNPs per region, in addition to the 10% causal-SNP setting (Figure 1, Supplementary Figure 2).

When there is a single causal SNP, COLOC achieves the highest power, consistent with its single-variant assumption. However, as the genetic architecture becomes more polygenic, with multiple causal variants within a region, HDL-C and SharePro consistently outperform COLOC and SuSiE across all the settings. In contrast, COLOC's performance declines sharply in these scenarios, reflecting its sensitivity to violations of the single-causal-variant assumption. Overall, these results demonstrate that HDL-C effectively captures shared polygenic effects and retains well-calibrated inference under complex genetic architectures.

While we included the 10% causal-SNP case for completeness and robustness assessment, the main conclusions remain firm under realistic cis-pQTL architectures with one to a few causal variants per region. In addition, we would like to note here that according to another submitted work by our group (results not shown here), having more than 10 or even more than 20 independent causal effects within a cis-pQTL is not uncommon, according to conditional analysis in the UK Biobank data ($N > 50,000$). For strong pQTL signals, e.g., $P < 10^{-300}$, we saw constantly over 15 independent genome-wide significant signals ($P < 5 \times 10^{-8}$). Thus, although a pQTL signal can be driven by a few major effects, it can still be quite polygenic, or in Reviewer #1's term, allelic heterogeneous.

3. What is the r_0 for HDL-C in Figure1a? Was it set at the true genetic correlation threshold?

We apologize for the unclear description of the figure in the first version. For the original Figure 1a, r_0, i.e., the genetic correlation threshold under the null, was zero, as the full likelihood for the original HDL-L method was adopted. In the revision, we set two thresholds of r_0 for HDL-C, denoted as HDL-C (0) and HDL-C (0.5), corresponding to $r_0 = 0$ and $r_0 = 0.5$, respectively. In the revised Figure 1a (10% causal variants scenario), we compare the performance of different methods across varying definitions of true colocalization by thresholding the true genetic correlation. In Figure 1b–d (sparse causal variants architecture), we define $r_G > 0$ as true colocalization.

4. In practice, is there a guidance on how to choose r_0? Is the proposed method robust to the setting of r_0?

In HDL-C the null hypothesis is $H_0: \ |r_G| \leq r_0$, so r_0 is a user–defined minimum degree of sharing required to call colocalization. Larger r_0 makes the null more stringent (higher specificity, lower power); smaller r_0 is more permissive (higher power, greater risk of calling partial overlap).

The choice of r_0 should reflect the scientific definition of "colocalization" for the analysis at hand. In biomarker discovery, we typically require near-identical local architectures, so we recommend a conservative default of $r_0 \in [0.5, 0.8]$. For exploratory scans where partial sharing is acceptable, even $r_0 \approx 0$ is reasonable. Two data–driven options we suggest are: (i) rediscovery calibration (e.g., sex-stratified or sample-split replicates): pick r_0 that maximizes rediscovery at a certain number of discoveries; and (ii) negative controls (trait pairs expected not to share biology or permuted/proxy regions): choose r_0 giving the desired empirical FPR.

If we compare the simulation results for $r_0 = 0$ and $r_0 = 0.5$, we noticed that the performance was quite similar. According to our sets of simulations, only when the genetic architecture is somewhat polygenic, and the underlying/latent genetic correlation threshold for colocalization is low (absolute value close to zero), HDL-C (0) (i.e., the original HDL-L method) would yield slightly greater power than HDL-C (0.5). An interesting finding in the revised manuscript was from the RDR analysis: For most top discoveries, HDL-C (0) actually reported more robust/replicable results than HDL-C (0.5), indicating that 1) multiple causal variants are shared between proteins and traits; and 2) the shared (replicable) local genetic correlations between males and females do not have great magnitudes. Nevertheless, this finding currently only applies to the UK Biobank cohort, and future studies may be needed to validate. We included these discussions in the revised manuscript (lines 315-333).

5. The simulation comparison is limited to only COLOC and SuSiE. While COLOC represents the classic colocalization approach and SuSiE focuses primarily on fine-mapping, the authors should include established colocalization methods (e.g., eCAVIAR, fastENLOC, sharepro) to provide a more comprehensive evaluation. Furthermore, the results presentation focuses solely on AUC values. Readers would benefit from seeing specific true positive rates and false positive

rates at various threshold settings to better understand method performance characteristics.

Our primary goal in simulation was to evaluate how HDL-C performs relative to widely used baselines. COLOC represents the classical Bayesian colocalization model assuming a single causal variant per trait, whereas SuSiE provides a fine-mapping–based framework capable of handling multiple causal variants. These two methods therefore form complementary baselines for assessing both the single-variant and multi-variant scenarios. In response to the reviewer's comment, we have additionally incorporated **SharePro** in the revised simulations. SharePro is a recently developed, joint fine-mapping–based colocalization method that explicitly models multiple causal variants and was shown in its original publication to outperform other established approaches, including eCAVIAR, fastENLOC, and PWCoCo, in both accuracy and computational efficiency[2]. As summarized in Table 1, SharePro achieves the highest power and fastest running time among multi-causal colocalization tools, making it a representative benchmark for the modern class of multi-signal methods. We also note that **fastENLOC** shares the same single-causal-variant assumption as COLOC when applied to summary statistics. As documented by Wen et al. (2017) and Hukku et al. (2021) (https://xqwen.github.io/fastenloc/enloc_v_coloc.html), both COLOC and fastENLOC rely on nearly identical algorithms for computing colocalization probabilities under this assumption. Therefore, we think that including both would be redundant; as the state-of-the-art COLOC method sufficiently represents this class of single-causal-variant methods. We focused our additional evaluation on HDL-C versus COLOC, SuSiE, and SharePro to ensure a comprehensive yet computationally tractable comparison.

Table 1: **Comparison of established colocalization methods.**

**Method**	**Multiple causal variants**	**Signal identification**	**Posterior summary**	**Running time (s; SD)**
COLOC	X	X	Locus-level	0.1 (0.1)
COLOC+SuSiE	✓	Separate fine-mapping	Paired locus-level	14.0 (3.3)
eCAVIAR	✓	Separate fine-mapping	Variant-level	227.7 (89.3)
PWCoCo	✓	Conditional analysis	Paired locus-level	38.1 (20.5)
SharePro	✓	Joint fine-mapping	Effect group-level	4.3 (1.1)

To facilitate a clearer understanding of methods behavior, we have expanded the result presentation beyond AUC values. Specifically, we now report true positive rates (TPR) and false

positive rates (FPR) at specific thresholds (5% and 10%, Supplementary Figure 2). These complementary summaries show that HDL-C consistently achieves the highest power (TPR) at matched FPR levels, demonstrating both strong power and robust calibration across a broad range of multi-causal-variant genetic architectures.

6. In the single causal SNP scenario, SuSiE performs best while COLOC performs worst, which contradicts theoretical expectations since SuSiE is designed for multiple causal variants while COLOC assumes a single causal variant per region. This unexpected result requires detailed analysis and discussion in the main text to help readers understand the underlying reasons.

We thank the reviewer for this constructive comment, which was also an issue we investigated after submitting the original manuscript. We would like to apologize for a mistake in our original code applying COLOC, where we put the wrong user-defined sample size argument. In the revision, we double-checked our simulation process and performed additional simulations that now include scenarios with 1, 3, and 5 causal SNPs, in addition to the previous 10% causal-SNP setting. The updated results show that under every single-causal-SNP scenario, COLOC performed as expected and achieved the highest AUC (Figure 1b), consistent with its assumption of a single causal variant. SuSiE shows slightly lower performance in this specific setting. The detailed numbers are given in Table 2 below.

7. The paper lacks discussion of computational requirements and scalability compared to existing methods, which is important for practical implementation.

We agree that practical utility depends on computational cost. We therefore benchmarked per-locus execution time across 50 simulation replicates for 300 cis-pQTL regions (i.e., 15,000 cases per method; Supplementary Fig. 3). Median runtime was 0.007s for COLOC, 0.445s for SuSiE, 2.04s for HDL-C, and 4.93s for SharePro. Thus, while HDL-C is not the fastest, it completes within a few seconds per locus with a stable upper tail (95th percentile < 3s); at this rate, 1,000 loci require ∼34 minutes on a single core. COLOC's millisecond computation reflects a simpler modeling objective. We have summarized these results in the revised Results (lines 157-162).

Table 2: AUC by method for one causal SNP

Heritability h_{11}	Method	AUC
0.001	COLOC	**0.982**
	SuSiE	0.750
	SharePro	0.927
	HDL-C (0)	0.831
	HDL-C (0.5)	0.830
0.01	COLOC	**0.994**
	SuSiE	0.825
	SharePro	0.936
	HDL-C (0)	0.894
	HDL-C (0.5)	0.893
0.1	COLOC	**0.997**
	SuSiE	0.662
	SharePro	0.920
	HDL-C (0)	0.931
	HDL-C (0.5)	0.928

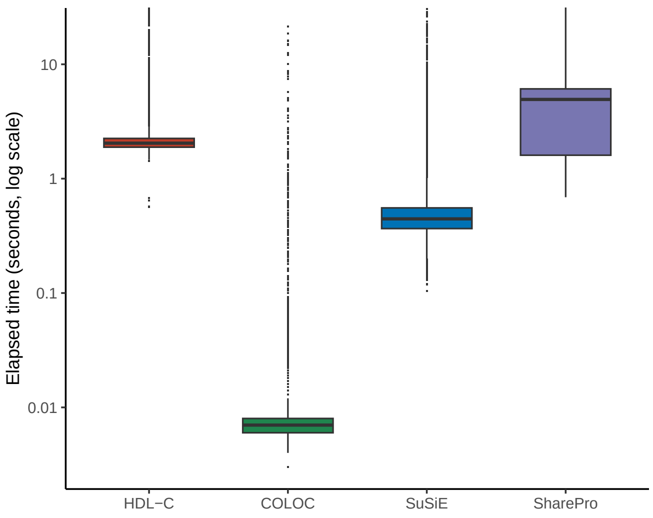

Supplementary Figure 3. Distributions of per-locus execution time of different methods. Boxplots of per-locus execution time ($\log_{10}$ seconds) across 50 simulation replicates for 300 cis-pQTL regions (i.e., $n = 15{,}000$ replicates per method). The central line marks the median and the box spans the interquartile range (IQR);

whiskers extend to 1.5 × IQR.

8. While the sex-stratified validation is interesting, the authors should clarify whether this design adequately addresses potential population stratification that might inflate apparent replication rates.

We thank the reviewer for bringing up this point. In our sex-stratified GWAS analyses, the first 20 genetic principal components (PCs) were included to account for population structure. The PCs were constructed in the entire UK Biobank cohort based on genome-wide genotypic data. Therefore, it would be unlikely that the discovered local genetic correlations were falsely generated by population structure.

Here, the sex-stratified validation was intended to assess the robustness of the discoveries from each method across two biologically distinct but (autosomal) genetically comparable subgroups. Even if false biology exists, the consistent results across sexes can still reflect the ability of a method to discover robust signals. The RDR analysis is a useful design to evaluate methods based on real data, which harbors realistic information and complications that simulations cannot capture. In the revision, we also discuss the utility of RDR analysis referring to relevant literature, trying to provide more insights to the readers (lines 322-333).

References

[1] Self, S. G. & Liang, K.-Y. Asymptotic Properties of Maximum Likelihood Estimators and Likelihood Ratio Tests Under Nonstandard Conditions. *Journal of the American Statistical Association* **82**, 605–610 (1987). URL https://www.jstor.org/stable/2289471. Publisher: [American Statistical Association, Taylor & Francis, Ltd.].

[2] Zhang, W. *et al.* SharePro: an accurate and efficient genetic colocalization method accounting for multiple causal signals. *Bioinformatics* **40**, btae295 (2024). URL https://doi.org/10.1093/bioinformatics/btae295.

High-definition likelihood inference of colocalization reveals protein biomarkers for human complex diseases

Yuying Li[1,2,†], Ranran Zhai[2,3,†],Zhijian Yang[2,5], Ting Li[2,3], Yudi Pawitan[1], Xia Shen[1,2,3,4,*]

[1]Department of Medical Epidemiology and Biostatistics, Karolinska Institutet, Stockholm, Sweden

[2]Center for Intelligent Medicine Research, Greater Bay Area Institute of Precision Medicine (Guangzhou), Fudan University, Guangzhou, China

[3]State Key Laboratory of Genetic Engineering, Center for Evolutionary Biology, School of Life Sciences, Fudan University, Shanghai, China

[4]Centre for Global Health Research, Usher Institute, University of Edinburgh, Edinburgh, UK

[5]Institute for Molecular Medicine Finland (FIMM), HiLIFE, University of Helsinki, Helsinki, Finland

[*]Correspondence should be addressed to: shenxia911@gmail.com

[†]These authors contributed equally to this work.

Abstract

Background: Genetic colocalization analysis is essential for understanding the shared genetic basis between phenotypic traits. Such an analysis is particularly useful for ~~discovering plasma proteins that have~~ identifying plasma proteins with potential as therapeutic targets or clinical biomarkers. ~~Improvement of existing tools is needed for better~~ Improvements to existing tools are needed for more accurate inference of potentially causal biomarkers.

Findings: We develop HDL-C, a high-definition likelihood inference method for genetic colocalization analysis. Based on simulations and observed rediscovery rates in real data analyses, we demonstrate that the HDL-C approach outperforms state-of-the-art methods, COLOC~~and SuSiE,~~ , SuSiE, and SharePro, in detecting genetic colocalization, thus enabling a more complete understanding of genetic connections at specific loci. Analyses of the top 50 protein−disease pairs identified by HDL-C in the male and female cohorts of the UK Biobank uncovered 40 previously validated drug-protein−disease combinations with approved drugs matching the phenotypes and 62 combinations with potential drug repurposing opportunities. Additionally, we identified 63 novel protein−disease pairs that suggest promising candidates for future therapeutic interventions.

Conclusion: This research establishes a robust framework for detecting colocalization signals, enabling the prioritization of disease-relevant protein targets and informing therapeutic development strategies.

Keywords

HDL-C, Colocalization, Genetic correlation, Proteomics, Complex diseases, Therapeutic targets

Introduction

Genetic influences underlying human diseases and traits remain an important area of investigation in genomics. Genome-wide association studies (GWAS) have significantly advanced genetic research by identifying numerous genomic regions linked to various traits and disease susceptibilities [1–5]. A key aspect of this exploration is understanding how variations in the genome correlate with variations in phenotypic traits, including those associated with complex diseases. This understanding not only reveals the genetic architecture of these traits ~~,~~ but also helps to identify potential therapeutic targets and biomarkers for disease prediction and management.

Plasma proteins, given their critical roles in various biological processes and disease pathways,

serve as valuable biomarkers and therapeutic targets. The measured proteome encompasses proteins secreted or shed ~~in~~ into the blood circulation, ~~playing the main~~ which play major roles in various molecular processes and ~~mediating~~ mediate cross-tissue ~~communications~~ communication [6]. Their expression levels, often influenced by genetic variations, can provide insights into the molecular mechanisms of diseases. Recent technological advancements in high-throughput quantification of circulating proteins have led to large-scale studies of protein quantitative trait loci (pQTL) [7–13]. These studies have highlighted the potential of associating protein levels with DNA sequence variants that colocalize with risk alleles for common diseases. Such colocalizations can reveal disease-associated pathways, offering novel insights into drug targets and translational biomarkers.

Therefore, methods for ~~better~~ more accurate inference of genetic colocalization are essential ~~in~~ for the joint analysis of molecular traits and complex diseases. COLOC is one of the most widely used methods for colocalization analysis, which aims to detect genetic colocalization between pairs of traits, such as the analysis of complex traits at specific molecular quantitative trait loci (QTL) [14]. This Bayesian model makes restrictive assumptions about the underlying shared genetic architecture at the given locus, e.g., one causal variant per trait. Such an assumption may not always hold in real-world datasets. While extensions of COLOC [15] using conditional regression have been attempted to address the issue of multiple variants, they rely on assumptions of independence among causal variants, which may not hold true, especially in the presence of extensive linkage disequilibrium (LD)[16,17]. The Sum of Single Effects (SuSiE) framework, integrated into the COLOC package, has addressed these limitations by enabling robust fine-mapping of multiple causal variants [18–20]. However, this approach primarily focuses on fine-mapping rather than quantifying colocalization itself. A more recent method is SharePro, which explicitly models multiple causal variants and joint fine-mapping [21].

As an alternative strategy, we propose the inference of a *sufficiently high* estimated regional genetic ~~correlations~~ correlation (r_G) between two phenotypes at ~~specific genomic loci~~ a specific genomic locus to detect genetic colocalization. Unlike existing methods, this strategy quantifies colocalization through a single genetic correlation parameter without strict assumptions about the underlying genetic architecture. We previously developed the high-definition likelihood (HDL) method as a robust approach for estimating genetic correlations using GWAS summary statistics[22] and the recent local version of this method, HDL-L, to estimate local genetic correlations[23]. This advancement ~~allows for a~~ enables more granular exploration of genetic correlations at specific loci. Nevertheless, for ~~the detection of colocalization~~ colocalization detection, inference must be based on a conditional likelihood given a sufficiently high regional genetic correlation estimate.

In this study, we (i) develop the theory and implement high-definition likelihood for colocaliza-

tion inference (HDL-C), (ii) demonstrate that HDL-C performs better than COLOC~~and SuSiE~~, SuSiE, and SharePro in detecting genetic colocalization, and (iii) apply the HDL-C method to investigate the colocalization between plasma proteins and complex diseases using data from the UK Biobank. Specifically, we prioritize drug targets focusing on 2,826 plasma proteins and their colocalization with 200 diseases. This approach offers a new opportunity to explore the genetic basis of disease-protein associations, potentially uncovering novel insights into disease mechanisms.

Results

Overview of the HDL-C method

~~Developing regional genetic correlation in the context of causal variant sharing, we introduce a conditional likelihood-based framework. The HDL-C method extends HDL-L by optimizing the likelihood conditioning on a high point estimate of local genetic correlation and assesses the statistical evidence that the two traits not only exhibit correlated genetic effects but are likely influenced by shared causal variants.~~ We define regional colocalization as the presence of a nonzero local genetic correlation between two traits. When the estimated correlation r_G equals zero, the local genetic effects are uncorrelated and there is no evidence of colocalization. Conversely, a significantly large r_G indicates that the traits share a consistent pattern of genetic effects within the region. In practice, one may regard a region as colocalized either when r_G is significantly different from zero, or when it exceeds a prespecified threshold $r_0 > 0$ that reflects a biologically meaningful level of correlation.

~~Let the estimated genetic correlation in a given region be $\hat{r}_G = \hat{h}_{12}/\sqrt{\hat{h}_1^2\hat{h}_2^2}$.~~ To formally test whether the local genetic correlation exceeds such a threshold, we develop the **HDL-C** method—a constrained likelihood ratio framework built upon the high-definition likelihood model to test genetic colocalization.

By definition, $r_G = h_{12}/\sqrt{h_1^2 h_2^2}$, where ~~$\hat{h}_1^2, \hat{h}_2^2, \hat{h}_{12}$, are the regional heritabilities for~~ h_1^2 and h_2^2 denote the local SNP-heritabilities of the two traits~~and their~~, and h_{12} their local genetic covariance. We consider the likelihood function $\mathcal{L}(h_1^2, h_2^2, h_{12} \mid \mathbf{z}_1, \mathbf{z}_2)$ for the regional genetic association Z-scores $(\mathbf{z}_1, \mathbf{z}_2)$, derived under the HDL multivariate normal model. We test whether the magnitude of the local genetic correlation exceeds a biologically meaningful threshold $r_0 \in [0, 1)$:

$$H_0: |r_G| \le r_0 \iff |h_{12}| \le r_0\sqrt{h_1^2 h_2^2}, \qquad H_A: |r_G| > r_0.$$

The null hypothesis therefore defines a bounded composite region in the parameter space. The *profile* likelihood of the genetic covariance~~, with $|\hat{r}_G| > r_0$ for a pre-specified threshold $r_0 \in (0, 1)$, e.~~

~~g., $r_0 = 0.5$. Under this strong correlation constraint, we test~~ is $\mathcal{L}_p(h_{12}) = \max_{h_1^2, h_2^2} \mathcal{L}(\theta) = \mathcal{L}(h_{12}, \hat{h}_1^2, \hat{h}_2^2)$, where $\hat{h}_1^2$ and $\hat{h}_2^2$ are the MLEs of the ~~hypothesis of colocalization by evaluating the significance of the estimated genetic covariance under the assumption that a strong genetic correlation implies a high probability of shared causal architecture. Let $\mathcal{L}(h_{12} \mid \mathbf{z}_1, \mathbf{z}_2, \hat{h}_1^2, \hat{h}_2^2)$ be the conditional likelihood. HDL-C constructs the conditional likelihood ratio test statistic under the constraint $(\hat{r}_G| > r_0)$ as :~~ heritabilities. The LRT statistic for genetic covariance is formulated as

$$T_{\text{HDL-C}}\Lambda = -2\log \mathcal{R}(0 \mid |_G| > r_0) = -2\log\ln \left[\frac{\mathcal{L}(h_{12} = 0 \mid \mathbf{z}_1, \mathbf{z}_2, \hat{h}_1^2, \hat{h}_2^2; |\hat{r}_G| > r_0)}{\mathcal{L}(\hat{h}_{12} \mid \mathbf{z}_1, \mathbf{z}_2, \hat{h}_1^2, \hat{h}_2^2; |\hat{r}_G| > r_0)} \frac{\sup \mathcal{L}_p(h_{12}) : |h_{12}| \le r_0 \sqrt{\hat{h}_1^2 \hat{h}_2^2}}{\sup \mathcal{L}_p(h_{12}) : |h_{12}| \le \sqrt{\hat{h}_1^2 \hat{h}_2^2}} \right];$$

~~subject to the constraint $|\hat{r}_G| > r_0$, which ensures the region is likely to harbor aligned genetic effects. We define the HDL-C p-value as:~~

$$P_{\text{HDL-C}} = \begin{cases} P\left(W \ge T_{\text{HDL-C}}\right), & \text{if } |\hat{r}_G| > r_0, \\ 1, & \text{otherwise}, \end{cases}$$

In practice, we profile over h_{12} while fixing h_1^2 and h_2^2 at their unconstrained MLEs, which preserves the null constraint $|h_{12}| \le B$, where $B = r_0\sqrt{\hat{h}_1^2 \hat{h}_2^2}$. Because the null involves an inequality constraint, the asymptotic null distribution of Λ follows a mixture of

$$\Lambda \xrightarrow{H_0} \tfrac{1}{2}\chi_0^2 + \tfrac{1}{2}\chi_1^2,$$

~~where $W \sim \chi^2(1)$ follows a chi-squared distribution with 1 degree of freedom.This formulation ensures that only regions with sufficiently strong observed genetic correlation are subject to statistical testing for colocalization (see **Methods** for the likelihood reasoning of the procedure). For these regions, a significant $p_{\text{HDL-C}}$ indicates that the observed Z-score vectors $\mathbf{z}_1, \mathbf{z}_2$ are better explained by a model with non-zero h_{12} under strong genetic correlation, suggesting a high probability of shared causal variants driving the local genetic signal in both traits~~i.e., a 50:50 mixture of a point mass at zero and a χ_1^2 distribution [24].

This procedure directly tests whether the local genetic correlation exceeds a biologically meaningful threshold r_0, rather than testing for zero correlation. It can equivalently be viewed as assessing whether the profile-likelihood confidence interval for r_G lies entirely outside the interval $[-r_0, r_0]$. In this sense, HDL-C provides a likelihood-based test of regional colocalization strength, complementing HDL-L, which estimates the magnitude of local correlation.

In contrast to standard colocalization methods, which typically model variant-level causal probabilities under strong prior assumptions, HDL-C exploits the summary-level multivariate Gaussian structure of Z-scores and the polygenic covariance encoding in the LD score matrix. HDL-C thus provides a high-dimensional, likelihood-based inference procedure for genetic colocalization ~~, requiring~~ that requires only GWAS summary statistics and an LD reference.

In practice, ~~we recommend using $r_0 = 0.5$ or higher to balance power and specificity. The resulting~~ the choice of r_0 reflects the minimum degree of local genetic sharing required to declare colocalization. In biomarker discovery, where near-identical genetic architectures are desired, we recommend a conservative range of $r_0 \in 0.5, 0.8$; for exploratory scans allowing partial sharing, $r_0 \approx 0$ is reasonable. Based on simulations, the empirical performance of HDL-C ~~p-values can be reported alongside HDL-C estimates to identify regions where strong genetic correlation co-occurs with statistically significant covariance, thereby reinforcing causal inference.~~ was generally robust across choices of r_0, with only minor power gains observed for $r_0 = 0$ under low genetic correlations. Therefore, we suggest reporting results from $r_0 = 0.5$ as a balanced default, accompanied by p-values and likelihood-based estimates of local genetic correlation to jointly assess statistical significance and biological concordance.

To evaluate the performance of HDL-C, we conducted a series of simulation studies comparing it with COLOC~~and SuSiE~~[14], SuSiE[20] and SharePro[21]. Given that COLOC inherently assumes a single causal single nucleotide polymorphism (SNP) per region, we designed simulations in two scenarios: (i) each region with a single causal SNP and (ii) each region ~~with multiple causal SNPs, where we assumed that~~ was simulated under multiple causal-variant scenarios, assuming either 3, 5, or 10% of ~~the SNPs were causal.~~SNPs as causal.. In addition, ~~for the purpose of analyzing~~ to analyze protein molecules and complex diseases, we examined different levels of true regional heritability for the disease trait. For cis-pQTLs, we estimated the heritability of the top SNP in each cis-region (see **Methods**) and then randomly selected 300 cis-pQTLs reflecting the heritability distribution of the full set of 2,826 cis-pQTLs (Supplementary Fig. 1). This subset approach was used to manage computational efficiency, as conducting simulations in all pQTL regions would be excessively computationally intensive. In each simulation replicate, we generated phenotypic data for two traits and estimated their local genetic correlation. The true effect sizes of the causal variants were drawn from a bivariate normal distribution, given the true genetic correlation (see Methods). The summary association statistics were then calculated from a genome-wide association analysis by regressing the simulated phenotypic data against the corresponding genotypes at each SNP.

HDL-C outperforms COLOC and SuSiE in detecting genetic colocalizationHDL-C outperform

Under multiple causal SNPsassumption, across varying thresholds for the absolute the assumption of 10% causal SNPs, HDL-C consistently outperformed COLOC, SuSiE, and SharePro across a range of true genetic correlation values to define true genetic colocalization (0 to 1) and true thresholds (0–1) and regional heritability levels , (**Fig. 1a**). We evaluated two HDL-C outperformed COLOC and SuSiE (**Fig. 1a**)thresholds, denoted HDL-C(0) and HDL-C(0.5), corresponding to increasingly stringent definitions of colocalization. The Area Under the Curve (AUC) of HDL-C(0) ranged from 0.92 to 0.98, compared to 0.69 to 0.93 for COLOC and 0.68 to 0.76 for SuSiE. Under one causal SNP assumption, since the with 0.82–0.99 for HDL-C(0.5), 0.70–0.93 for COLOC, 0.65–0.73 for SuSiE, and 0.53–0.65 for SharePro. More specifically, HDL-C achieved higher true positive rates (TPR) than COLOC, SuSiE, and SharePro at both 5% and 10% false positive rate (FPR) thresholds (Supplementary Fig. 2). Under the single-causal-variant setting, where true local genetic correlation would be 1 or -1 if there was colocalization , we compared the performance under different true regional heritability values (is expected to be ± 1 when colocalization exists, COLOC achieved the highest AUC, followed by SharePro, HDL-C, and SuSiE (**Fig. 1b**). When the regional heritability was relatively small, i.e., 1×10^{-5}, SuSiE performed the best. As the regional heritability increased to 0.001, these three methods achieved similar performance, with AUC values of 0.99 for SuSiEfor the disease trait increased to 0.1 in the corresponding cis-pQTL region, HDL-C performed comparably to SharePro, with AUCs of 0.93 and 0.92, respectively. In scenarios with three and five causal variants (**Fig. 1c,d**), HDL-C again delivered the strongest overall performance across all heritability levels. Notably, when the number of causal variants increased and the true genetic correlation weakened, HDL-C(0) outperformed the more conservative HDL-C(0.5). To assess computational efficiency, we benchmarked per-locus execution time across 50 simulation replicates in 300 cis-pQTL regions (15,0.98 000 runs per method; Supplementary Fig. 3). All analyses were performed on a single CPU core without parallelization, using a uniform memory allocation of 8GB for all methods. Median runtime was 0.007s for COLOC, 0.445s for SuSiE, 2.04s for HDL-C, and 0.97 for COLOC4.93s for SharePro. Although HDL-C is not the fastest, it completes within a few seconds per locus and exhibits stable upper-tail performance (95th percentile $<$ 3s). At this rate, analysis of 1,000 loci requires approximately 34 minutes on a single core. The speed of COLOC reflects its simpler single-causal-variant model.

HDL-C has higher rediscovery rates in two independent samples

To demonstrate our theory in real data analyses, we evaluated their abilities ability to detect genetic colocalization between 200 ICD-10 coded ICD-10-coded diseases and 2,826 proteins in the UK

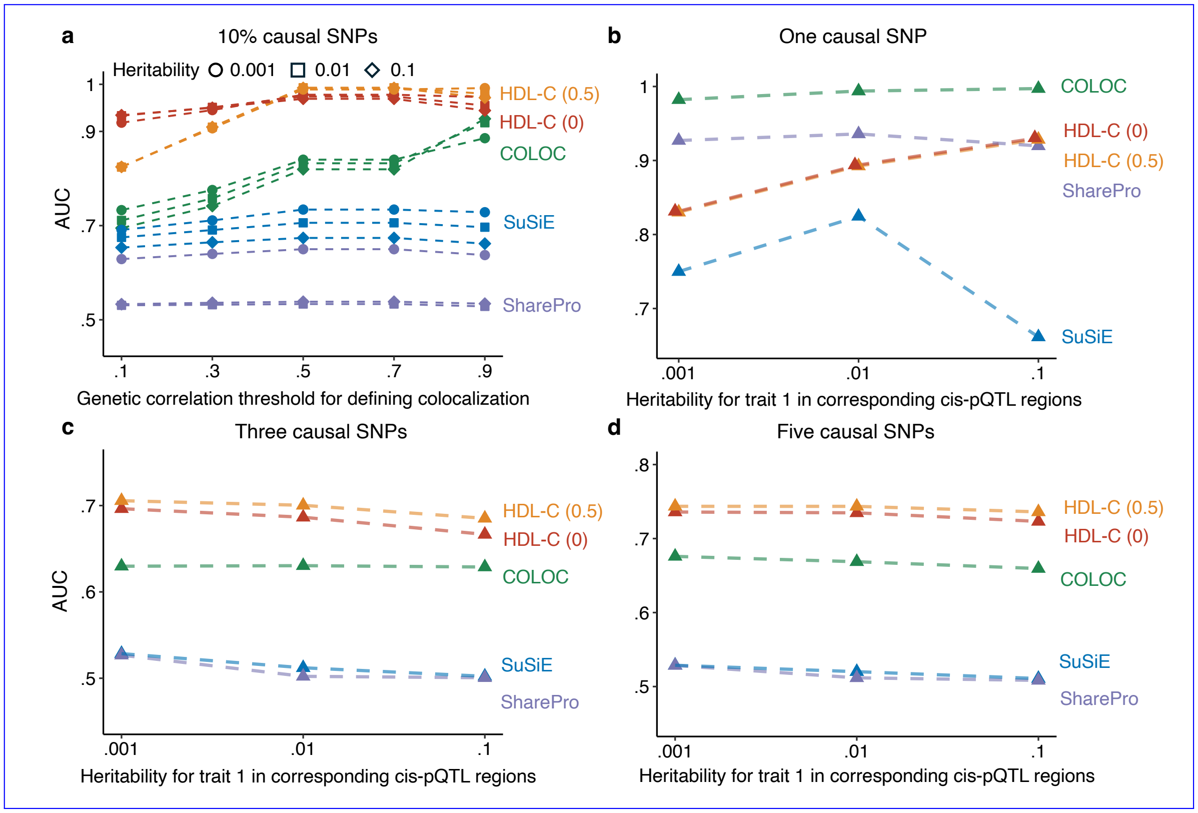

Figure 1: ~~**Performance Comparison of HDL-C, COLOC, and SuSiE in detecting genetic colocalization by AUC.**~~ ~~This figure illustrates~~ **Performance Comparison of HDL-C, COLOC, SuSiE and SharePro in detecting genetic colocalization by AUC.** Panels show the ~~performance of HDL-C, COLOC, and SuSiE in the detection of genetic colocalization~~ area under the ROC curve (AUC) for different methods across simulation settings. The colocalization level was simulated across different true genetic correlation values. (a) ~~In the multiple causal variant scenario, where~~ Results for 10% ~~of the SNPs were designated as~~ causal SNPs, plotted against the ~~x-axis represents the absolute true~~ genetic correlation ~~values~~ threshold used to define ~~genetic~~ colocalization ~~.~~ We examined three different levels of true regional heritability for disease traits corresponding to cis-pQTL regions. (~~b~~b-d) ~~In the single causal variant scenario, we randomly selected~~ Results for one~~causal SNP within each cis-pQTL region~~ , three, and ~~simulated four distinct levels of true heritability for~~ five causal SNPs, respectively. HDL-C (0) refers to the ~~disease traits~~setting in which colocalization is assessed by testing whether the local genetic correlation equals zero ($r_G = 0$), ~~confined~~ whereas HDL-C (0.5) corresponds to a more stringent criterion that tests whether the ~~same genomic region~~local genetic correlation is less than or equal to 0.5 ($r_G \leq 0.5$).

Biobank. We focused on cis-pQTL regions to explore the shared genetic architecture between these diseases and proteins in male and female populations. We used two validation settings. In the first setting, we used female data for training and male data for testing. In the second setting, we used male data for training and female data for testing. This design allowed us to directly evaluate the reproducibility of findings across sex-stratified cohorts~~directly~~. We provide detailed descriptions of the diseases and their associated proteins in Supplementary Tables 2-3. The top 50 significant colocalization results from HDL-C, COLOC, ~~and SuSiE~~SuSiE, and SharePro analyses were selected from the training set~~(Supplementary Table 4)~~, and we examined the rediscovery rates (RDR) for these methods in the test set as the proportion of overlapping results between the top 50 findings in the test and training sets (**Fig. 2**, Supplementary Table 4).

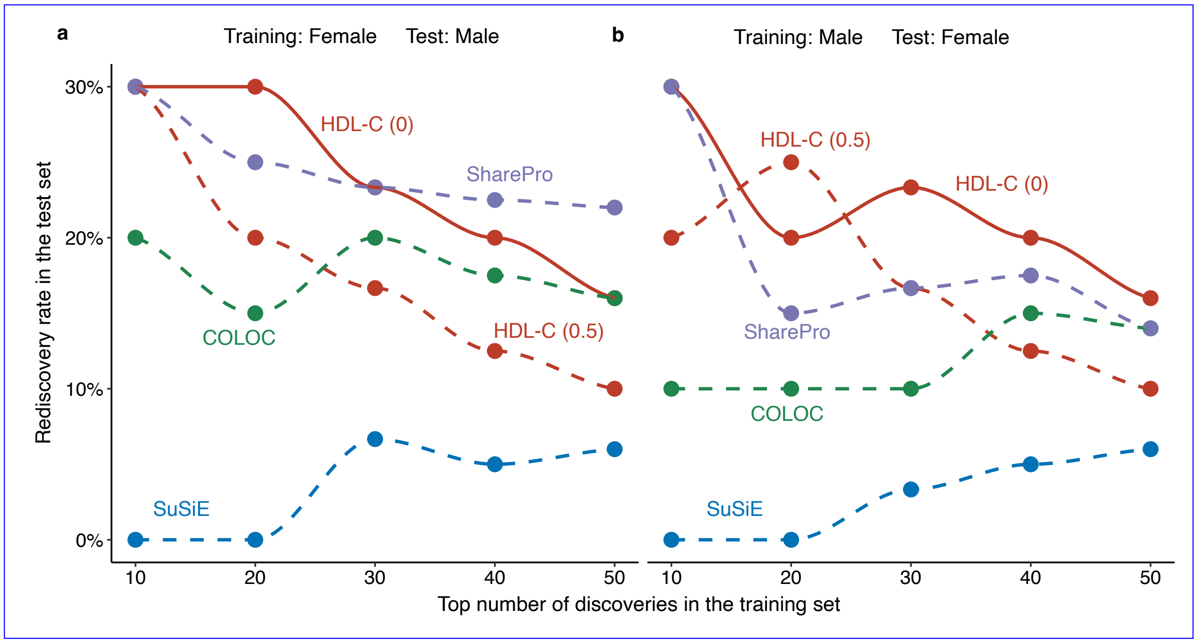

Figure 2: **~~Rediscovery rates of HDL-C, COLOC, and SuSiE under two validation settings.~~** **Rediscovery rates of HDL-C, COLOC, SuSiE, and SharePro under two validation settings.** (a) Training was performed on the female dataset and testing on the male dataset. (b) Training was performed on the male dataset and testing on the female dataset. The x-axis shows the top N significant results selected in the training set. The y-axis shows the proportion of these signals rediscovered in the test set. We applied HDL-C, COLOC, ~~and~~ SuSiE, and SharePro on ICD-10 coded 200 diseases and 2,826 proteins summary association statistics in the UK Biobank male and female population.HDL-C (0) refers to the setting in which colocalization is assessed by testing whether the local genetic correlation equals zero ($r_G = 0$), whereas HDL-C (0.5) corresponds to a more stringent criterion that tests whether the local genetic correlation is less than or equal to 0.5 ($r_G \leq 0.5$).

HDL-C~~achieved higher RDR than COLOC and SuSiE in both cross-validation scenarios. This result suggests that~~ (0) achieved the highest rediscovery rates among all evaluated methods, except in the female-to-male validation, when the top discoveries exceeded 40, where SharePro performed slightly better(**Fig. 2**). Based on simulation results, this exception likely reflects that shared causal variants between males and females are often driven by a single dominant causal SNP rather than multiple shared signals. Overall, HDL-C~~can robustly detect shared genetic signalsand adapt to sex-specific genetic variation~~(0.5) performed worse than HDL-C(0), indicating that most protein–disease associations exhibit weak and highly polygenic genetic architectures. Some diseases may have sex-specific patterns: This would influence the RDR[25,26], but not affect the comparison of performance between the methods. ~~Overall,~~ Also, in our sex-stratified GWAS analyses, the first 20 genetic principal components (PCs) were included to account for population structure. These PCs were derived from the entire UK Biobank cohort using genome-wide genotype data, making it unlikely that the observed local genetic correlations were artifacts of population stratification. Taken together, HDL-C~~showed better performance under these conditions~~(0) demonstrated superior reproducibility and robustness under these validation settings. COLOC and SuSiE could identify overlapping genetic loci, but often yielded lower RDR compared to HDL-C. SharePro achieved intermediate performance across both validation directions. This finding showed the advantage of HDL-C's likelihood-based framework in detecting robust colocalization when applied to real data.

HDL-C prioritizes drug targets for human complex diseases

We extended our analysis by investigating the top 50 genetically correlated protein–disease pairs identified by HDL-C(0) in the male and female subcohorts, respectively, resulting in 92 unique protein–disease combinations. Each of these combinations was cross-referenced with DrugBank (Supplementary Table 5). Integrating HDL-C discoveries with existing drug information, ~~there were~~ 40 validated drug-protein-disease combinations were identified where a given drug targets the same protein and treats the same disease or causes the same side effect ("Matched"). For all of the 40 matched combinations, the HDL-C inferred protein's causal effect directions were consistent with the corresponding drug action direction ("Matched $^{+}$") (**Fig. 3a**). We also identified 62 combinations where the drugs have different approved indications that differ from the diseases or side effects identified in the HDL-C results ("Re-purposing"), suggesting potential re-purposing opportunities. Furthermore, we discovered 63 protein–disease pairs where the proteins are not targeted by any drug in DrugBank ("New"), indicating potential novel therapeutic targets if the potential causal effects can be validated. In addition, we denoted 133 combinations as "Druggable", which means their ongoing evaluation in clinical trials or their viability for development into small-molecule

therapies. We further showed the distribution of these drug-protein–phenotype combinations per protein (**Fig. 3b**).

For example, we observed that Tyrosine-protein kinase Fes/Fps (FES, UniProt P07332) exhibited a protective effect against chronic ischemic heart disease (ICD10: I25), as indicated by a significant local genetic correlation estimate of −0.99 (95% CI, −0.54 to −1.00) in females and −0.81 (95% CI, −0.49 to −1.00) in males (**Fig. 3c**). Fostamatinib - marketed as Tavalisse since its FDA approval on April 17, 2018 - was developed as a spleen tyrosine kinase (SYK) inhibitor for rheumatoid arthritis and immune thrombocytopenic purpura (ITP). However, studies have demonstrated that its active metabolite (R406) can also inhibit FES, a kinase implicated in ~~protective inflammatory regulation~~ the regulation of protective inflammation [27]. While fostamatinib's anti-inflammatory properties have been investigated for mitigating vascular damage (and even acute respiratory distress syndrome in severe COVID-19), recent clinical evidence points to an increased incidence of cardiovascular side effects, notably hypertension, that may exacerbate ischemic heart conditions. This aligns with our findings of genetic correlation.

Aminocaproic acid is an antifibrinolytic agent that, by inhibiting plasminogen activation, may potentiate the prothrombotic environment in individuals with elevated apolipoprotein(a) [Lp(a)]. Higher Lp(a) levels themselves are well-documented risk factors for atherosclerotic disease, including angina pectoris, due to Lp(a)'s structural similarity to plasminogen and resultant interference with normal fibrinolysis. Thus, when aminocaproic acid further restricts fibrinolysis, it can intensify the cardiovascular risk posed by elevated Lp(a), leading to an increased incidence or severity of angina pectoris. This mechanistic interplay aligns with our significant local genetic correlation finding (0.94 with 95% CI (0.70,1.00) between Lp(a) and Angina pectoris (**Fig. 3c**), underscoring the shared risk pathway involving fibrinolysis inhibition and Lp(a)-related atherogenesis.

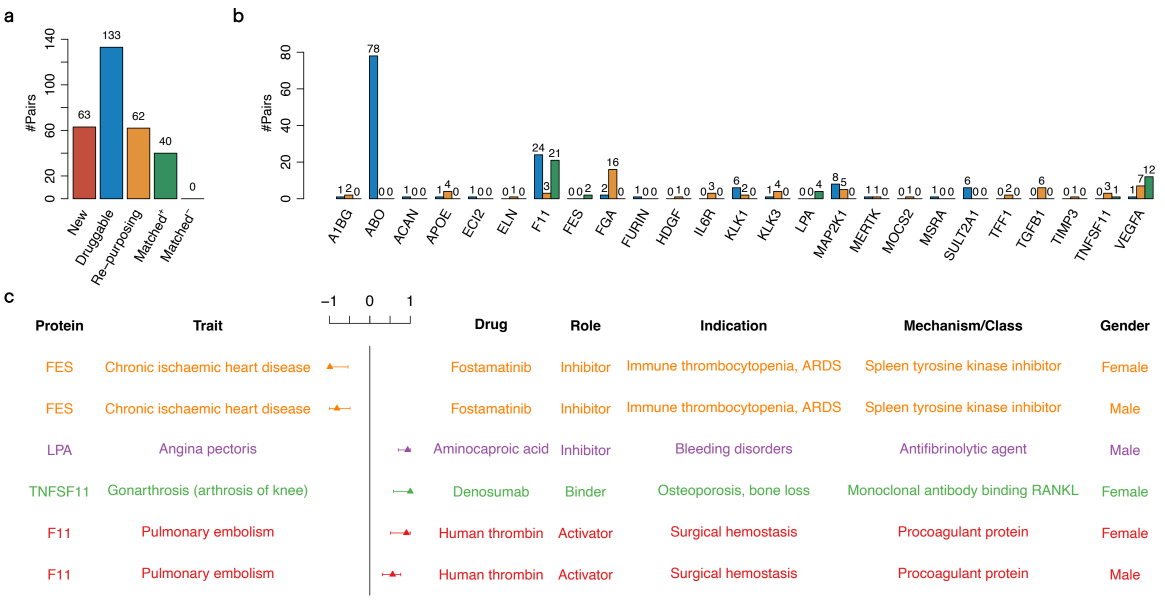

Figure 3: Drug targets inferred by local genetic correlation analysis. (a) Drug-protein–phenotype combinations identified from the top 50 HDL-C results in separate male and female cohorts, grouped into four categories: ”New”, representing novel protein–disease associations with no existing drugs; ”Druggable”, where proteins are under clinical evaluation or considered viable for small-molecule development; ”Repurposing”, where existing drugs are approved for different diseases; and “Validated”, where known drugs affect both the protein and the disease. Validated combinations are further subdivided into ”Matched +”, where the drug’s effect direction aligns with the HDL-C estimation, and ”Matched −”, where the effect direction differs from the HDL-C findings. (b) Number of distinct categories per protein. (c) Representative examples of validated known targets, including drug descriptions, their primary indications or side effects, and HDL-C effect estimates. The local genetic correlation estimates are shown as solid circles with 95% confidence intervals (whiskers).

TNF superfamily member 11 (TNFSF11) displayed a risk-increasing effect on gonarthrosis (arthrosis of the knee) disease (ICD10: M17), with a significant local genetic correlation estimation of 1.00 and 95% CI (0.59, 1.00) (**Fig. 3c**). Gonarthrosis is marked by both progressive cartilage breakdown and pathologic remodeling of the subchondral bone. Increasing evidence showed that the RANK–RANKL–OPG axis is a key mediator in this process, with elevated RANKL driving osteoclast activity and contributing to aberrant bone turnover in Osteoarthritis [28,29]. Experimental studies using in vitro and animal models suggest that inhibiting RANKL can reduce excessive osteoclast-mediated resorption in the subchondral bone, potentially slowing disease progression [30,31]. Denosumab, a human monoclonal antibody targeting RANKL, effectively suppresses osteoclast formation and bone resorption and is currently approved for osteoporosis and skeletal metastases [32]. Although its use in arthrosis of the knee remains to be verified through a large-scale clinical trial~~to verify~~,

these findings ~~illustrate~~ provide a plausible rationale for exploring RANKL inhibition as part of a disease-modifying strategy in osteoarthritis management. In summary, these findings demonstrate how integrating genetic correlation signals with drug databases can pinpoint both established and emergent therapeutic opportunities, particularly for complex disorders.

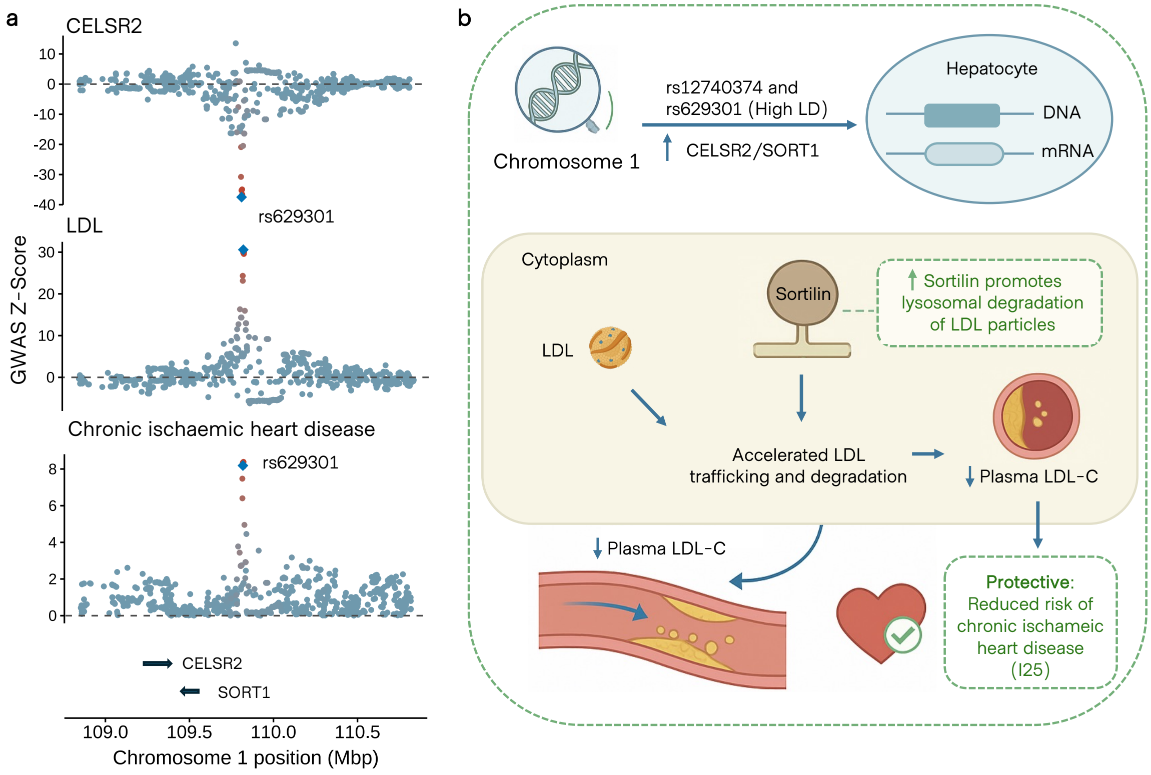

Figure 4: Potential new therapeutic target for chronic ischemic heart disease. (a) Regional association plots around CELSR2 showing: The cis-pQTL signal for plasma CELSR2 protein (top), the GWAS signal for LDL (medium), and the GWAS signal for chronic ischemic heart disease (bottom). The blue diamond shows the sentinel pQTL variant. Other variants are colored by LD to the sentinel pQTL. (b) Schematic illustration depicting the role of rs629301 in modulating CELSR2 expression and its downstream impact on LDL-C levels and ischemic heart disease (I25) risk.

Among the 63 newly identified protein–disease pairs, for instance, the HDL-C analysis found that the cadherin EGF LAG seven-pass G-type receptor 2 (CELSR2) was a potential target for chronic ischemic heart disease (ICD-10: I25). In both sexes, the local genetic correlation was nearly -1 (−0.94 in males, 95 % CI: −0.74 to −1.00; and −1.00 in females, 95 % CI: −0.62 to −1.00), driven by rs629301, a variant tightly linked to the well-studied rs12740374. The lead variant rs629301 was associated with CELSR2 protein levels in plasma, indicating a regulatory effect. This modulation likely influenced LDL-cholesterol levels and contributed to ischemic heart disease risk (**Fig. 4a**). Existing studies

indicate that rs12740374 modulates the hepatic expression of CELSR2 and its neighboring gene Sortilin 1 (SORT1), affecting LDL-cholesterol levels and coronary event risk. Functional analyses in human hepatocytes and mouse models have shown that elevated sortilin accelerates intracellular trafficking and lysosomal degradation of APOB-containing lipoproteins, reducing atherogenic particle pools [33,34] (**Fig. 4b**). These findings suggest CELSR2 as a potential therapeutic target for lipid-lowering therapies in chronic ischemic heart disease.

We further assessed whether the top-ranked HDL-C discoveries in the RDR analysis were preferentially enriched for protein–trait pairs supported by prior experimental or clinical evidence. To address this, we examined the composition of annotated categories across increasing top-*N* discoveries. The results showed that the enrichment for evidence-supported pairs (Matched+) was the highest among the most highly ranked signals and decreased monotonically as *N* increased. This indicated that the strongest, most validated signals were concentrated near the top of the ranking (Supplementary Fig. 4).

Discussion

We introduced HDL-C, a new ~~and natural approach~~ extended approach built upon the high-definition likelihood (HDL-L) framework to infer colocalization based on sufficiently high genetic correlation. ~~The~~ HDL-L tests for the existence of non-zero local genetic covariance. However, non-zero covariance does not necessarily imply a shared causal architecture: weak or diffuse correlations may arise from LD structure, polygenicity, or multiple distinct causal variants. In contrast, colocalization requires that the genetic effects for two traits proportionally align across the underlying causal variants, a condition that is theoretically equivalent to the local genetic correlation exceeding a meaningful threshold. Our analyses indicate that HDL-C outperforms ~~both COLOCand SuSiE~~COLOC, SuSiE, and SharePro in detecting genetic colocalization in simulated and real datasets. In the top 50 findings for male and female UK Biobank subcohorts, HDL-C not only demonstrated robust efficacy but also identified previously unrecognized genetic associations between plasma proteins and diseases. The identification of these colocalized protein-disease pairs helped understand the genetic basis of complex diseases. The results prioritized novel protein targets for further investigation, which might lead to the development of new therapeutic strategies and clinical biomarkers.

From a methodological perspective, COLOC employs summary statistics with prior probabilities to infer whether two traits share a causal variant. This method distinguishes among multiple hypotheses in a Bayesian framework, notably H3 (indicating distinct causal variants for the two traits) and H4 (indicating a shared causal variant for both traits). Although COLOC's reliance on

prior probabilities enhances flexibility, it also introduces potential bias if default priors are arbitrarily selected and not validated by sensitivity analyses. Furthermore, COLOC does not model multiple causal variants simultaneously, limiting its utility in regions of high LD, where distinguishing competing hypotheses (H3 vs H4) becomes challenging. Thus, careful application and sensitivity analyses are essential to ensure robust conclusions.

SuSiE enhances colocalization inference by explicitly modeling multiple causal signals, improving accuracy over single-variant methods. However, its performance depends critically on high-quality LD estimates. Mismatches between the LD reference panel and the study population can lead to spurious signals. Additionally, selecting the parameter L (the number of allowed causal effects) requires consideration: underestimating L risks missing true signals, while overestimating L may fragment true signals or overfit noise. In our simulations, the default L setting was adopted.

HDL-C addresses these limitations by detecting sufficiently high local genetic correlation using GWAS summary statistics while accounting for LD. This approach captures associations between genetic effect vectors across traits, independent of the number of causal variants. For example, a shared causal variant with pleiotropic effects induces proportional effect estimates across SNPs in LD, producing a strong local genetic correlation (approaching ± 1.0 depending on the directionality of the effect). Conversely, unshared distinct causal variants produce low correlations, reflecting independent signals. Another intuitive advantage is that it inherently accounts for the direction and magnitude of effects, not just their existence.

Our comparison between $r_0 = 0$ and $r_0 = 0.5$ of HDL-C showed similar AUC across simulation scenarios, indicating that HDL-C is generally robust to the choice of colocalization threshold. However, under polygenic architectures with weak underlying genetic correlations, HDL-C (0) (equivalent to the unconstrained HDL-L test) would demonstrate slightly higher power. Nevertheless, HDL-C is built upon the high-definition likelihood (HDL-L) framework, with the extension aimed at addressing the specific inferential goal of detecting colocalization rather than general genetic correlation estimation. HDL-L and related approaches estimate or test the existence of non-zero genetic covariance between two traits within a region. In contrast, colocalization usually aims to detect whether two traits share the same causal architecture within a locus, i.e., whether the genetic effects at the shared causal variants *proportionally align* between the two traits. This theoretically corresponds to a sufficiently strong local genetic correlation, i.e., $|r_G|$ exceeds a threshold. HDL-C (with non-zero r_0) tests for a large $|r_G|$ threshold (rather than $h_{12} \neq 0$). This can avoid reporting situations where small but nonzero covariances are misinterpreted as evidence of colocalization.

In practice, the choice of r_0 should reflect the scientific definition of "colocalization" for the analysis at hand. In biomarker discovery, we typically require near-identical local architectures, so

we recommend a conservative default of $r_0 \in [0.5, 0.8]$. For exploratory scans where partial sharing is acceptable, even $r_0 \approx 0$ is reasonable. We suggest two data–driven options: (i) rediscovery calibration (e.g., sex-stratified or sample-split replicates) by picking r_0 that maximizes rediscovery at a certain number of discoveries; or (ii) choosing r_0 giving a desired empirical false positive rate using negative controls (trait pairs expected not to share biology or permuted/proxy regions).

We suggest that the rediscovery rate (RDR) should be a standard criterion for validating statistical discovery methods. It is a function that considers both the false-positive rate and power in both the training and validation samples, which measures the probability that a declared discovery reappears upon replication. Methods that suffer from inflated Type I error often produce non-replicable signals, leading to a low rediscovery rate. The RDR analysis is a useful design for evaluating methods based on real data, which incorporates realistic information and complications that simulations cannot capture. In the particular RDR analysis of this paper, for most top discoveries, HDL-C (0) actually reported more robust/replicable results than HDL-C (0.5), indicating that 1) multiple causal variants are shared between proteins and traits; and 2) the shared (replicable) local genetic correlations between males and females do not have great magnitudes. Nevertheless, this finding currently only applies to the UK Biobank cohort, and future studies may be needed to validate.

This work presents a novel perspective on colocalization analysis, especially offering a better understanding of the genetic colocalization between human plasma proteins and complex traits. The insights gained from this study are not only valuable for genetics research but also have broad implications for the fields of personalized medicine and drug development.

Methods

Theory of the HDL-C method The high-definition likelihood inference of genetic colocalization (HDL-C) ~~is conceptually grounded in the likelihood function~~ builds upon the likelihood formulation of local genetic covariance~~, derived from GWAS summary statistics under a bivariate Gaussian model.~~ under the bivariate high-definition likelihood (HDL) framework[23]. The method provides a likelihood ratio test (LRT) under a constrained null hypothesis for determining whether the local genetic correlation between two traits exceeds a biologically meaningful threshold.

For two traits with ~~local~~ GWAS summary z-score vectors $\mathbf{z}_1$ and $\mathbf{z}_2$~~, we model their joint distribution as:~~ measured at M SNPs in a given linkage disequilibrium (LD) block, HDL-C assumes a bivariate Gaussian model:

$$\begin{bmatrix} \mathbf{z}_1 \\ \mathbf{z}_2 \end{bmatrix} \sim \mathcal{N}\left(0, \begin{bmatrix} \Sigma_{11} & \Sigma_{12} \\ \Sigma_{12}^{\top} & \Sigma_{22} \end{bmatrix}\right),$$

where ~~$\Sigma_{ii} = \frac{N_i h_i^2}{M}\mathbf{L} + \mathbf{R}$ and $\Sigma_{12} = \frac{\sqrt{N_1 N_2} h_{12}}{M}\mathbf{L}$, with~~ Σ_{ii} and Σ_{12} denote the within-trait and cross-trait covariance structures of z-scores, respectively. Specifically,

$$\Sigma_{ii} = \frac{N_i h_i^2}{M}\mathbf{L} + \mathbf{R}, \quad \Sigma_{12} = \frac{\sqrt{N_1 N_2} h_{12}}{M}\mathbf{L},$$

where N_i is the sample size of trait i, h_i^2 denotes the local SNP heritability, h_{12} ~~denoting~~ the local genetic covariance, ~~and $\mathbf{L} = \mathbf{R}^2$ and~~ $\mathbf{R}$ the LD ~~score and LD correlation matrices, respectively. Let $\mathcal{L}(h_{12} \mid \mathbf{z}, \hat{h}_1^2, \hat{h}_2^2)$ denote the conditional likelihood of the genetic covariance. The theoretical definition of~~ correlation matrix, and $\mathbf{L} = \mathbf{R}^2$ the LD score matrix. This parameterization captures both sampling variance and LD-induced correlation between SNPs. The local genetic correlation is defined as

$$r_G = \frac{h_{12}}{\sqrt{h_1^2 h_2^2}},$$

bounded by $[-1, 1]$. HDL-C ~~interprets *colocalization evidence* as the normalized likelihood mass above a user-defined genetic correlation threshold r_0, that is~~ aims to test whether the magnitude of r_G exceeds a pre-specified colocalization threshold $r_0 \in [0, 1)$:

$$H_0 : |r_G| \le r_0 \quad \Longleftrightarrow \quad |h_{12}| \le r_0 \sqrt{h_1^2 h_2^2}, \qquad H_A : |r_G| > r_0.$$

Let $\mathcal{L}(\theta)$ denote the full likelihood function with parameters $\theta = (h_1^2, h_2^2, h_{12})$. To construct the likelihood ratio test, we first estimate the trait-specific heritabilities via maximum likelihood $(\hat{h}_1^2, \hat{h}_2^2)$ under the HDL model, treating them as nuisance parameters. Then the profile liklihood of the genetic covariance is

$$\text{HDL-C score}\,\mathcal{L}_p(h_{12}) = \int_{h_{12} > r_0 \sqrt{\hat{h}_1^2 \hat{h}_2^2}} \frac{\mathcal{L}(h_{12} \mid \mathbf{z}, \hat{h}_1^2, \hat{h}_2^2)}{\int_{-\infty}^{\infty} \mathcal{L}(h_{12} \mid \mathbf{z}, \hat{h}_1^2, \hat{h}_2^2)\, \mathrm{d}h_{12}}\, \mathrm{d}\max_{h_1^2, h_2^2} \mathcal{L}(h_1^2, h_2^2, h_{12}) = \mathcal{L}(h_{12}, \hat{h}_1^2, \hat{h}_2^2).$$

~~This quantity represents the posterior weight (under uniform prior) assigned to the hypothesis that the two traits share a sufficiently large positive genetic correlation, and thus likely a shared causal signal~~The LRT statistic is given by

$$\Lambda = -2 \ln \left[\frac{\sup \mathcal{L}_p(h_{12}) : |h_{12}| \le r_0 \sqrt{\hat{h}_1^2 \hat{h}_2^2}}{\sup \mathcal{L}_p(h_{12}) : |h_{12}| \le \sqrt{h_1^2 h_2^2}} \right].$$

Because the null hypothesis imposes an inequality constraint on h_{12}, the asymptotic null distribution of Λ follows a chi-bar-square mixture[24]:

$$\Lambda \xrightarrow{H_0} \tfrac{1}{2}\chi_0^2 + \tfrac{1}{2}\chi_1^2.$$

Accordingly, a conservative p-value can be computed as $p = \frac{1}{2}\Pr(\chi_1^2 \geq \Lambda)$.

~~However, in practice, especially when the likelihood function is sharply peaked or the genetic correlation estimate $\hat{r}_G$ is large, this area-based proportion often approaches 1, providing little inferential resolution, particularly under multiple testing across genomic regions. Therefore, we propose a practical inference procedure based on the *conditional likelihood ratio test*, under the constraint $|\hat{r}_G| > r_0$. Specifically, we test:~~

$$H_0 : h_{12} = 0 \quad \text{vs.} \quad H_1 : h_{12} \neq 0, \quad \text{conditional on } |\hat{r}_G| > r_0.$$

~~This yields~~ This formulation directly tests whether the estimated local genetic correlation exceeds r_0, rather than testing for zero correlation. Inference is equivalent to checking whether the likelihood-based confidence interval for r_G lies entirely outside the interval $[-r_0, r_0]$. In our analyses, we consider two settings of the HDL-C ~~p-value:~~

$$p_{\text{HDL-C}} = \begin{cases} P\left(W \geq -2\log\frac{\mathcal{L}(0)}{\mathcal{L}(\hat{h}_{12})}\right), & \text{if } |\hat{r}_G| > r_0 \\ 1, & \text{otherwise}, \end{cases}$$

~~where $W \sim \chi^2(1)$ follows a chi-squared distribution with 1 degree of freedom,~~ method (under $r_0 = 0$ and ~~the likelihoods are evaluated conditionally on the estimated $\hat{h}_1^2$ and $\hat{h}_2^2$, as justified in Ning et al.[22].~~ $r_0 = 0.5$), where HDL-C (0) corresponds to the standard local genetic correlation testing method HDL-L.

~~This conditional framework maintains the theoretical grounding in likelihood theory while providing statistically discriminative p-values suitable for genome-wide inference, with robustness to sharp likelihood peaks and multiplicity effects. It complements and extends existing colocalization methods by incorporating both effect size magnitude and~~ Compared with standard Bayesian colocalization methods such as COLOC[14] or SuSiE[20], which rely on variant-level causal priors and enumeration of causal configurations, HDL-C operates purely at the regional level. By exploiting the multivariate normal structure of GWAS summary z-scores and encoding LD information through **R** and **L**, HDL-C provides a high-dimensional, likelihood-based ~~uncertainty.~~ inference framework that scales efficiently

to genome-wide analyses using only GWAS summary statistics and an LD reference.

In practical implementation, HDL-C is profiled over h_{12} with fixed $(\hat{h}_1^2, \hat{h}_2^2)$, optimizing the log-likelihood via Newton–Raphson iteration with a convergence tolerance of 10^{-6}.

Proteins and their summary association statistics This study focused on plasma proteins from the Pharma Proteomics Project, ~~which is~~ a precompetitive biopharmaceutical consortium ~~characterizing~~ that characterizes the plasma proteomic profiles of 54,219 UK Biobank participants. The proteome profiling was based on the Olink ~~©Proteomics ’~~ Proteomics proximity extension assay (PEA) for ~~about~~ approximately 3,000 proteins, corresponding to the Olink ~~©~~Explore panel. For data processing, the first step involved downloading the protein quantitative trait loci (pQTL) summary statistics. This dataset provides comprehensive insights into the genetic determinants of protein levels. Subsequently, our attention was directed towards the genetic variants on the autosomes. Specifically, we retained all overlapping SNPs located on these chromosomes, ensuring a comprehensive coverage of autosomal genetic variations. The final step in our data preparation process entailed selecting genes positioned on the autosomes. For each of these genes, we identified and delineated the corresponding cis Region, extending ± 1 Mb from the gene's physical location. This approach ~~allowed~~ enabled us to precisely target genomic regions that are likely to influence the expression levels of ~~the~~ nearby genes, thereby providing a robust foundation for our subsequent analyses ~~in understanding~~ of the genetic architecture of protein expression.

Summary association statistics of diseases The UK Biobank GWAS summary statistics used in this report were obtained from the second wave of results released in 2018 by Neale's group. We selected 200 ICD-10-coded diseases from the UK Biobank, each with over 1,000 recorded cases. These diseases span a broad spectrum of diagnostic categories, including malignant neoplasms (for example, breast, colon, and lung cancer), cardiovascular conditions (such as angina pectoris, chronic ischemic heart disease, and atrial fibrillation), and a variety of musculoskeletal disorders (for instance, rheumatoid arthritis, spondylosis, and arthrosis). We also included common genitourinary diseases, endocrine disorders, and gastrointestinal conditions. In addition, we included several dermatological and respiratory diagnoses, as well as injuries and other frequent causes of hospital admission. By focusing on diseases with large case counts, we ensured adequate statistical power for subsequent analyses and captured a representative range of disease phenotypes in the UK Biobank cohort.

Genome-wide pQTL analysis in males and females UK Biobank genotyping and imputation (and quality control) were performed as described previously[35]. Individual protein levels (NPX) were

inverse-rank normalized, including values below the limit of detection (LOD). Before the genome-wide association study (GWAS), each protein phenotype was adjusted for the following covariates, including age, age^2, UK Biobank center, UKB genetic array, the time between blood sampling and measurement, and the first 20 genetic principal components to account for population structure. Sex-stratified GWAS analyses were conducted separately in males and females using *REGSCAN* [36]. Variants with minor allele frequency < 0.05 were excluded.

Simulation To evaluate the performance of our methodology in detecting colocalization between cis-pQTLs and disease traits. The simulations were ~~carried out~~ conducted in two distinct settings: the first scenario involved 10% SNPs as causal, while the second scenario considered a single causal SNP. We randomly selected 300 cis-pQTL regions from the total of 2,826, ensuring that the distribution of cis-pQTL heritability (h_1^2)—computed from the top associated SNP in each region—closely matched the distribution observed across all regions. The SNP heritability of the top variant in each cis-region was calculated using the formula:

$$h^2 = \frac{Z^2}{N + Z^2},$$

where Z is the GWAS Z-score (i.e., the estimated effect divided by its standard error) and N is the sample size. This value reflects the proportion of variance in protein abundance explained by the most strongly associated SNP per region.

In the first simulation scenario, we assumed a polygenic architecture with 10% of the SNPs in each region designated as causal. The heritability of the disease trait (h_2^2) was varied over the set $\{0.001, 0.01, 0.1\}$, and the genetic correlation (r_G) between the disease and cis-pQTL traits was drawn from $\{0, 0.3, 0.5, 0.8, 1\}$. In the second scenario, we assumed a single causal variant model for the disease trait. While the cis-pQTL heritability remained as calculated from top variants, the heritability of the disease trait (h_2^2) was varied over $\{1 \times 10^{-5}, 5 \times 10^{-4}, 1 \times 10^{-4}, 1 \times 10^{-3}\}$. For this setting, we evaluated genetic correlations r_G from the set $\{0, -1, 1\}$, representing scenarios of no correlation, perfect negative correlation, and perfect positive correlation between the protein and disease traits.

To simulate the genetic effects and phenotypic data, we followed a polygenic model. For each SNP j in the selected cis-pQTL region, the genetic effects β_{ij} were drawn from a bivariate normal distribution. The distribution was specified as:

$$\begin{pmatrix} \beta_{1j} \\ \beta_{2j} \end{pmatrix} \sim \mathcal{N}\left(\begin{pmatrix} 0 \\ 0 \end{pmatrix}, \begin{pmatrix} h_1^2/m & r_G\sqrt{h_1^2 h_2^2}/m \\ r_G\sqrt{h_1^2 h_2^2}/m & h_2^2/m \end{pmatrix} \right), \tag{1}$$

where h_1^2 and h_2^2 represent the heritability values for the cis-pQTL and disease traits, respectively, and m is the total number of causal SNPs selected in each simulation setting. The genetic effects β_{ij} were then used to model the phenotypic data.

The phenotypic data for the two traits, $\mathbf{y}_1$ (cis-pQTL) and $\mathbf{y}_2$ (disease), were generated by applying the polygenic model:

$$\mathbf{y}_i = \sum_{j=1}^{m} \mathbf{x}_{ij}\beta_{ij} + \varepsilon_i, (i = 1, 2) \tag{2}$$

where $\mathbf{x}_{ij}$ represents the genotype data for SNP j and ε_i denotes the residuals. These residuals were sampled from a multivariate normal distribution:

$$\begin{pmatrix} \varepsilon_1 \\ \varepsilon_2 \end{pmatrix} \sim \mathcal{N}\left(\begin{pmatrix} 0 \\ 0 \end{pmatrix}, \begin{pmatrix} (1-h_1^2)\mathbf{I} & 0 \\ 0 & (1-h_2^2)\mathbf{I} \end{pmatrix} \right), \tag{3}$$

This distribution ensures that the total phenotypic variance for each trait sums to 1. The phenotypic data were generated for each simulation replicate under the specified heritability and genetic correlation settings. The estimation of genetic covariance and genetic correlation between the cis-pQTL trait and the disease trait was performed using the method described in the HDL-C paper . HDL-C applied a likelihood-based framework to estimate these parameters. The likelihood ratio test (LRT) was used to assess the statistical significance of the genetic covariance, and the 95% confidence intervals for the genetic covariance were derived using the likelihood ratio approach, as detailed in the original method section. Each simulation setting was replicated 100 times to ensure robust performance.

Colocalization analysis We used the Bayesian colocalization analysis tool COLOC with the posterior probabilities testing the H4 colocalization hypothesis: testing for a single shared causal variant between the pair of traits. The tests were applied to the mapped cis-pQTL and the established GWAS summary statistics. SuSiE is a flexible model that estimates the posterior distribution of causal effects at each genomic locus, allowing for the identification of multiple causal variants within a single region. The analysis was performed on the mapped cis-pQTL regions and the corresponding GWAS summary statistics, with SuSiE estimating the posterior inclusion probabilities (PIPs) for each SNP in the region. These PIPs were used to assess the strength of evidence for each variant being causal, with the highest PIPs suggesting the most likely causal variants within the identified loci.

Area under receiver operating characteristic curve To evaluate the diagnostic performance of HDL-C and COLOC, Receiver Operating Characteristic (ROC) curves were constructed. This in-

volved plotting the true positive rate (sensitivity) against the false positive rate (1-specificity) at various threshold settings. The Area Under the Curve (AUC) of these ROC curves was then calculated, providing a quantitative measure of the overall diagnostic accuracy of each method. A higher AUC value indicates superior diagnostic performance. To statistically compare the AUCs derived from the two methods, we employed the ~~pROC~~ pROC package in R. This package ~~facilitates a non-parametric approach to compute~~ provides a nonparametric approach to assess the significance of the difference between the AUCs.

Drug target investigation For the top 50 protein-disease pairs identified by HDL-C in male and female cohorts, we systematically investigated available drugs targeting these proteins using the DrugBank and Drugs.com databases. It aimed to identify therapeutic opportunities by classifying the drug–protein–disease combinations into four main categories: New, druggable, re-purposing, and validated. A protein–disease pair was considered validated if ~~there was~~ an existing drug ~~known to influence~~ was known to affect both the protein and the disease. Validated pairs were further classified into "Matched +" if the drug's impact on the protein and disease was consistent with the effect direction observed in HDL-C analysis, and "Matched -" if the effect direction differed from the HDL-C estimation. If a drug targeted the protein but was approved for treating a different disease than the one identified by HDL-C, it was classified as a repurposing opportunity. This indicates potential ~~for expanding~~ to expand the drug's use ~~to~~ into new therapeutic areas. Protein–disease pairs were labeled as druggable if the protein is currently under clinical evaluation or considered viable for development into small-molecule therapies, regardless of existing drug approval. If no known drugs were available for a given protein–disease pair, it was classified as "New," representing a novel therapeutic target for further exploration. This approach ~~allowed for~~ enabled the identification of potentially actionable therapeutic targets based on ~~the~~ sex-specific ~~results of the~~ HDL-C ~~analysis~~results.

Code availability

HDL-C is included in the HDL project available at https://github.com/YuyingLi-X/HDL-C. COLOC and SuSiE software are available at https://chr1swallace.github.io/coloc/. PLINK 2.0 (https://www.cog-genomics.org/plink/2.0/) was used to extract individual-level data of imputed SNPs from the UKBB. PLINK 1.9 (https://www.cog-genomics.org/plink/) and LDAK (http://dougspeed.com/ldak/) were used in LD correlation calculation and simulations. REGSCAN is available at https://genomics.ut.ee/en/tools.

Data availability

The individual-level genotype and phenotype data are available by application from the UKBB (http://www.ukbiobank.ac.uk/). The UKBB GWAS summary statistics by the Neale laboratory can be obtained from http://www.nealelab.is/uk-biobank/. The UKB-PPP proteogenomic results and summary association data are available through an interactive portal at http://ukb-ppp.gwas.eu. Source data are provided in this paper.

Acknowledgements

X.S. was in receipt of a National Natural Science Foundation of China (NSFC) grant (No. 12171495), a National Key Research and Development Program grant (No. 2022YFF1202105), and a Swedish Research Council (Vetenskapsrådet) grant (No. 2022-01309).

Author contributions

X.S. and Y.P. initiated and supervised the study. Y.L., R.Z., Z.Y., and T.L. performed the analysis. Y.L., Y.P., and X.S. contributed to method development. Y.L. and X.S. wrote the paper, and all the authors approved the final version.

Competing interests statement

The authors declare no competing financial interests.

References

[1] Uffelmann, E. *et al.* Genome-wide association studies. *Nature Reviews Methods Primers* **1**, 1–21 (2021). URL https://www.nature.com/articles/s43586-021-00056-9.

[2] Schmitt, A. D., Hu, M. & Ren, B. Genome-wide mapping and analysis of chromosome architecture. *Nature Reviews Molecular Cell Biology* **17**, 743–755 (2016). URL http://www.nature.com/articles/nrm.2016.104.

[3] Visscher, P. M. *et al.* 10 Years of GWAS Discovery: Biology, Function, and Translation. *American Journal of Human Genetics* **101**, 5–22 (2017). URL https://www.ncbi.nlm.nih.gov/pmc/articles/PMC5501872/.

[4] Folkersen, L. *et al.* Genomic and drug target evaluation of 90 cardiovascular proteins in 30,931 individuals. *Nature Metabolism* **2**, 1135–1148 (2020). URL https://www.nature.com/articles/s42255-020-00287-2. Publisher: Nature Publishing Group.

[5] Repetto, L. *et al.* The genetic landscape of neuro-related proteins in human plasma. *Nature Human Behaviour* **8**, 2222–2234 (2024). URL https://www.nature.com/articles/s41562-024-01963-z. Publisher: Nature Publishing Group.

[6] Anderson, N. L. & Anderson, N. G. The human plasma proteome: history, character, and diagnostic prospects. *Molecular & cellular proteomics: MCP* **1**, 845–867 (2002).

[7] Emilsson, V. *et al.* Co-regulatory networks of human serum proteins link genetics to disease. *Science (New York, N.Y.)* **361**, 769–773 (2018).

[8] Sun, B. B. *et al.* Genomic atlas of the human plasma proteome. *Nature* **558**, 73–79 (2018). URL https://www.nature.com/articles/s41586-018-0175-2.

[9] Suhre, K., McCarthy, M. I. & Schwenk, J. M. Genetics meets proteomics: perspectives for large population-based studies. *Nature Reviews Genetics* **22**, 19–37 (2021). URL https://www.nature.com/articles/s41576-020-0268-2.

[10] Sun, B. B. *et al.* Plasma proteomic associations with genetics and health in the UK Biobank. *Nature* **622**, 329–338 (2023). URL https://www.nature.com/articles/s41586-023-06592-6.

[11] Benson, M. D. *et al.* Genetic Architecture of the Cardiovascular Risk Proteome. *Circulation* **137**, 1158–1172 (2018).

[12] Zhernakova, D. V. *et al.* Individual variations in cardiovascular-disease-related protein levels are driven by genetics and gut microbiome. *Nature Genetics* **50**, 1524–1532 (2018).

[13] Yao, C. *et al.* Genome-wide mapping of plasma protein QTLs identifies putatively causal genes and pathways for cardiovascular disease. *Nature Communications* **9**, 3268 (2018).

[14] Giambartolomei, C. *et al.* Bayesian Test for Colocalisation between Pairs of Genetic Association Studies Using Summary Statistics. *PLOS Genetics* **10**, e1004383 (2014). URL https://journals.plos.org/plosgenetics/article?id=10.1371/journal.pgen.1004383. Publisher: Public Library of Science.

[15] Wallace, C. Eliciting priors and relaxing the single causal variant assumption in colocalisation analyses. *PLOS Genetics* **16**, e1008720 (2020). URL https://journals.plos.org/

plosgenetics/article?id=10.1371/journal.pgen.1008720. Publisher: Public Library of Science.

[16] Miller, A. J. Selection of Subsets of Regression Variables. *Journal of the Royal Statistical Society. Series A (General)* **147**, 389–425 (1984). URL https://www.jstor.org/stable/2981576. Publisher: [Royal Statistical Society, Wiley].

[17] Asimit, J. L. *et al.* Stochastic search and joint fine-mapping increases accuracy and identifies previously unreported associations in immune-mediated diseases. *Nature Communications* **10**, 3216 (2019). URL https://www.nature.com/articles/s41467-019-11271-0. Publisher: Nature Publishing Group.

[18] Wang, G., Sarkar, A., Carbonetto, P. & Stephens, M. A Simple New Approach to Variable Selection in Regression, with Application to Genetic Fine Mapping. *Journal of the Royal Statistical Society Series B: Statistical Methodology* **82**, 1273–1300 (2020). URL https://academic.oup.com/jrsssb/article/82/5/1273/7056114.

[19] Zhu, X. & Stephens, M. BAYESIAN LARGE-SCALE MULTIPLE REGRESSION WITH SUMMARY STATISTICS FROM GENOME-WIDE ASSOCIATION STUDIES. *The annals of applied statistics* **11**, 1561–1592 (2017). URL https://www.ncbi.nlm.nih.gov/pmc/articles/PMC5796536/.

[20] Wallace, C. A more accurate method for colocalisation analysis allowing for multiple causal variants. *PLOS Genetics* **17**, e1009440 (2021). URL https://journals.plos.org/plosgenetics/article?id=10.1371/journal.pgen.1009440. Publisher: Public Library of Science.

[21] Zhang, W. *et al.* SharePro: an accurate and efficient genetic colocalization method accounting for multiple causal signals. *Bioinformatics* **40**, btae295 (2024). URL https://doi.org/10.1093/bioinformatics/btae295.

[22] Ning, Z., Pawitan, Y. & Shen, X. High-definition likelihood inference of genetic correlations across human complex traits. *Nature Genetics* **52**, 859–864 (2020). URL http://www.nature.com/articles/s41588-020-0653-y.

[23] Li, Y., Pawitan, Y. & Shen, X. An enhanced framework for local genetic correlation analysis. *Nature Genetics* **57**, 1053–1058 (2025). URL https://www.nature.com/articles/s41588-025-02123-3. Publisher: Nature Publishing Group.

[24] Self, S. G. & Liang, K.-Y. Asymptotic Properties of Maximum Likelihood Estimators and Likelihood Ratio Tests Under Nonstandard Conditions. *Journal of the American Statistical Association* **82**, 605–610 (1987). URL https://www.jstor.org/stable/2289471. Publisher: [American Statistical Association, Taylor & Francis, Ltd.].

[25] Ly, D., Forman, D., Ferlay, J., Brinton, L. A. & Cook, M. B. An International Comparison of Male and Female Breast Cancer Incidence Rates. *International journal of cancer. Journal international du cancer* **132**, 1918–1926 (2013). URL https://www.ncbi.nlm.nih.gov/pmc/articles/PMC3553266/.

[26] Bray, F. *et al.* Global cancer statistics 2022: GLOBOCAN estimates of incidence and mortality worldwide for 36 cancers in 185 countries. *CA: A Cancer Journal for Clinicians* **74**, 229–263 (2024). URL https://onlinelibrary.wiley.com/doi/abs/10.3322/caac.21834. _eprint: https://onlinelibrary.wiley.com/doi/pdf/10.3322/caac.21834.

[27] Karaman, M. W. *et al.* A quantitative analysis of kinase inhibitor selectivity. *Nature Biotechnology* **26**, 127–132 (2008).

[28] Lories, R. J. & Luyten, F. P. The bone–cartilage unit in osteoarthritis. *Nature Reviews Rheumatology* **7**, 43–49 (2011). URL https://www.nature.com/articles/nrrheum.2010.197. Publisher: Nature Publishing Group.

[29] Jura-Półtorak, A., Szeremeta, A., Olczyk, K., Zoń-Giebel, A. & Komosińska-Vassev, K. Bone Metabolism and RANKL/OPG Ratio in Rheumatoid Arthritis Women Treated with TNF-α Inhibitors. *Journal of Clinical Medicine* **10**, 2905 (2021). URL https://www.ncbi.nlm.nih.gov/pmc/articles/PMC8267676/.

[30] Shangguan, L., Ding, M., Wang, Y., Xu, H. & Liao, B. Denosumab ameliorates osteoarthritis by protecting cartilage against degradation and modulating subchondral bone remodeling. *Regenerative Therapy* **27**, 181–190 (2024).

[31] Nakashima, T., Wada, T. & Penninger, J. M. RANKL and RANK as novel therapeutic targets for arthritis. *Current Opinion in Rheumatology* **15**, 280–287 (2003).

[32] Lu, J. *et al.* Current comprehensive understanding of denosumab (the RANKL neutralizing antibody) in the treatment of bone metastasis of malignant tumors, including pharmacological mechanism and clinical trials. *Frontiers in Oncology* **13**, 1133828 (2023). URL https://www.ncbi.nlm.nih.gov/pmc/articles/PMC9969102/.

[33] Kjolby, M. *et al.* Sort1, encoded by the cardiovascular risk locus 1p13.3, is a regulator of hepatic lipoprotein export. *Cell Metabolism* **12**, 213–223 (2010).

[34] Musunuru, K. *et al.* From noncoding variant to phenotype via SORT1 at the 1p13 cholesterol locus. *Nature* **466**, 714–719 (2010). URL https://www.ncbi.nlm.nih.gov/pmc/articles/PMC3062476/.

[35] Bycroft, C. *et al.* The UK Biobank resource with deep phenotyping and genomic data. *Nature* **562**, 203–209 (2018). URL https://www.nature.com/articles/s41586-018-0579-z. Publisher: Nature Publishing Group.

[36] Haller, T., Kals, M., Esko, T., Mägi, R. & Fischer, K. RegScan: a GWAS tool for quick estimation of allele effects on continuous traits and their combinations. *Briefings in Bioinformatics* **16**, 39–44 (2015). URL https://doi.org/10.1093/bib/bbt066.

Supplementary Figures

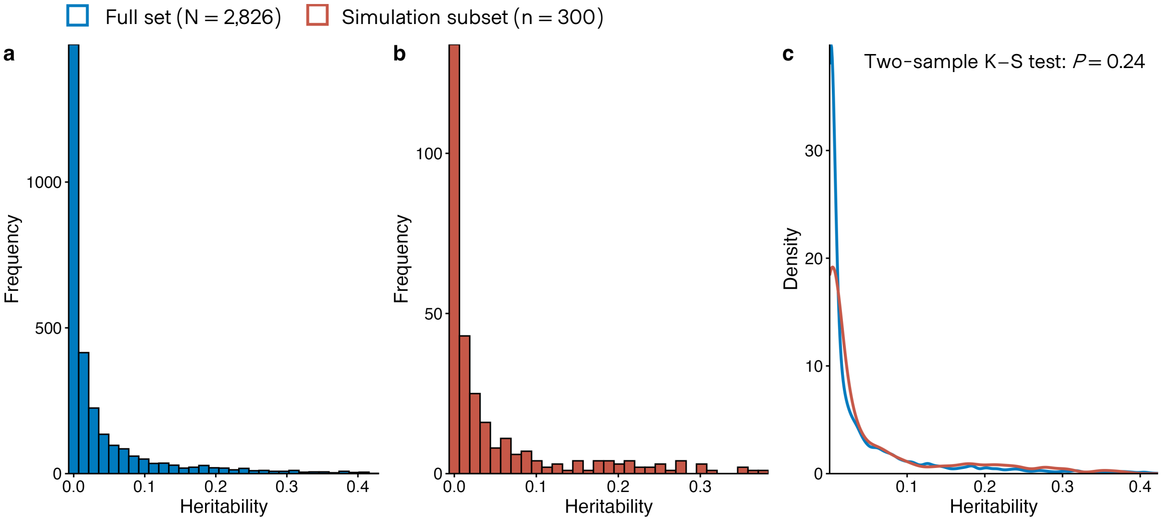

Supplementary Figure 1. Distribution of single SNP cis heritability estimates for plasma protein. The empirical distribution of the estimated heritability for the lead (top) variant in each cis pQTL region. (a) Histogram of all 2,826 cis pQTL regions. (b) Histogram of a random subset of 300 cis pQTL regions drawn without replacement so that their heritability spectrum mirrors that of the full set. (c) Kernel density overlays of the complete (blue) and subset (red) distributions. The P-value was derived by a two sample Kolmogorov–Smirnov test, confirming that no systematic bias was introduced by down sampling.

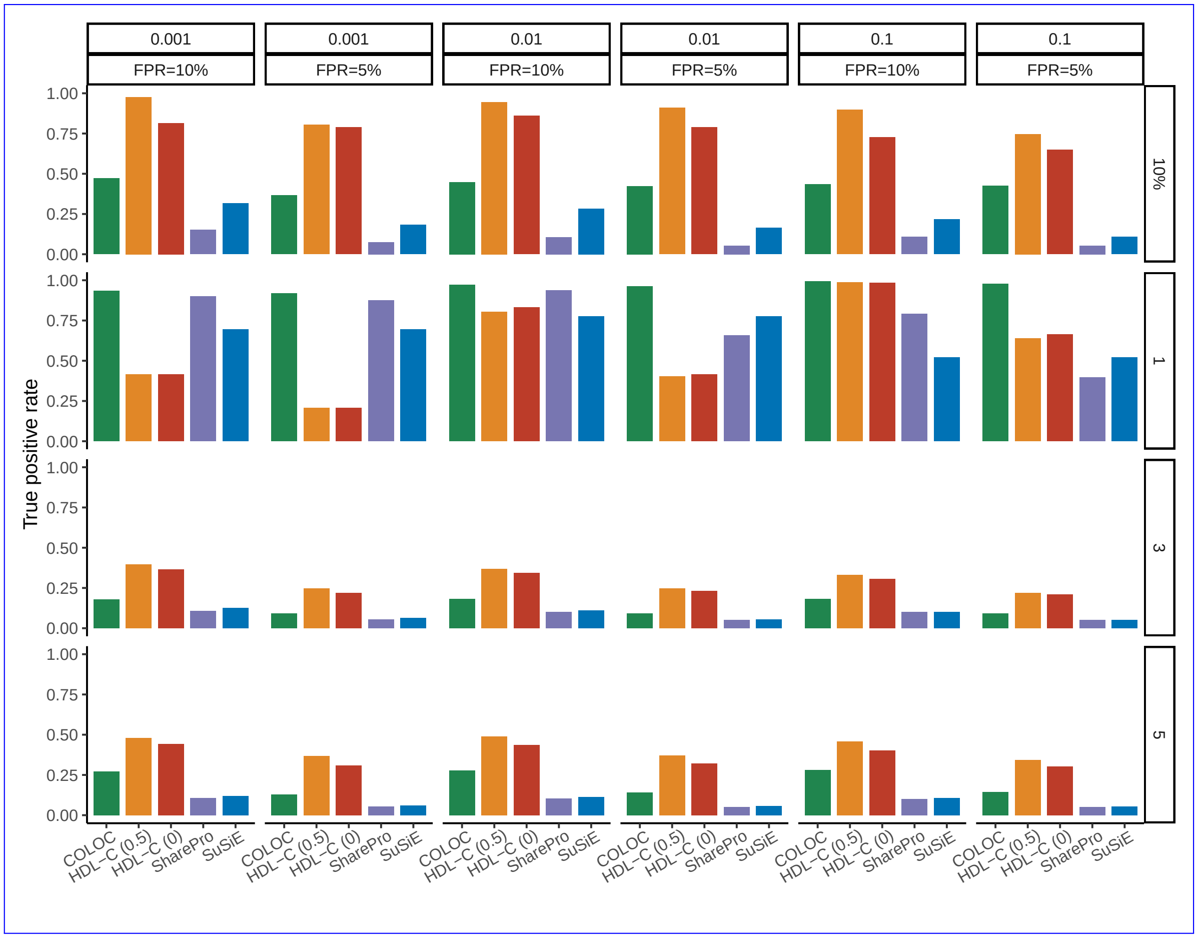

Supplementary Figure 2. True positive rate at fixed false positive rate thresholds. TPR is summarized at FPR = 5% and 10% for different numbers of causal SNPs (10%, 1, 3, 5) and heritability levels (0.001, 0.01, 0.1).

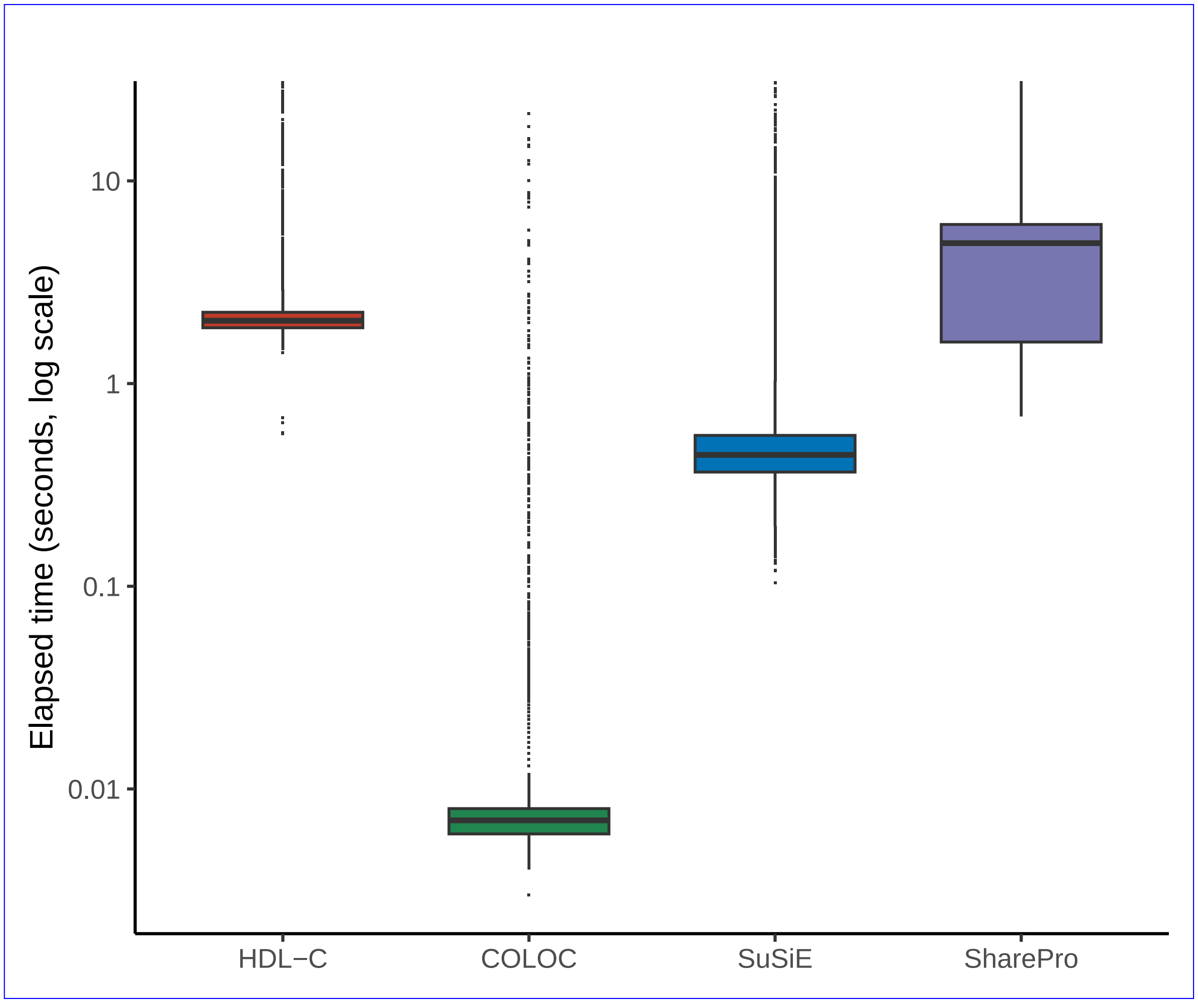

Supplementary Figure 3. Distributions of per-locus execution time of different methods. Boxplots of per-locus execution time ($\log_{10}$ seconds) across 50 simulation replicates for 300 cis-pQTL regions (i.e., $n = 15{,}000$ replicates per method). The central line marks the median and the box spans the inter-quartile range (IQR); whiskers extend to $1.5 \times$ IQR.

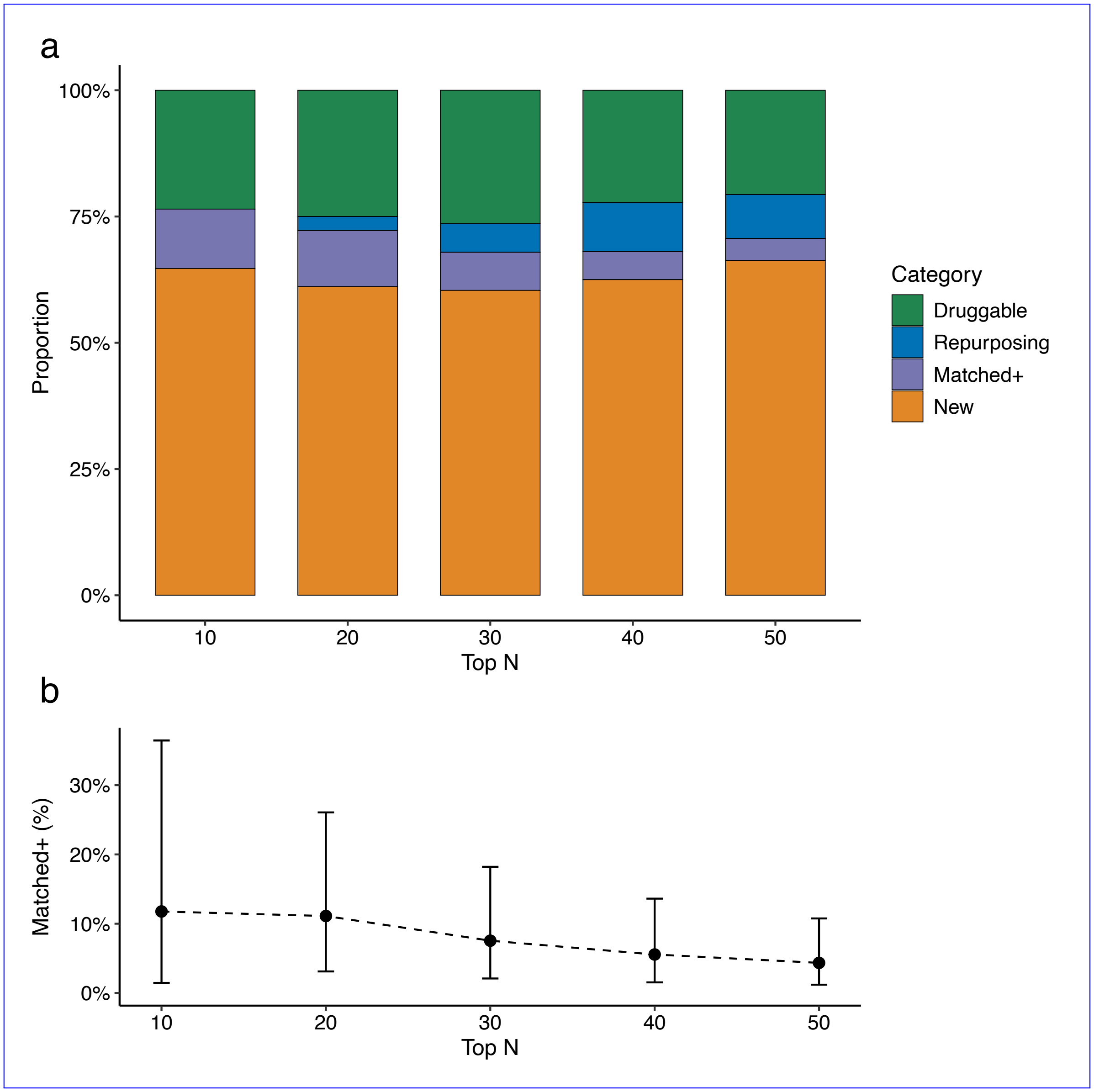

Supplementary Figure 4. Composition and enrichment across top-N thresholds. **(a)** Stacked proportions of category assignments—Druggable, Repurposing, Matched+, Matched−, and New—for the top-N HDL-C reported protein–disease pairs ($N \in \{10, 20, 30, 40, 50\}$). Proportions are computed as *Count*/*Total*, where *Total* is the number of unique pairs at each N. **(b)** The fraction of Matched+ pairs is shown with 95% confidence intervals, which provide binomially valid coverage for the underlying success proportion.

Supplementary Tables

Supplementary Table 1. The AUC values ~~and *P*-values~~ of HDL-C, COLOC, ~~and~~ SuSiE, and SharePro in simulation analyses. **Method**: Specifies the method used (HDL-C, COLOC, or SuSiE); **h11**: The simulated heritability level for the disease trait; **rg_threshold**: The threshold for the true genetic correlation used to define colocalization; **AUC**: The area under the receiver operating characteristic curve, reflecting the method's ability to distinguish between colocalized and non-colocalized regions; **CausalSNPs**: Denotes the simulation scenario—either a single causal SNP or multiple causal SNPs per region.

Supplementary Table 2. Description of 200 ICD-10 coded diseases. This table provides detailed information for 200 disease phenotypes based on ICD-10 codes used in the UK Biobank analysis. **phenotype**: ICD-10 code corresponding to each disease; **description**: Full description of the disease; **variable_type**: Indicates whether the phenotype is categorical or numerical; **source**: Source of the phenotype definition (ICD-10); **n_non_missing**: Number of individuals with non-missing phenotype data; **n_missing**: Number of individuals with missing phenotype data; **n_controls**: Number of individuals without the disease; **n_cases**: Number of individuals diagnosed with the disease.

Supplementary Table 3. Description of 2,826 proteins from the UK Biobank Pharma Proteomics Project. This table summarizes annotations and summary association statistics for 2,826 plasma proteins profiled in the UK Biobank Pharma Proteomics Project (UKB-PPP), used in cis-pQTL analyses. **UKBPPP_ProteinID**: Unique identifier of the protein, formatted as *HGNC.symbol_UniProt_OID_Panel_chr.Position*.
CHR, POS19, POS38: Chromosome and position of the top associated SNP in GRCh37 (POS19) and GRCh38 (POS38) coordinates; **REF, ALT**: Reference and alternative alleles for the top SNP; **rsid**: dbSNP identifier of the top SNP; **BETA, SE**: Estimated effect size and standard error from cis-pQTL association testing; **ALTFREQ**: Frequency of the alternative allele; **N**: Sample size used in the association analysis; **Z**: Z-score, computed as the ratio of BETA to SE; $\mathbf{h^2}$: SNP heritability of the top variant in each cis-region.

Supplementary Table 4. ~~Colocalization~~ Top 50 results ~~from HDL-C, COLOC, and SuSiE~~ of rediscovery analysis across ~~sex-stratified~~ methods and sex-specific UK Biobank populations. This Excel file contains the top 50 significant colocalization analysis results between 2,826 proteins and 200 ICD-10 coded disease traits, stratified by sex. Results were obtained using ~~three~~ four methods:

HDL-C, COLOC, ~~and SuSiE~~SuSiE, and SharePro. Each method was applied separately to the male and female datasets from the UK Biobank. The ~~file contains six sheets~~

~~**HDL_male**~~**threshold** ~~and **HDL_female**: Local genetic correlation estimates from HDL-C, applied to the male and female datasets, respectively.~~ denotes the training-derived significance cutoff corresponding to the 50th-ranked signal; **measuretest** provides the value of the same ranking statistic in the opposite-sex test cohort; and **rediscovered** indicates whether the locus surpasses the training threshold in the test dataset. The ~~**COLOC_male**~~**Method** and ~~**COLOC_female**~~**Direction** ~~: Posterior probabilities of five hypotheses estimated by COLOC, based on sex-stratified GWAS summary statistics. **SuSiE_male** and **SuSiE_female**: Fine-mapped colocalization results using the SuSiE regression model, reporting credible sets and posterior support for shared causal variants in males and females.~~ columns specify the statistical framework used (HDL-C, HDL-L, Coloc, SuSiE, or SharePro) and the direction of replication.

Supplementary Table 5. Top 50 significant protein–disease associations from HDL-C analysis and cross-referencing with DrugBank. This table summarizes results from an extended HDL-C(0) analysis in which we investigated the top 50 significant protein–disease associations separately in male and female cohorts. The combined set included 92 unique protein–disease pairs. Each entry includes local genetic correlation estimates and inference statistics ~~from~~ for HDL-C, ~~alongside DrugBank annotation for~~ along with DrugBank annotations for the corresponding protein targets. The file contains four sheets: Matched, druggable,re-purposing, and new.

uniprot, **protein**: UniProt accession and gene/protein name of the cis-pQTL.

phenotype, **description.x**: ICD-10 code and disease description.

Heritability_1, **Heritability_2**: Local SNP heritability estimates for the protein and disease traits, respectively.

Genetic_Covariance, **Genetic_Correlation**: Local genetic covariance and correlation estimates from HDL-C.

Lower_bound_rg, **Upper_bound_rg**, **P**: Likelihood-based confidence interval and P-value for local genetic correlation.

Gender: Indicates whether the result is from the male or female cohort.

drugbank_id, **name**, **description.y**: DrugBank ID, compound name, and description of known compounds targeting the protein.

known_action, **gene_name**, **cellular_location**: Drug–target interaction metadata and protein localization from DrugBank.
