# Supplementary material for: High-definition likelihood inference of genetic colocalization reveals protein biomarkers for human complex diseases
Source: Gigascience. 2026 Jan 23;15:giaf155. doi: 10.1093/gigascience/giaf155 (PMC12916012; doi:10.1093/gigascience/giaf155)
Supplement: giaf155_Reviewer_1_Report_original_submission — Mingxuan Cai -- 6/18/2025 [file giaf155_reviewer_1_report_original_submission.pdf]

Reviewer Report

Title: High-definition likelihood inference of colocalization reveals protein biomarkers for human complex diseases

Version: Original Submission **Date:** 6/18/2025

Reviewer name: Mingxuan Cai

Reviewer Comments to Author:

This paper presents HDL-C (High-Definition Likelihood for Colocalization), a new method for genetic colocalization analysis that aims to identify shared genetic architecture between traits by leveraging regional genetic correlations. The authors demonstrate that HDL-C outperforms existing methods like COLOC and SuSiE in detecting genetic colocalization through both simulation studies and real data applications using UK Biobank proteomics and disease data.The study has several notable strengths. The research addresses a practically important problem in human genetics, as identifying protein biomarkers through colocalization analysis has direct implications for therapeutic target discovery. The real data analysis is particularly compelling, with the identification of 92 unique protein-disease combinations and their systematic cross-referencing with DrugBank to reveal validated drug targets, repurposing opportunities, and novel therapeutic candidates. The application to sex-stratified cohorts and the rediscovery rate validation approach provide convincing evidence of the method's robustness. The writing is clear and the biological interpretations are well-grounded.However, the simulation studies are incomplete, comparing only against COLOC and SuSiE while omitting other established colocalization methods. Additionally, some simulation results contradict theoretical expectations and require better explanation. The following are specific statements of the problems:1. HDL-C appears to be a direct extension of HDL-L. The validity of proposed method is based on the assumption that colocalized loci have substantial genetic correlation. It seems more straightforward if simply testing the significance of genetic covariance (e.g., with HDL-L, LAVA, or superGNOVA). Can the authors clarify the necessity to use large-genetic correlation condition and empirically compare the above methods that directly test genetic covariance?2. The simulation setting with multiple causal SNPs scenario is unrealistic and unfair. Using 10% SNPs as causal could favor the correlation-based method. The number of causal cis-pQTL SNPs is usually less than 5 in practice.3. What is the r_0 for HDL-C in Figure1a? Was it set at the true genetic correlation threshold?4. In practice, is there a guidance on how to choose r_0? Is the proposed method robust to the setting of r_0?5. The simulation comparison is limited to only COLOC and SuSiE. While COLOC represents the classic colocalization approach and SuSiE focuses primarily on fine-mapping, the authors should include established colocalization methods (e.g., eCAVIAR, fastENLOC, sharepro) to provide a more comprehensive evaluation. Furthermore, the results presentation focuses solely on AUC values. Readers would benefit from seeing specific true positive rates and false positive rates at various threshold settings to better understand method performance characteristics.6. In the single causal SNP scenario, SuSiE performs best while COLOC performs worst, which contradicts theoretical expectations since SuSiE is designed for multiple causal variants while COLOC assumes a single causal variant per region. This unexpected result requires detailed analysis and discussion in the

main text to help readers understand the underlying reasons.7. The paper lacks discussion of computational requirements and scalability compared to existing methods, which is important for practical implementation.8. While the sex-stratified validation is interesting, the authors should clarify whether this design adequately addresses potential population stratification that might inflate apparent replication rates.

Level of Interest

Please indicate how interesting you found the manuscript: Choose an item.

Quality of Written English

Please indicate the quality of language in the manuscript: Choose an item.

Declaration of Competing Interests

Please complete a declaration of competing interests, considering the following questions:

- Have you in the past five years received reimbursements, fees, funding, or salary from an organisation that may in any way gain or lose financially from the publication of this manuscript, either now or in the future?
- Do you hold any stocks or shares in an organisation that may in any way gain or lose financially from the publication of this manuscript, either now or in the future?
- Do you hold or are you currently applying for any patents relating to the content of the manuscript?
- Have you received reimbursements, fees, funding, or salary from an organization that holds or has applied for patents relating to the content of the manuscript?
- Do you have any other financial competing interests?
- Do you have any non-financial competing interests in relation to this paper?

If you can answer no to all of the above, write 'I declare that I have no competing interests' below. If your reply is yes to any, please give details below.

I declare that I have no competing interests

I agree to the open peer review policy of the journal. I understand that my name will be included on my report to the authors and, if the manuscript is accepted for publication, my named report including any attachments I upload will be posted on the website along with the authors' responses. I agree for my report to be made available under an Open Access Creative Commons CC-BY license (http://creativecommons.org/licenses/by/4.0/). I understand that any comments which I do not wish to be included in my named report can be included as confidential comments to the editors, which will not be published.

Choose an item.

To further support our reviewers, we have joined with Publons, where you can gain additional credit to further highlight your hard work (see: https://publons.com/journal/530/gigascience). On publication of this paper, your review will be automatically added to Publons, you can then choose whether or not to claim your Publons credit. I understand this statement.

Yes Choose an item.
