# Supplementary material for: High-definition likelihood inference of genetic colocalization reveals protein biomarkers for human complex diseases
Source: Gigascience. 2026 Jan 23;15:giaf155. doi: 10.1093/gigascience/giaf155 (PMC12916012; doi:10.1093/gigascience/giaf155)
Supplement: giaf155_Reviewer_2_Report_original_submission — Jie Zheng -- 8/20/2025 [file giaf155_reviewer_2_report_original_submission.pdf]

Reviewer Report

Title: High-definition likelihood inference of colocalization reveals protein biomarkers for human complex diseases

Version: Original Submission **Date:** 8/20/2025

Reviewer name: Jie Zheng

Reviewer Comments to Author:

This manuscript presents a novel colocalization analysis method based on a conditional likelihood framework. The approach is innovative, and the authors systematically benchmark the method against several widely used colocalization methods using both simulated and real-world data. Furthermore, the study convincingly demonstrates the applicability of the proposed method in practical research settings, providing valuable evidence for drug development across multiple diseases. Overall, the manuscript is of high quality and scientific relevance, with only a few points requiring further clarification; specific comments are as below:1. Line 94-95, when the condition r_0>0 and ã€–|r Ì‚ã€—_G |>r_0 are satisfied, the scenario of h_12=0 does not seem to be valid, which raises some uncertainty regarding the computation of the conditional likelihood function. To improve clarity for readers, I recommend that the authors provide further explanation on how this formula is derived and applied in such cases.2. It would be helpful if the authors could further compare the robustness or power of detection of HDL-C relative to other methods in the presence of noise within a region, such as when some SNPs exhibit heterogeneity.3. The authors selected the top 50 significant protein-disease pairs in their analysis. Could the authors clarify the rationale for choosing this threshold? If 50 pairs were randomly drawn from all significant results, would HDL-C still demonstrate greater consistency or robustness compared with other methods? Furthermore, would the proportion of results that can be validated in real-world settings differ from those observed when focusing only on the top 50?

Level of Interest

Please indicate how interesting you found the manuscript: Choose an item.

Quality of Written English

Please indicate the quality of language in the manuscript: Choose an item.

Declaration of Competing Interests

Please complete a declaration of competing interests, considering the following questions:

- Have you in the past five years received reimbursements, fees, funding, or salary from an organisation that may in any way gain or lose financially from the publication of this manuscript, either now or in the future?

- Do you hold any stocks or shares in an organisation that may in any way gain or lose financially from the publication of this manuscript, either now or in the future?
- Do you hold or are you currently applying for any patents relating to the content of the manuscript?
- Have you received reimbursements, fees, funding, or salary from an organization that holds or has applied for patents relating to the content of the manuscript?
- Do you have any other financial competing interests?
- Do you have any non-financial competing interests in relation to this paper?

If you can answer no to all of the above, write 'I declare that I have no competing interests' below. If your reply is yes to any, please give details below.

No declare of interest

I agree to the open peer review policy of the journal. I understand that my name will be included on my report to the authors and, if the manuscript is accepted for publication, my named report including any attachments I upload will be posted on the website along with the authors' responses. I agree for my report to be made available under an Open Access Creative Commons CC-BY license (http://creativecommons.org/licenses/by/4.0/). I understand that any comments which I do not wish to be included in my named report can be included as confidential comments to the editors, which will not be published.

Choose an item.

To further support our reviewers, we have joined with Publons, where you can gain additional credit to further highlight your hard work (see: https://publons.com/journal/530/gigascience). On publication of this paper, your review will be automatically added to Publons, you can then choose whether or not to claim your Publons credit. I understand this statement.

Yes Choose an item.
